# Supplementary material for: Dynamic mechanostereochemical switching of a co-conformationally flexible [2]catenane controlled by specific ionic guests
Source: Nat Commun. 2024 Mar 4;15:1952. doi: 10.1038/s41467-024-46099-w (PMC10909852; doi:10.1038/s41467-024-46099-w)
Supplement: Supplementary file 1 — Supplementary Information [file 41467_2024_46099_MOESM1_ESM.pdf]

Supplementary Information

Dynamic mechanostereochemical switching of a co-conformationally flexible [2]catenane controlled by specific ionic guests

Yueliang Yao,[1] Yuen Cheong Tse,[1] Samuel Kin-Man Lai,[1] Yixiang Shi,[1] Kam-Hung Low[1] and Ho Yu Au-Yeung[1,2,*]

[1]Department of Chemistry, The University of Hong Kong, Hong Kong, China

[2]State Key Laboratory of Synthetic Chemistry and HKU-CAS Joint Laboratory on New Materials, The University of Hong Kong, Hong Kong, China

*Correspondence: hoyuay@hku.hk

Supplementary Methods

Synthesis

General. All reagents (purity ≥98% unless otherwise noted) were purchased from commercial suppliers (J&K, Sigma-Aldrich, TCI, Aladdin, Energy, Macklin and Dkmchem) and used without further purification unless otherwise noted. All the solvents were of analytical grade (ACI Labscan and DUKSAN Pure Chemicals). Deuterated solvents $CDCl_3$ and DMSO-d_6 were purchased from Energy (≥99.8 atom% D). **Bpy-OH**[1] and **Bpy-p-CHO**[2] were synthesized according to literature procedures. Thin layer chromatography (TLC) was performed on silica gel 60 F254 (Merck, Germany, Aluminium sheet) and column chromatography was carried out using silica gel 60F (Silicycle, Canada). HR-ESI-MS were obtained from a Bruker Impact II Ultra-High Resolution QTOF mass spectrometer. NMR spectra were recorded on Bruker DPX spectrometers with working frequencies of 400 MHz, 500 MHz or 600 MHz for ^{1}H, and 100 MHz or 125 MHz for ^{13}C, respectively. Chemical shifts were reported in ppm and referenced to solvent residues (for ^{1}H: $CDCl_3$: δ = 7.26 ppm, DMSO-d_6: δ = 2.50 ppm, CD_3CN: δ = 1.94 ppm; for ^{13}C: $CDCl_3$: δ = 77.16 ppm, DMSO-d_6: δ = 39.52 ppm).

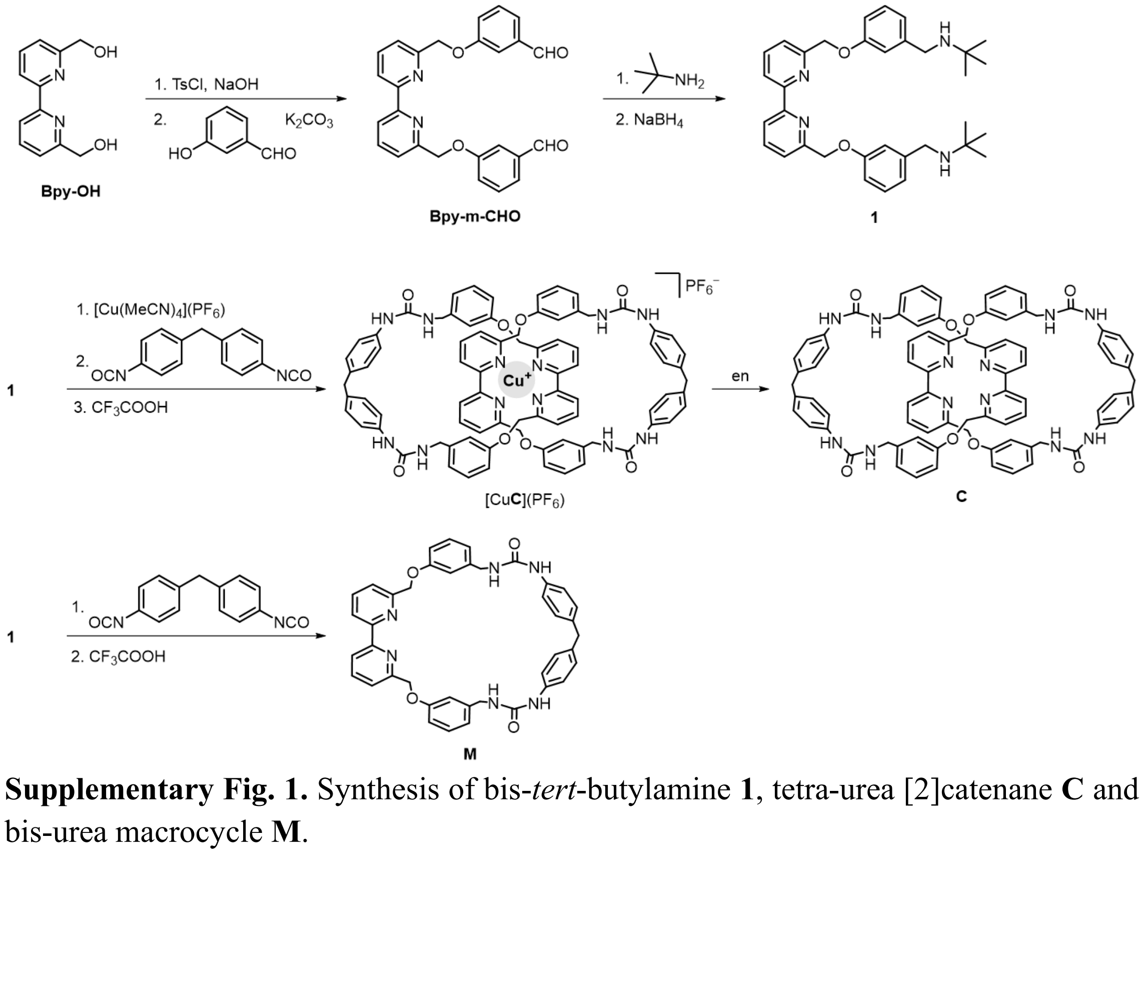

Supplementary Fig. 1. Synthesis of bis-*tert*-butylamine **1**, tetra-urea [2]catenane **C** and bis-urea macrocycle **M**.

Synthesis of **Bpy-m-CHO**. A mixture of **Bpy-OH** (1.08 g, 5.0 mmol) and NaOH (0.60 g, 15 mmol) in THF/H_2O (*v/v* 5:1, 30 mL) was stirred at room temperature for 10 min. The mixture was cooled to 0 °C in an ice bath and a solution of 4-toluenesulfonyl chloride (2.29 g, 12 mmol) in THF (30 mL) was slowly added over 10 min and stirred for 4 h at room temperature. The solvent was removed by a rotary evaporator and the residue was dissolved in CH_2Cl_2 (50 mL), washed with water and brine, dried over Na_2SO_4, filtered and concentrated to afford a yellow oil. The yellow oil was diluted with MeCN (50 mL), and 3-hydroxybenzaldehyde (1.46 g, 12 mmol) and K_2CO_3 (2.07 g, 15 mmol) were added. The mixture was heated to reflux for overnight. Insoluble materials were removed by filtration and the solvent was removed by a rotary evaporator. The residue was dissolved in CH_2Cl_2 (100 mL), washed with dilute NH_4Cl (aq), water and brine, dried over Na_2SO_4, filtered and concentrated to afford a dark oil, which was purified on a silica column (CH_2Cl_2/EtOAc, *v/v* = 10:1) to afford the **Bpy-m-CHO** as a white solid. Yield = 1.04 g, 49%. ^{1}H NMR (500 MHz, DMSO-d_6, 298 K) δ 9.99 (s, 2H), 8.35 (d, J = 7.8 Hz, 2H), 8.02 (t, J = 7.8 Hz, 2H), 7.64–7.60 (m, 4H), 7.58–7.54 (m, 4H), 7.45–7.41 (m, 2H), 5.39 (s, 4H). ^{13}C{^{1}H} NMR (125 MHz, DMSO-d_6, 298 K) δ 192.9, 158.7, 156.1, 154.6, 138.3, 137.7, 130.5, 122.9, 122.2, 121.7, 119.8, 114.2, 70.6. ESI-HRMS (+ve) found: 425.1493; calcd. for $C_{26}H_{21}N_2O_4^+$ [M+H]$^+$: 425.1496.

Synthesis of **1**. A mixture of **Bpy-m-CHO** (0.42 g, 1.0 mmol), *tert*-butylamine (0.73 g, 10 mmol) and piperidine (0.5 mL) in MeOH/$CHCl_3$ (*v/v* 1:1, 30 mL) was heated at 50 °C for overnight. The reaction was cooled to 0 °C in an ice bath and $NaBH_4$ (0.09 g, 2.5 mmol) was added. The mixture was stirred at room temperature for 30 min, and the solvents were removed by a rotary evaporator. The residue was partitioned between aq. HCl (pH ~3, 100 mL) and $CHCl_3$ (50 mL). The aqueous layer was separated, washed with $CHCl_3$ (2 × 20 mL) and added saturated K_2CO_3 (5 mL) to give a white mixture and was extracted with CH_2Cl_2 (2 × 50 mL). The organic layer was then washed with dilute aq. K_2CO_3, water and brine, dried over Na_2SO_4, filtered and concentrated to afford a pale-yellow solid as the product. Yield = 0.45 g, 84%. ^{1}H NMR (500 MHz, $CDCl_3$, 298 K) δ 8.34 (d, J = 7.8 Hz, 2H), 7.84 (t, J = 7.8 Hz, 2H), 7.56 (d, J = 7.6 Hz, 2H), 7.23 (t, J = 7.9 Hz, 2H), 7.06 (s, 2H), 6.95 (d, J = 7.5 Hz, 2H), 6.89 (dd, J = 8.2, 2.0 Hz, 2H), 5.30 (s, 4H), 3.71 (s, 4H), 1.16 (s, 18H). ^{13}C{^{1}H} NMR (125 MHz, $CDCl_3$, 298 K) δ 158.8, 157.2, 155.6, 143.5, 137.8, 129.6, 121.4, 121.1, 120.1, 115.0, 113.2, 70.9, 50.8, 47.3, 29.3. ESI-HRMS (+ve) found: 539.3381; calcd. for $C_{34}H_{43}N_4O_2^+$ [M+H]$^+$: 539.3381.

Synthesis of [Cu**C**](PF_6). Under an argon atmosphere, a mixture of **1** (108 mg, 0.20 mmol) and [Cu(MeCN)$_4$](PF_6) (37 mg, 0.10 mmol) in MeCN/$CHCl_3$ (*v/v* 1:1, 10 mL) was heated at 50 °C for 1 h. The red reaction mixture was cooled to room temperature and filtered to remove insoluble materials. Solvents were removed by a rotary evaporator to produce a red solid. The red solid was re-dissolved in dry 1,2-dichloroethane (50 mL) under an argon atmosphere and 4,4'-methylenediphenyl diisocyanate (62 mg, 0.25 mmol) was added. The reaction mixture was heated at 50 °C for overnight and cooled to room temperature. The product mixture was filtered and quickly added CF_3COOH (2 mL), and the mixture was stirred for 6 h at room temperature. Solvents were removed by a rotary evaporator to afford a dark red waxy solid, which was re-dissolved in MeOH/$CHCl_3$ (*v/v* 1:1). Slow diffusion of diethyl ether to the solution gave [Cu**C**](PF_6) as an orange solid. Yield = 97 mg, 62%. ^{1}H NMR (600 MHz, DMSO-*d*$_6$, 298 K) δ 8.39 (s, 4H), 8.12 (dd, J = 7.4, 1.5 Hz, 4H), 7.28–7.22 (m, 16H), 7.06–7.03 (m, 12H), 6.78 (d, J = 7.6 Hz, 4H), 6.51–6.47 (m, 8H), 6.20 (s, 4H), 4.63 (s, 8H), 4.13 (d, J = 6.0 Hz, 8H), 3.73 (s, 4H). ^{13}C{^{1}H} NMR (125 MHz, DMSO-*d*$_6$, 298 K) δ 157.3, 155.3, 153.9, 151.0, 142.4, 138.5, 138.1, 135.8, 129.0, 128.5, 125.8, 121.9, 120.5, 118.5, 112.8, 112.4, 69.9, 42.5. ESI-HRMS (+ve) found: 1415.4874; calcd. for $C_{82}H_{72}N_{12}O_8Cu^+$ [M]$^+$: 1415.4887.

Synthesis of **C**. To a solution of [Cu**C**](PF_6) (97 mg, 0.062 mmol) in MeOH/$CHCl_3$ (*v/v* 1:1, 10 mL) was added 2 mL of ethylenediamine (30 mmol), and the mixture was stirred for 5 min. $CHCl_3$ (3 mL) and water (3 mL) were added, and the yellow organic phase was separated. Another aliquot of ethylenediamine (1 mL, 15 mmol) was added to the organic portion and stirred for another 5 min to give a colourless solution. $CHCl_3$ (3 mL) and water (3 mL) were added, and the organic phase was further washed by water (2 × 5 mL) and dried over Na_2SO_4. Insoluble materials were removed by filtration and the solvents were removed using a rotary evaporator to afford the pale-yellow solid. The crude product was washed with Et_2O/CH_2Cl_2 (*v/v* 5:1) and dried to give **C**. Yield = 55 mg, 66%. ^{1}H NMR (600 MHz, DMSO-*d*$_6$, 298 K) δ 8.25 (s, 4H), 7.57 (d, J = 7.8 Hz, 4H), 7.25–7.18 (m, 8H), 6.97–6.93 (m, 12H), 6.90 (s, 4H), 6.88–6.83 (m, 8H), 6.62 (d, J = 8.4 Hz, 8H), 6.52 (t, J = 6.1 Hz, 4H), 4.72 (s, 8H), 4.24 (d, J = 5.9 Hz, 8H). ^{13}C{^{1}H} NMR (125 MHz, DMSO-*d*$_6$, 298 K) δ 158.6, 155.3, 154.8, 153.9, 142.5, 137.8, 136.8, 134.3, 129.2, 128.2, 121.7, 119.8, 119.2, 117.6, 113.6, 112.4, 70.4, 42.4. ESI-HRMS (+ve) found: 677.2873; calcd. for $C_{82}H_{74}N_{12}O_8^{2+}$ [M+2H]$^{2+}$: 677.2871.

Synthesis of **M**. Under an argon atmosphere, a mixture of **1** (54 mg, 0.10 mmol) and 4,4'-methylenediphenyl diisocyanate (28 mg, 0.11 mmol) in dry 1,2-dichloroethane (50 mL) was heated at 50 °C for overnight. The reaction mixture was cooled to room temperature and filtered. To the filtrate was added CF_3COOH (2 mL) and the mixture was stirred for 6 h at room temperature. Solvents were removed by a rotary evaporator to afford a yellow solid, which was washed with $CHCl_3$ and dried to give a pale-yellow solid as the product. Yield = 54 mg, 80%. ^{1}H NMR (600 MHz, DMSO-d_6, 298 K) δ 8.45 (s, 2H), 8.18 (d, J = 7.7 Hz, 2H), 7.72 (t, J = 7.8 Hz, 2H), 7.50 (d, J = 7.6 Hz, 2H), 7.27–7.23 (m, 6H), 7.04 (d, J = 8.5 Hz, 4H), 6.96 (dd, J = 8.2, 2.2 Hz, 2H), 6.94 (s, 2H), 6.85 (d, J = 7.6 Hz, 2H), 6.55 (t, J = 6.2 Hz, 2H), 5.18 (s, 4H), 4.24 (d, J = 6.1 Hz, 4H), 3.73 (s, 2H). ^{13}C{^{1}H} NMR (125 MHz, DMSO-d_6, 298 K) δ 158.4, 156.1, 155.4, 154.6, 142.7, 138.3, 138.0, 134.8, 129.3, 128.7, 122.6, 119.8, 119.7, 118.0, 113.2, 112.8, 70.3, 42.4. ESI-HRMS (+ve) found: 677.2879; calcd. for $C_{41}H_{37}N_6O_4^+$ $[M+H]^+$: 677.2871.

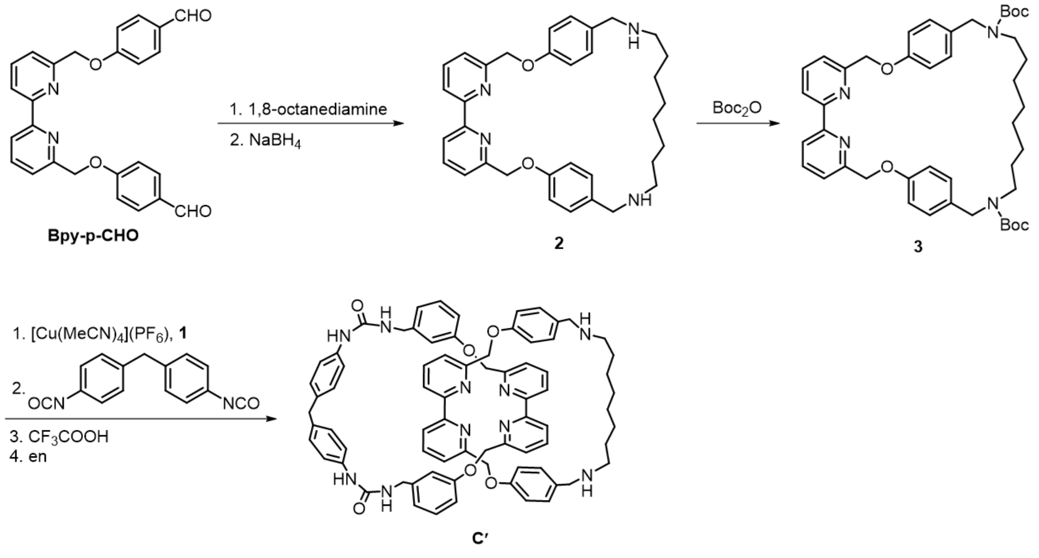

Supplementary Fig. 2. Synthesis of heteroleptic bis-urea [2]catenane **C′**.

Synthesis of **2**. A mixture of **Bpy-p-CHO** (106 mg, 0.25 mmol), 1,8-diaminooctane (40 mg, 0.28 mmol) and piperidine (0.5 mL) in MeOH/$CHCl_3$ (*v/v* 1:10, 200 mL) was heated at 50 °C for overnight. The reaction was cooled to 0 °C in an ice bath and $NaBH_4$ (19 mg, 0.50 mmol) was added. The mixture was stirred at room temperature for 30 min, and the solvents were removed by a rotary evaporator. The residue was dissolved in CH_2Cl_2 (100 mL), washed with water and brine, dried over Na_2SO_4, filtered and concentrated to afford a yellow oil, which was purified on a silica column (CH_2Cl_2/MeOH/Et_3N, *v/v/v* = 100:1:1) to give a white solid. Yield = 60 mg, 45%. ^{1}H NMR (500 MHz, $CDCl_3$, 298 K) δ 8.32 (d, J = 7.8 Hz, 2H), 7.79 (t, J = 7.8 Hz, 2H), 7.45 (d, J = 7.6 Hz, 2H), 7.14 (d, J = 8.5 Hz,

4H), 6.94 (d, J = 8.7 Hz, 4H), 5.36 (s, 4H), 3.70 (s, 4H), 2.49 (t, J = 7.1 Hz, 4H), 1.43 (s, 4H), 1.18 (s, 8H). $^{13}C\{^{1}H\}$ NMR (125 MHz, $CDCl_3$, 298 K) δ 157.4, 157.3, 155.5, 137.8, 129.5, 122.3, 120.3, 115.5, 71.2, 53.1, 48.6, 30.0, 29.6, 27.1. ESI-HRMS (+ve) found: 537.3244; calcd. for $C_{34}H_{41}N_4O_2^+$ $[M+H]^+$: 537.3224.

Synthesis of **3**. A solution of **2** (54 mg, 0.10 mmol) in CH_2Cl_2 (10 mL) was cooled to 0 °C in an ice bath and added a solution of di-*tert*-butyl dicarbonate (87 mg, 0.40 mmol) in CH_2Cl_2 (5 mL) over 2 min. The mixture was stirred at room temperature for overnight. The solvent was removed by a rotary evaporator and the residue was dissolved in CH_2Cl_2 (50 mL), washed with water and brine, dried over Na_2SO_4, filtered and concentrated to afford a pale-yellow oil, which was purified on a silica column (CH_2Cl_2/EtOAc, *v/v* = 50:1) to afford a colourless oil. Yield = 59 mg, 80%. ^{1}H NMR (500 MHz, $CDCl_3$, 298 K) δ 8.26 (s, 2H), 7.77 (t, J = 7.8 Hz, 2H), 7.43 (d, J = 7.6 Hz, 2H), 7.06 (s, 4H), 6.91 (d, J = 8.0 Hz, 4H), 5.36 (s, 4H), 4.31 (s, 4H), 2.94 (d, J = 34.6 Hz, 4H), 1.45 (s, 18H), 1.33 (s, 4H), 1.09 (s, 8H). $^{13}C\{^{1}H\}$ NMR (125 MHz, $CDCl_3$, 298 K) δ 157.5, 157.3, 155.6, 137.8, 131.1, 122.1, 120.2, 115.5, 71.0, 49.9, 49.5, 46.2, 29.3, 28.6, 27.0. ESI-HRMS (+ve) found: 737.4277; calcd. for $C_{44}H_{57}N_4O_6^+$ $[M+H]^+$: 737.4273.

Synthesis of **C′**. Under an argon atmosphere, a mixture of **3** (40 mg, 0.055 mmol), **1** (27 mg, 0.050 mmol) and $[Cu(MeCN)_4](PF_6)$ (20 mg, 0.055 mmol) in MeCN/$CHCl_3$ (*v/v* 1:1, 5 mL) was heated at 50 °C for 3 h. The red reaction mixture was cooled to room temperature, filtered to remove insoluble materials, and the solvents were removed by a rotary evaporator to produce a red solid. The red solid was re-dissolved in dry 1,2-dichloroethane (25 mL) under an argon atmosphere and 4,4'-methylenediphenyl diisocyanate (14 mg, 0.056 mmol) was added. The reaction mixture was heated at 50 °C for overnight and cooled to room temperature. The red solution was filtered and quickly added CF_3COOH (2 mL), and the mixture was stirred for 6 h at room temperature. Solvents were removed by a rotary evaporator, and the residue was re-dissolved in CH_2Cl_2 (10 mL). Ethylenediamine (2 mL, 30 mmol) was added, and the mixture was stirred for 5 min, followed by addition of CH_2Cl_2 (3 mL) and water (3 mL). The yellow organic phase was separated, and another aliquot of ethylenediamine (1 mL, 15 mmol) was added and stirred for another 5 min to give a colourless solution. Additional CH_2Cl_2 (3 mL) and water (3 mL) were added, and the organic phase was washed by water (2 × 5 mL). The solvent was removed using a rotary evaporator to afford the pale-yellow solid, which was washed with Et_2O and purified by preparative thin layer chromatography

(CH_2Cl_2/MeOH/Et_3N, *v/v/v* = 50:1:1) to give **C′**. Yield = 27 mg, 45%. ^{1}H NMR (400 MHz, DMSO-d_6, 298 K) δ 8.34 (s, 2H), 7.86 (d, J = 7.8 Hz, 2H), 7.63 (d, J = 7.6 Hz, 2H), 7.40 (t, J = 7.7 Hz, 2H), 7.33–7.27 (m, 4H), 7.23 (s, 2H), 7.11–7.04 (m, 6H), 7.01 (d, J = 8.5 Hz, 4H), 6.96 (d, J = 8.0 Hz, 4H), 6.86 – 6.81 (m, 4H), 6.78 (d, J = 7.7 Hz, 2H), 6.58 (d, J = 8.5 Hz, 4H), 6.49 (s, 2H), 5.13 (s, 4H), 4.74 (s, 4H), 4.24 (d, J = 5.8 Hz, 4H), 3.45 (s, 2H), 3.26 (s, 2H), 1.94 (s, 4H), 0.73 (s, 4H), 0.23 (s, 8H). ^{13}C{^{1}H} NMR (100 MHz, DMSO-d_6, 298 K) δ 158.8, 157.0, 155.4, 155.1, 154.6, 154.1, 142.7, 138.0, 137.4, 134.5, 129.8, 129.5, 128.6, 122.7, 122.4, 119.9, 119.8, 119.5, 117.5, 115.2, 113.8, 111.9, 70.6, 70.1, 55.0, 29.3, 28.8, 26.0. ESI-HRMS (+ve) found: 1213.6027; calcd. for $C_{75}H_{77}N_{10}O_6^+$ $[M+H]^+$: 1213.6022.

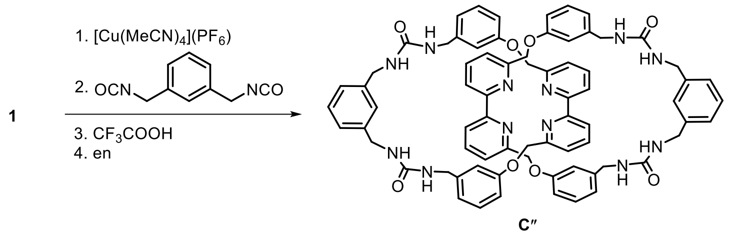

Supplementary Fig. 3. Synthesis of tetra-urea [2]catenane **C″**.

Synthesis of **C″**. Under an argon atmosphere, a mixture of **1** (54 mg, 0.10 mmol) and $[Cu(MeCN)_4](PF_6)$ (19 mg, 0.05 mmol) in MeCN/$CHCl_3$ (*v/v* 1:1, 10 mL) was heated at 50 °C for 1 h. The red reaction mixture was cooled to room temperature and filtered to remove insoluble materials. Solvents were removed by a rotary evaporator to produce a red solid. The red solid was re-dissolved in dry 1,2-dichloroethane (20 mL) under an argon atmosphere and *m*-xylylene diisocyanate (24 mg, 0.13 mmol) was added. The reaction mixture was heated at 50 °C for overnight and cooled to room temperature. The product mixture was filtered and quickly added CF_3COOH (2 mL), and the mixture was stirred for 6 h at room temperature. Solvents were removed by a rotary evaporator to afford a dark red waxy solid, which was re-dissolved in MeOH/$CHCl_3$ (*v/v* 1:1). Ethylenediamine (2 mL, 30 mmol) was added, and the mixture was stirred for 5 min, followed by addition of CH_2Cl_2 (3 mL) and water (3 mL). The yellow organic phase was separated, and another aliquot of ethylenediamine (1 mL, 15 mmol) was added and stirred for another 5 min to give a colourless solution. Additional CH_2Cl_2 (3 mL) and water (3 mL) were added, and the organic phase was washed by water (2 × 5 mL). The solvent was removed using a rotary evaporator to afford the pale-yellow solid, which was washed with Et_2O and purified by preparative thin layer chromatography (CH_2Cl_2/MeOH/Et_3N, *v/v/v* = 50:1:1) to give **C″**. Yield = 5 mg, 8%. ^{1}H NMR (500 MHz, DMSO-d_6, 298 K) δ

7.90 (d, J = 7.5 Hz, 4H), 7.68 (t, J = 7.6 Hz, 4H), 7.32 (d, J = 6.8 Hz, 4H), 7.15 (t, J = 7.6 Hz, 4H), 6.95–6.88 (m, 6H), 6.81 (d, J = 7.0 Hz, 4H), 6.75 (d, J = 7.5 Hz, 4H), 6.70 (s, 4H), 6.64 (s, 2H), 6.28 (s, 4H), 6.03 (s, 4H), 5.00 (s, 8H), 3.94 (s, 8H), 3.74 (s, 8H). $^{13}C\{^{1}H\}$ NMR (125 MHz, DMSO-d_6, 298 K) δ 158.2, 158.0, 156.3, 154.8, 141.7, 139.6, 137.7, 129.4, 127.9, 125.9, 125.7, 121.7, 120.2, 114.0, 113.0, 69.9, 43.3, 43.1. ESI-HRMS (+ve) found: 615.2703; calcd. for $C_{72}H_{70}N_{12}O_8^{2+}$ [M+2H]$^{2+}$: 615.2714.

Supplementary Discussion

1. MS Characterization

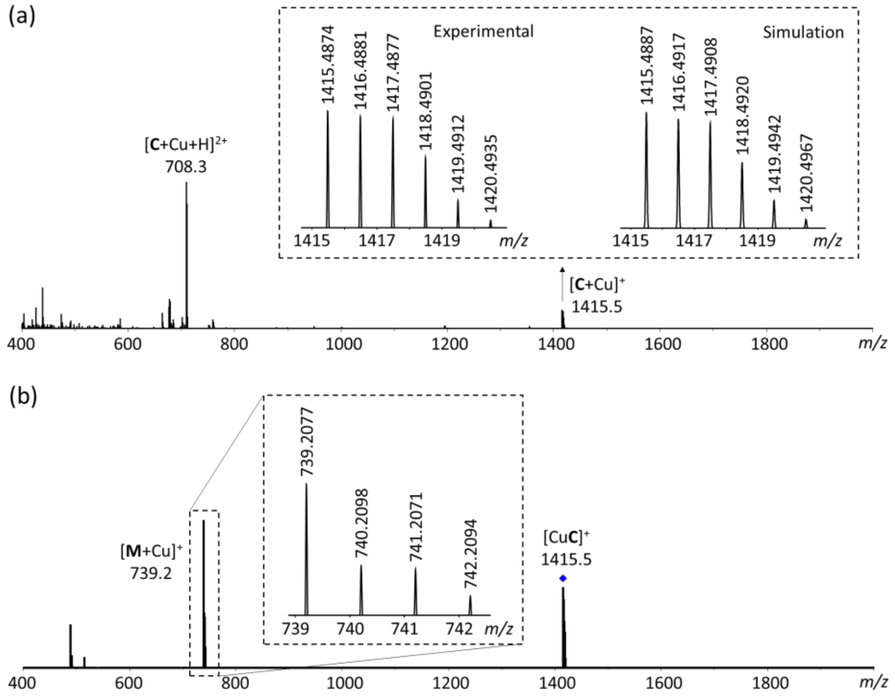

Supplementary Fig. 4. (a) ESI-MS (+ve) spectrum of [Cu**C**](PF_6) obtained from the templated synthesis (inset: isotopic distribution of the peak at *m/z* = 1415.5) and (b) MS/MS spectrum upon fragmentation of the peak at *m/z* = 1415.5.

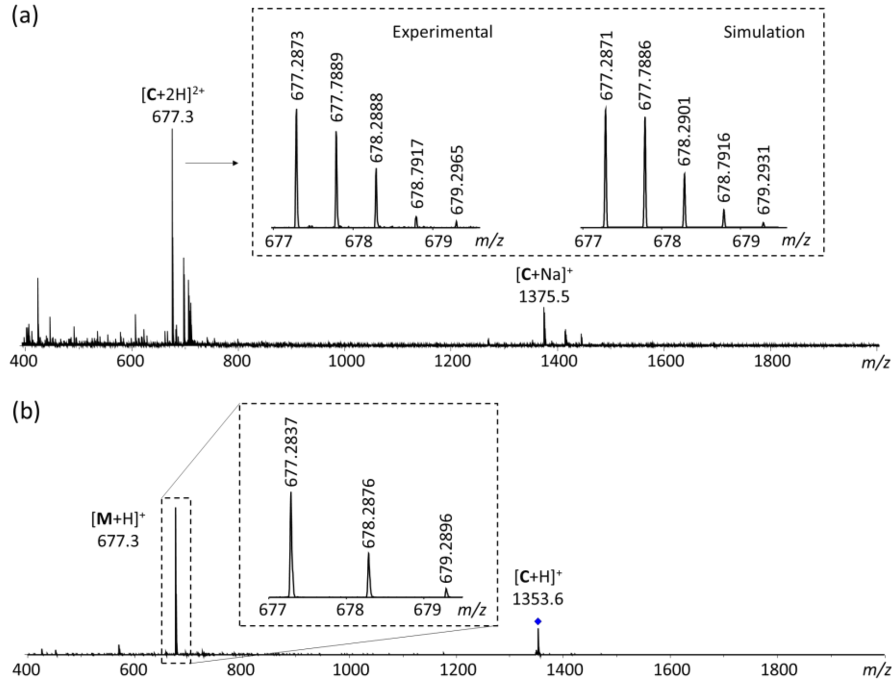

Supplementary Fig. 5. (a) ESI-MS (+ve) spectrum of **C** (inset: isotopic distribution of the peak at *m/z* = 677.3) and (b) MS/MS spectrum upon fragmentation of the peak at *m/z* = 1353.6.

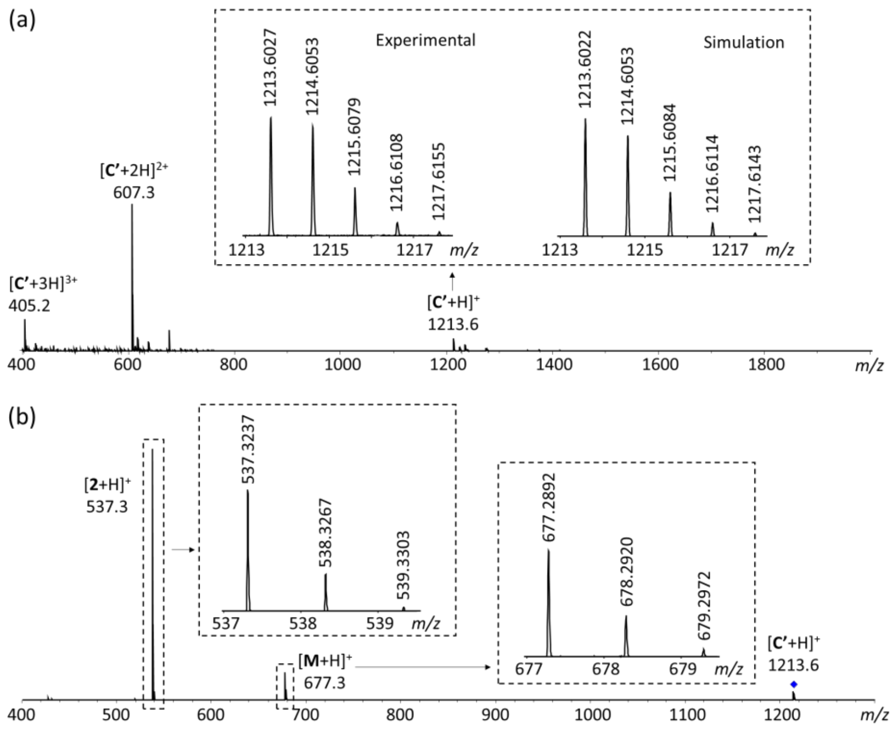

Supplementary Fig. 6. (a) ESI-MS (+ve) spectrum of **C′** (inset: isotopic distribution of the peak at *m/z* = 1213.6) and (b) MS/MS spectrum upon fragmentation of the peak at *m/z* = 1213.6.

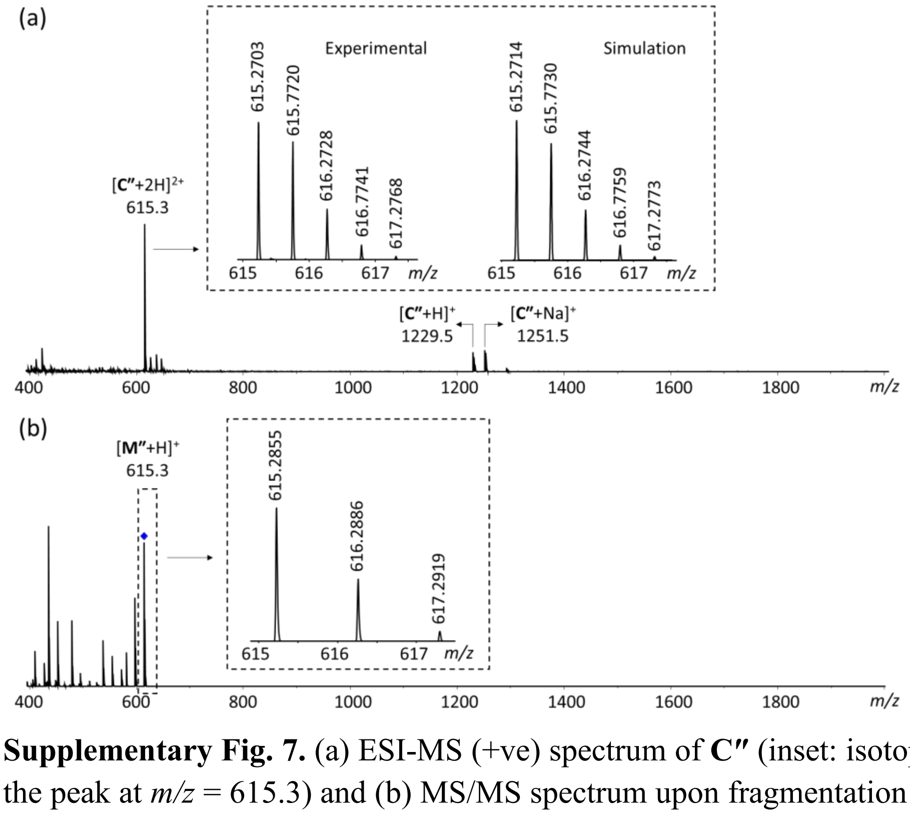

Supplementary Fig. 7. (a) ESI-MS (+ve) spectrum of **C″** (inset: isotopic distribution of the peak at *m/z* = 615.3) and (b) MS/MS spectrum upon fragmentation of the [**C″**+2H]$^{2+}$ peak at *m/z* = 615.3.

2. NMR Characterization

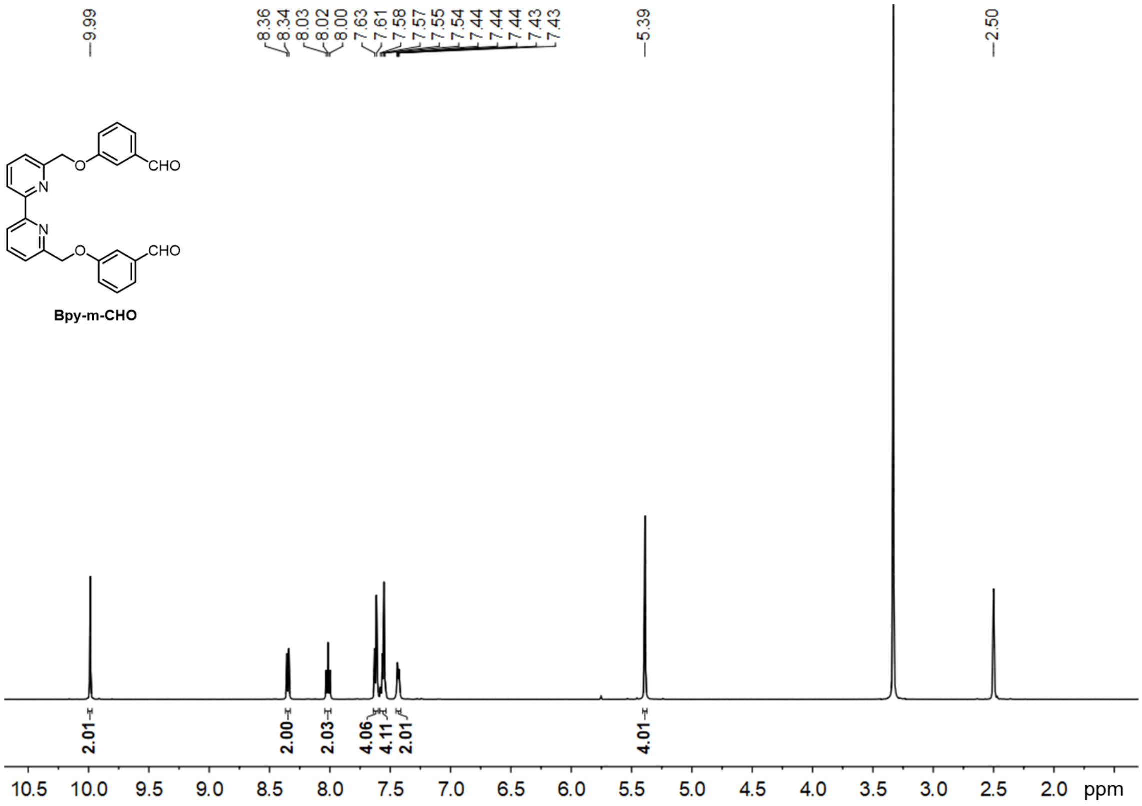

Supplementary Fig. 8. ^{1}H NMR (500 MHz, DMSO-d_6, 298 K) of **Bpy-m-CHO.**

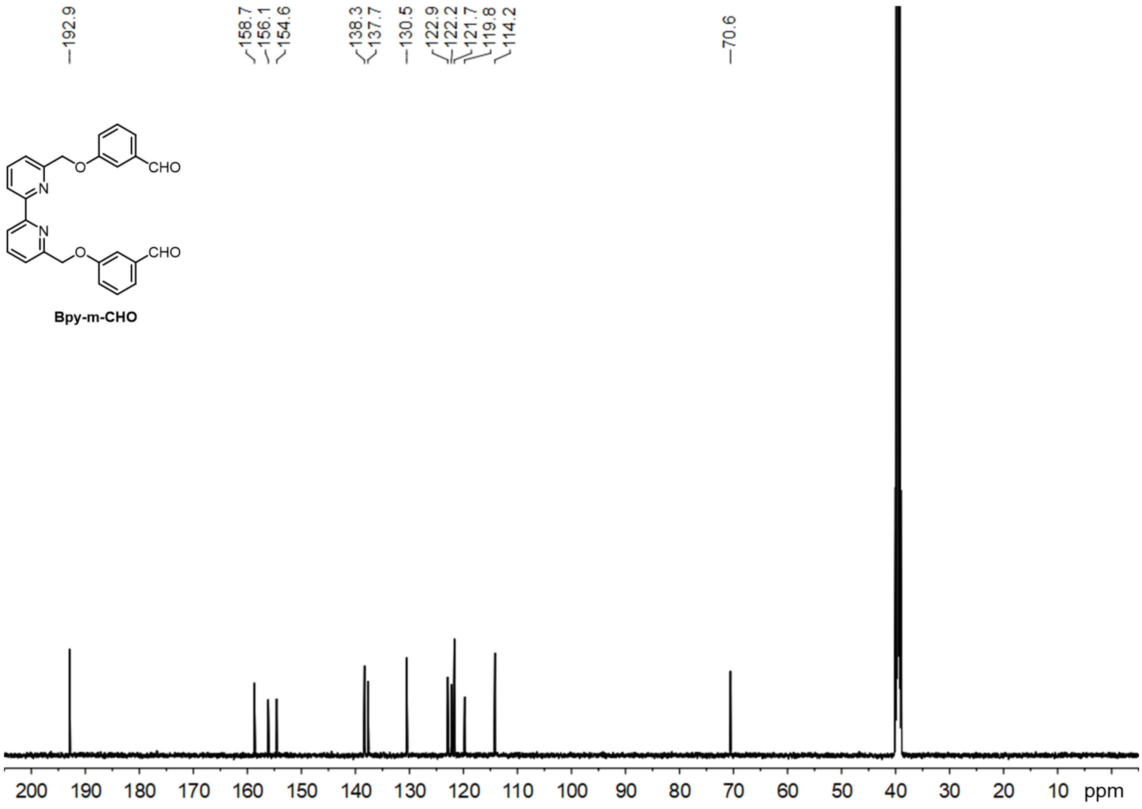

Supplementary Fig. 9. ^{13}C{^{1}H} NMR (125 MHz, DMSO-d_6, 298 K) of **Bpy-m-CHO**.

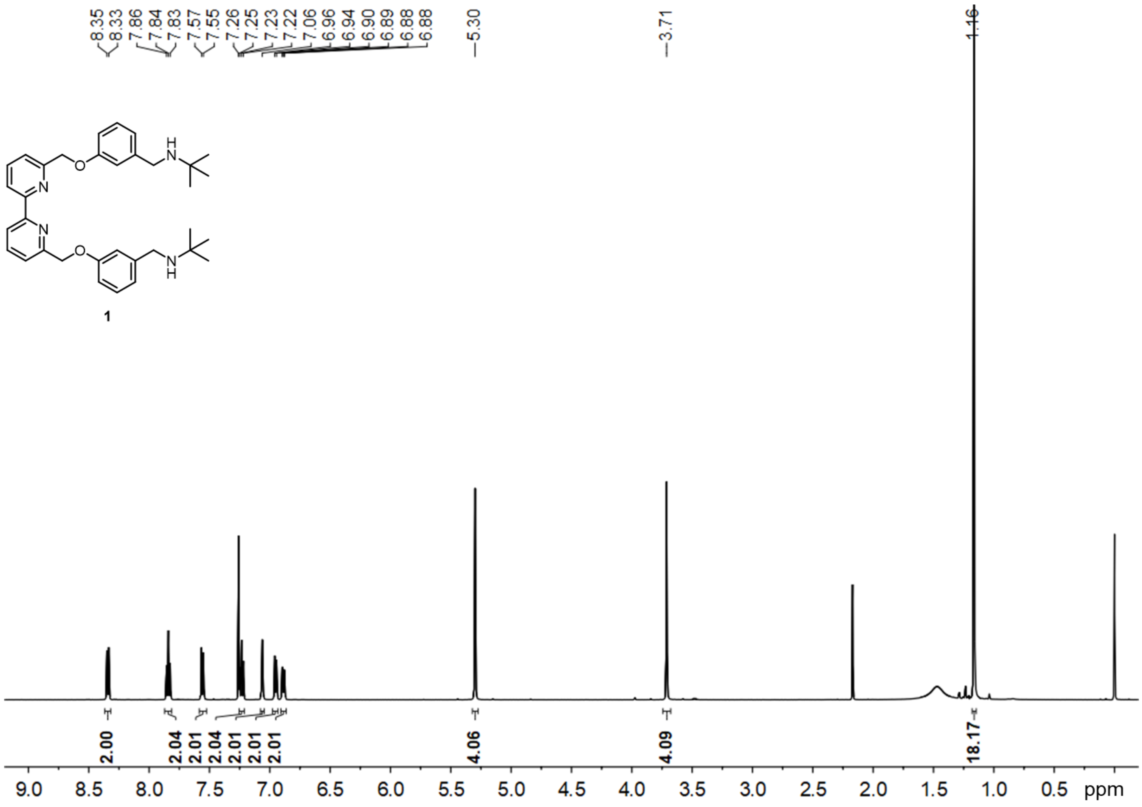

Supplementary Fig. 10. ^{1}H NMR (500 MHz, $CDCl_3$, 298 K) of **1**.

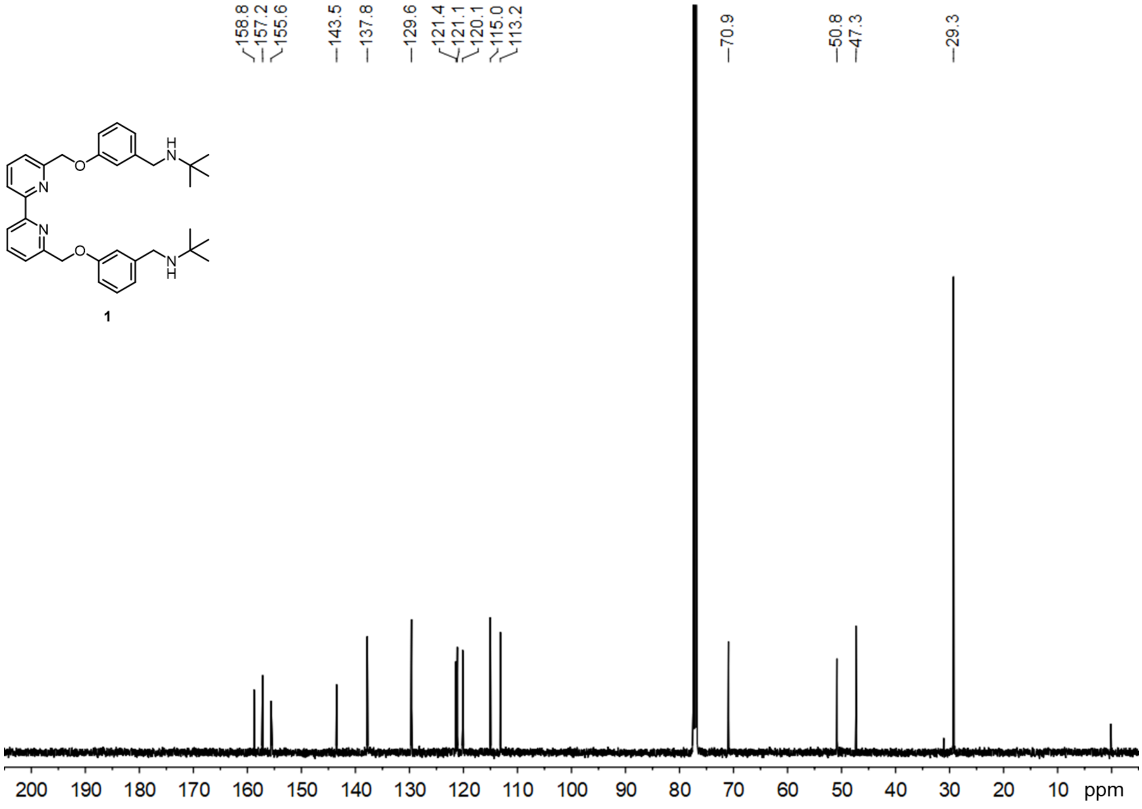

Supplementary Fig. 11. $^{13}C\{^{1}H\}$ NMR (125 MHz, $CDCl_3$, 298 K) of **1**.

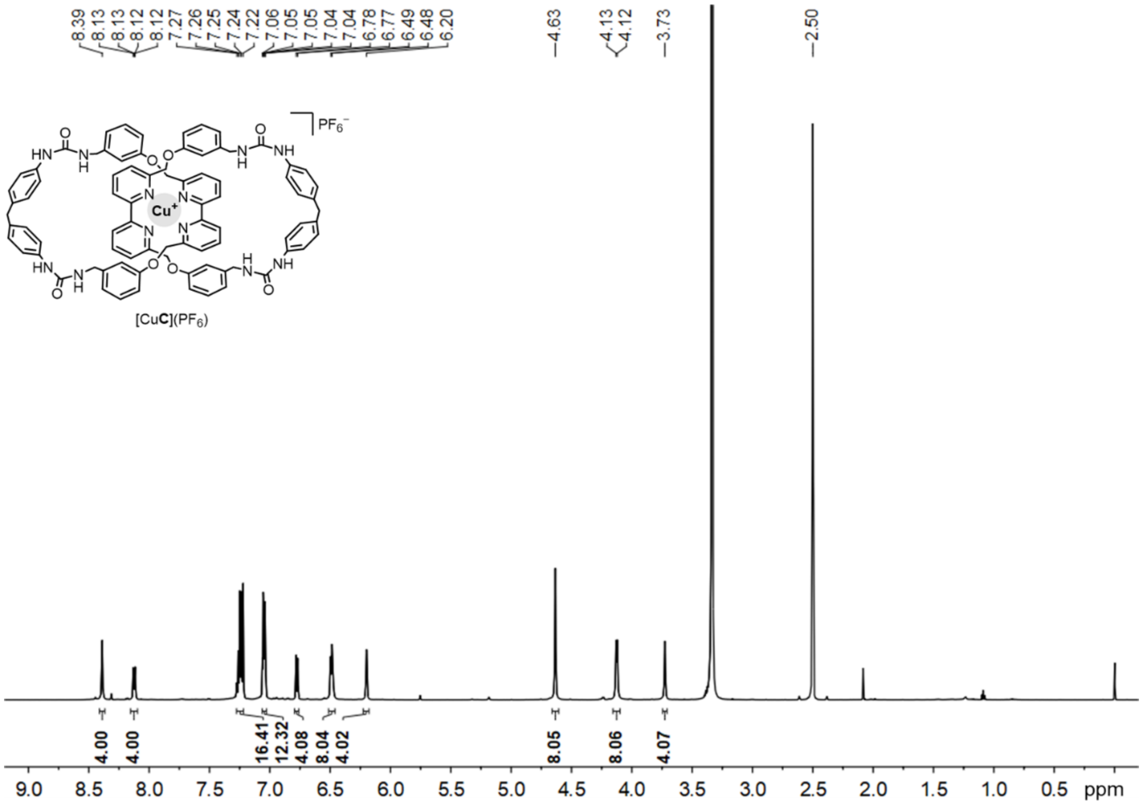

Supplementary Fig. 12. ^{1}H NMR (600 MHz, DMSO-d_6, 298 K) of [Cu**C**](PF_6).

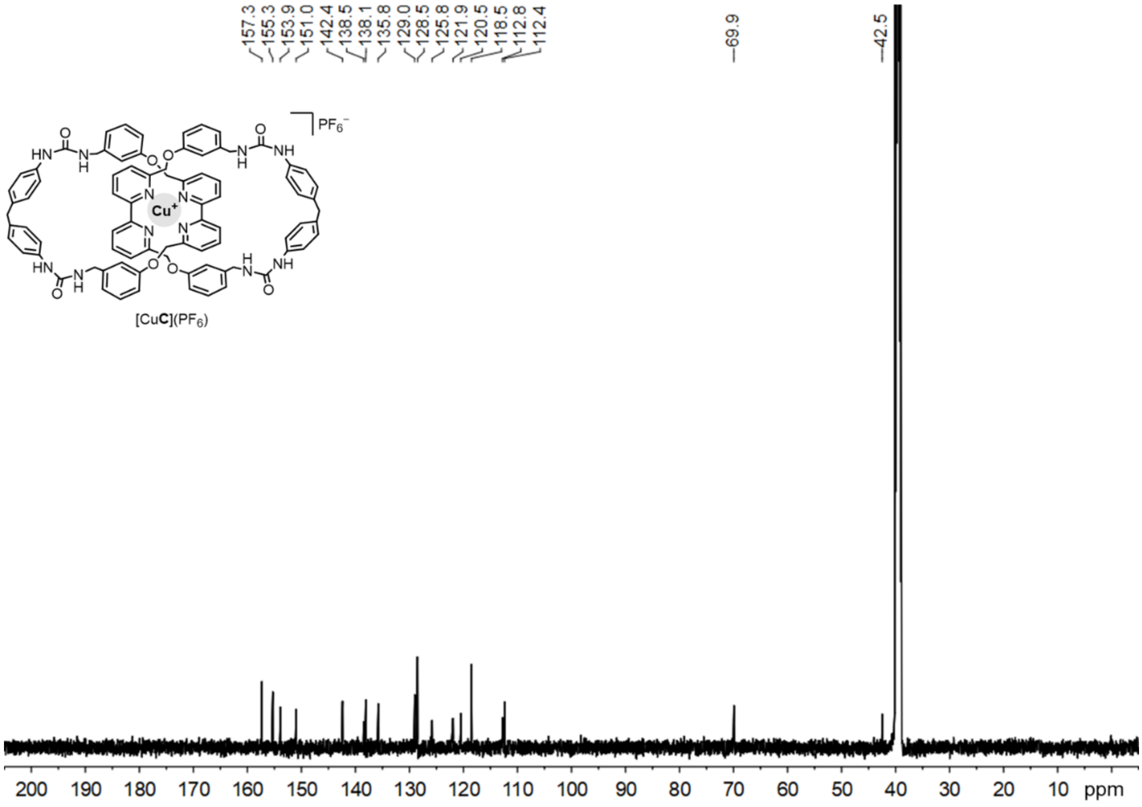

Supplementary Fig. 13. $^{13}C\{^{1}H\}$ NMR (125 MHz, DMSO-d_6, 298 K) of [Cu**C**](PF_6).

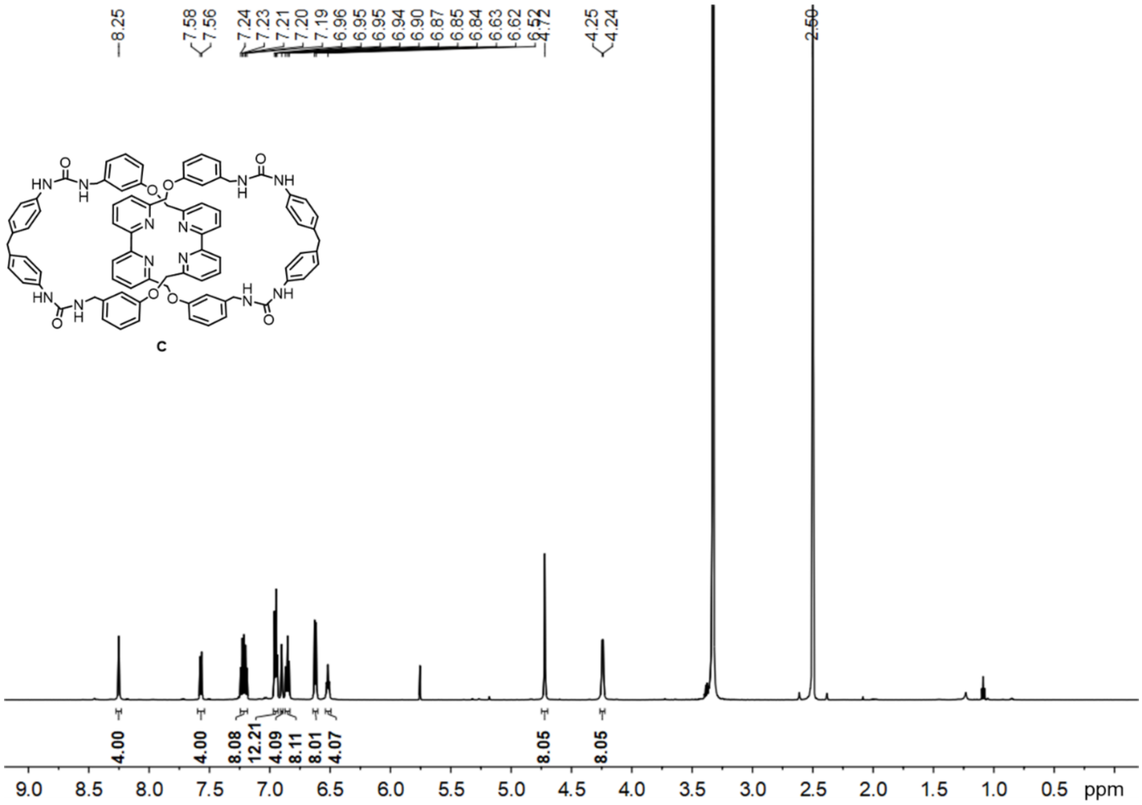

Supplementary Fig. 14. ^{1}H NMR (600 MHz, DMSO-d_6, 298 K) of **C**.

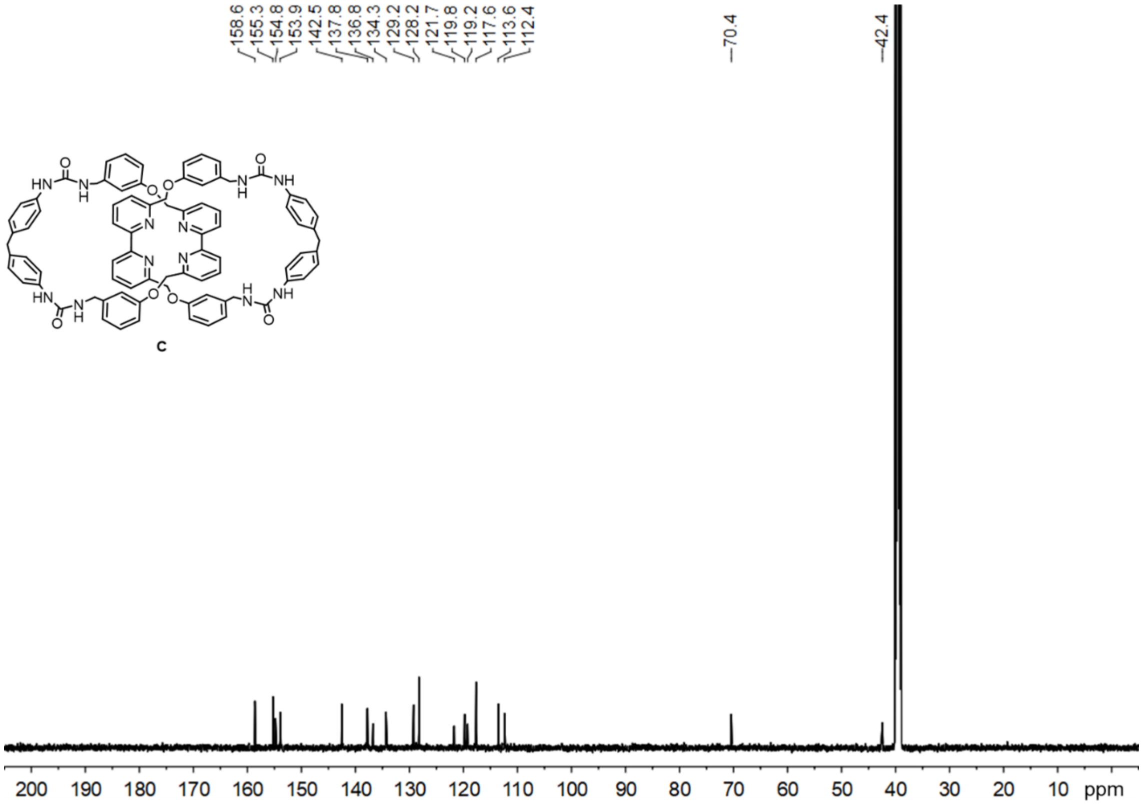

Supplementary Fig. 15. ^{13}C{^{1}H} NMR (125 MHz, DMSO-d_6, 298 K) of **C**.

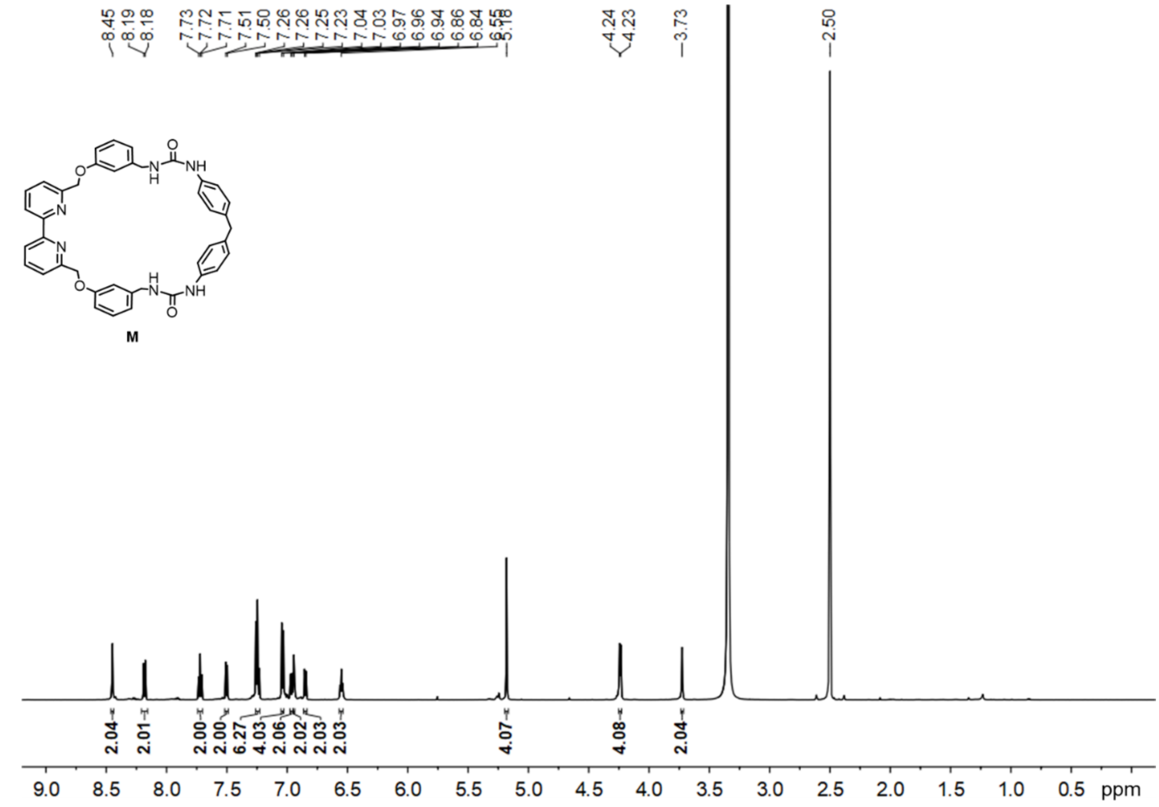

Supplementary Fig. 16. ^{1}H NMR (600 MHz, DMSO-d_6, 298 K) of **M**.

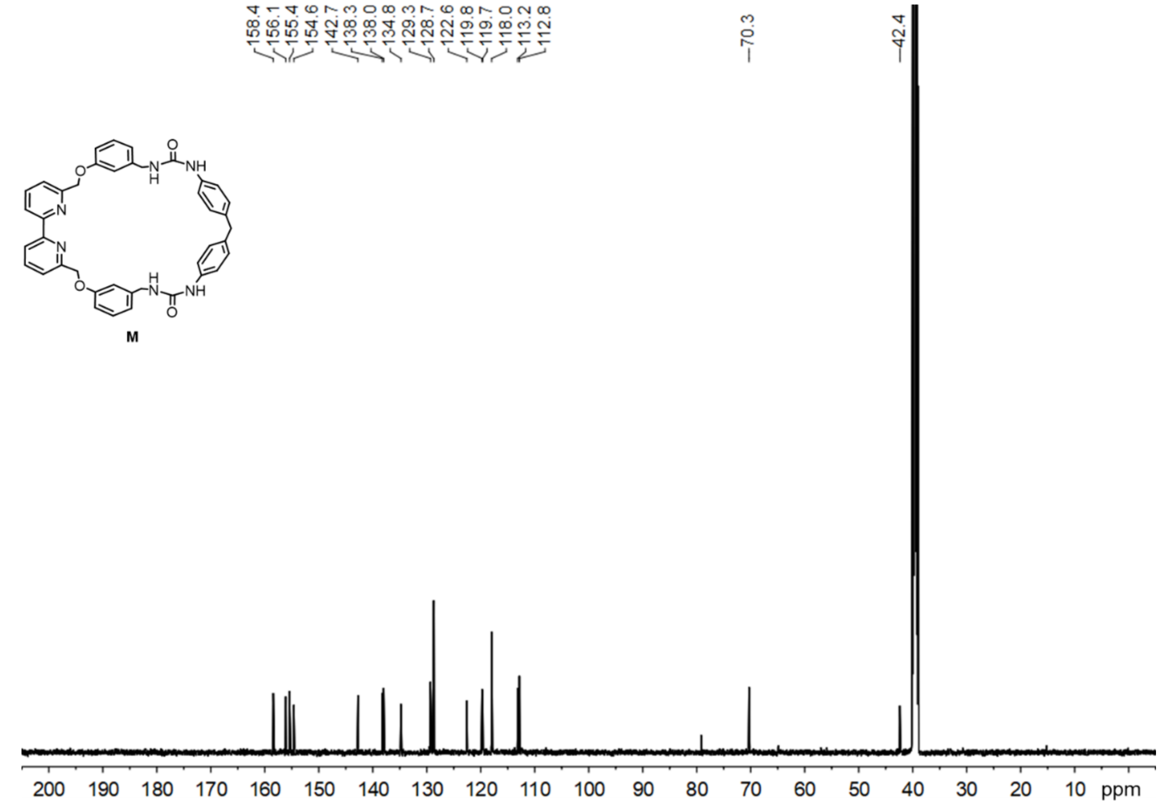

Supplementary Fig. 17. ^{13}C{^{1}H} NMR (125 MHz, DMSO-d_6, 298 K) of **M**.

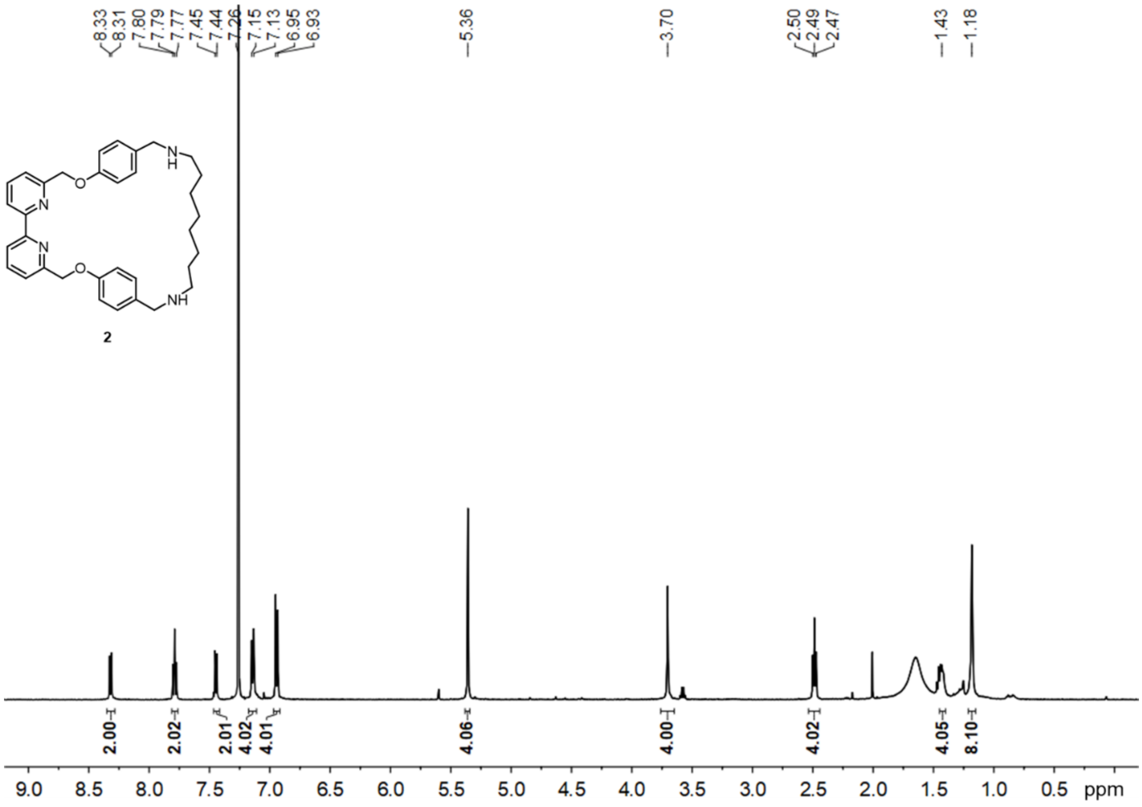

Supplementary Fig. 18. ^{1}H NMR (500 MHz, $CDCl_3$, 298 K) of **2**.

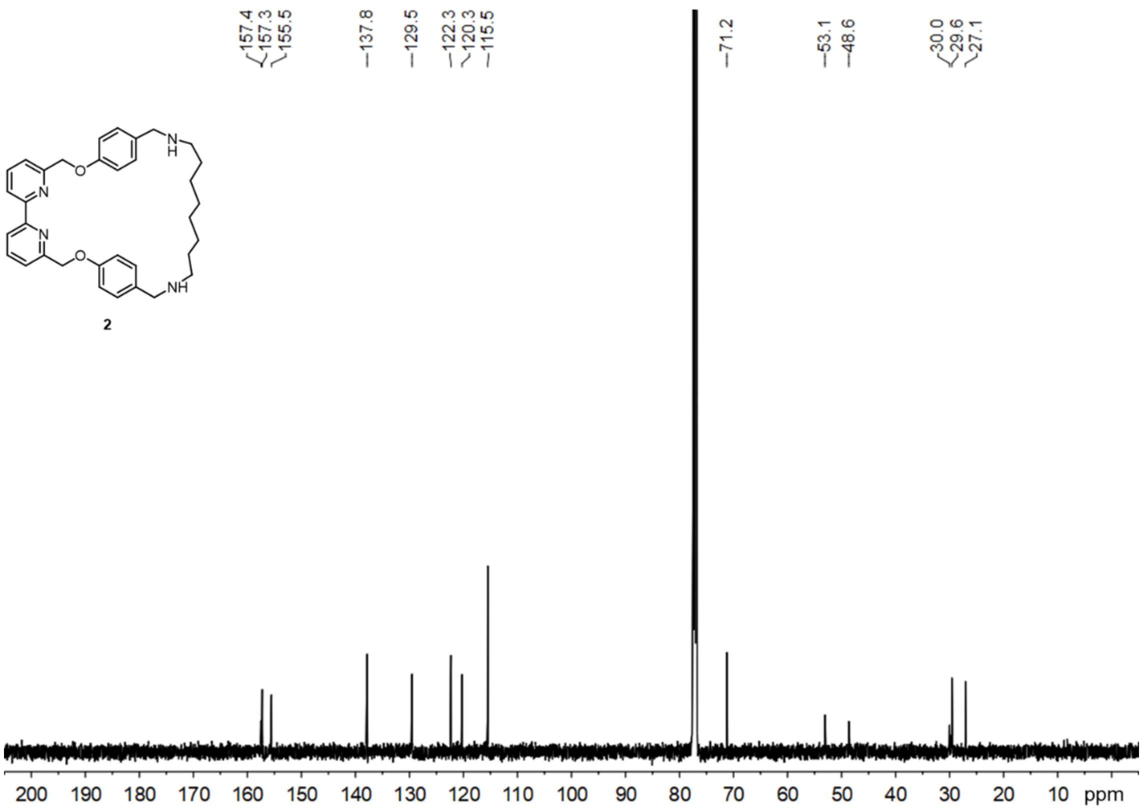

Supplementary Fig. 19. $^{13}C\{^{1}H\}$ NMR (125 MHz, $CDCl_3$, 298 K) of **2**.

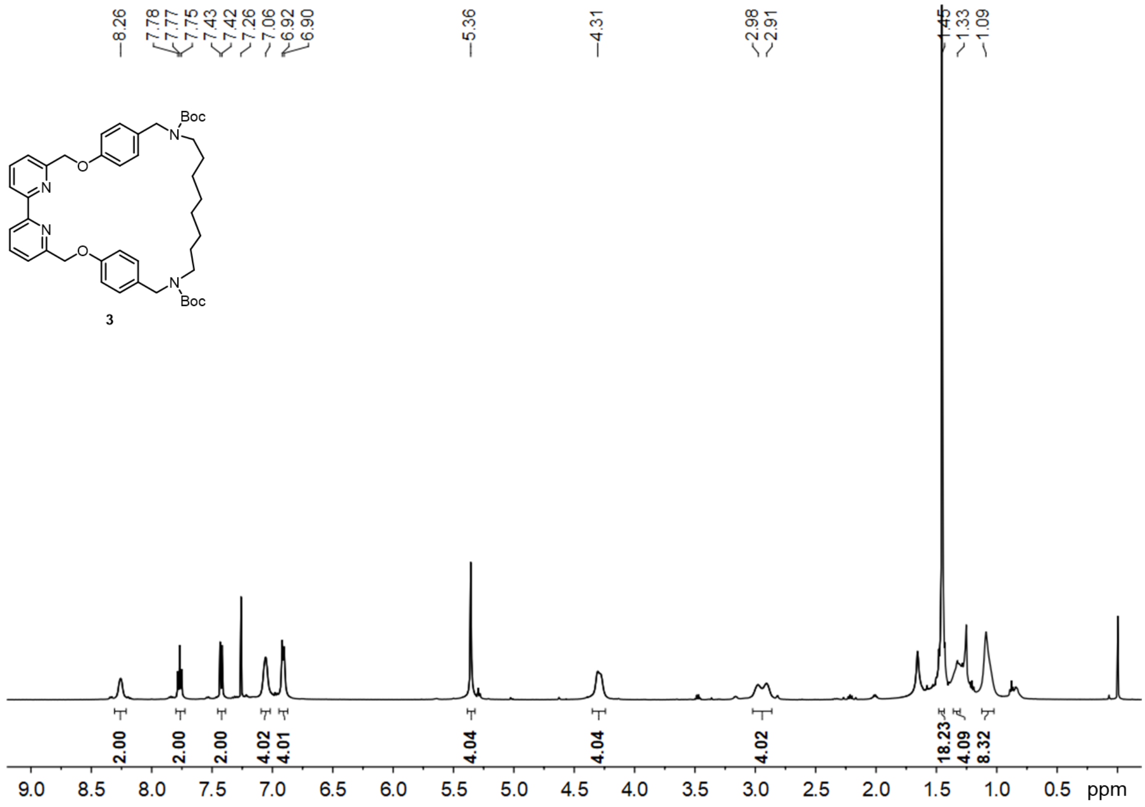

Supplementary Fig. 20. ^{1}H NMR (500 MHz, $CDCl_3$, 298 K) of **3**.

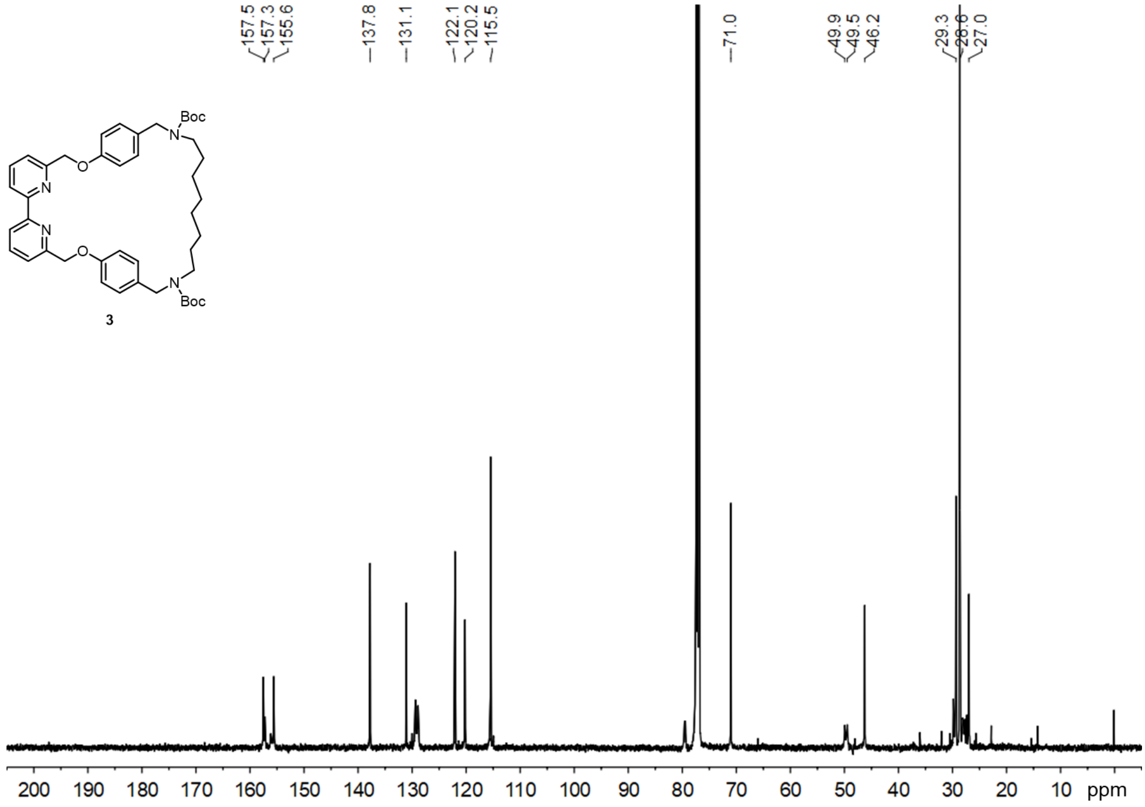

Supplementary Fig. 21. $^{13}C\{^{1}H\}$ NMR (125 MHz, $CDCl_3$, 298 K) of **3**.

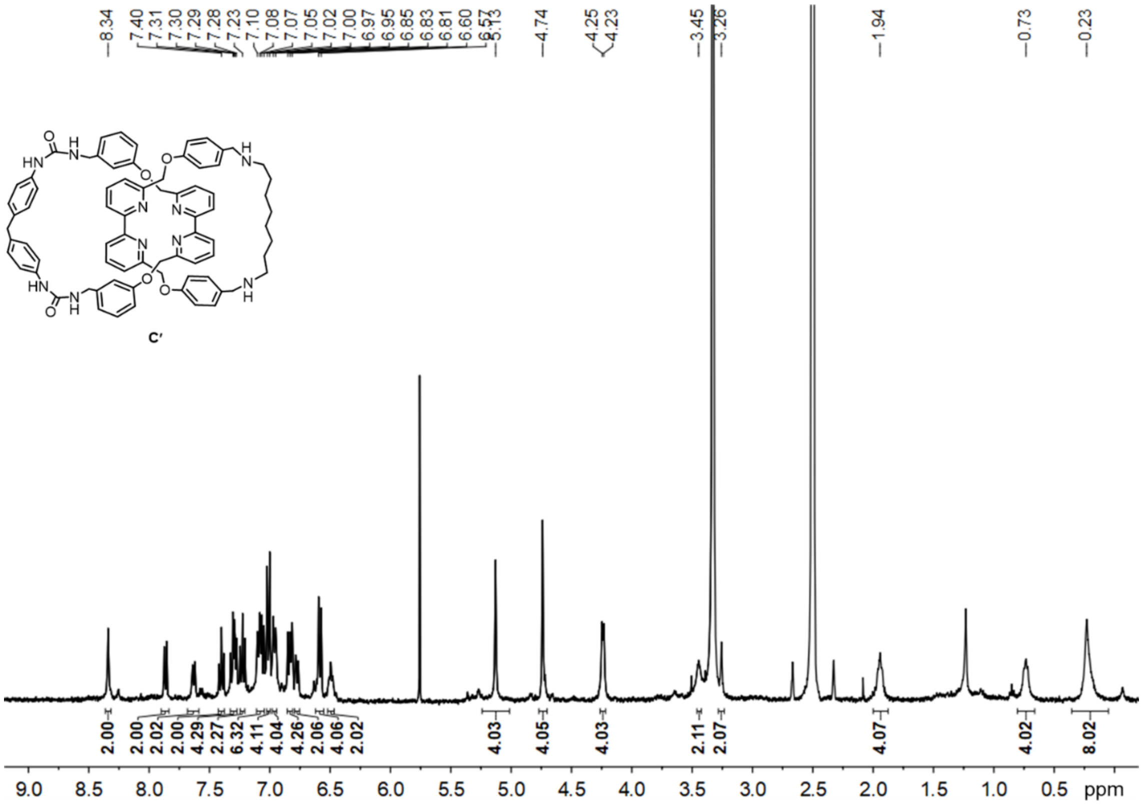

Supplementary Fig. 22. ^{1}H NMR (400 MHz, DMSO-d_6, 298 K) of **C′**.

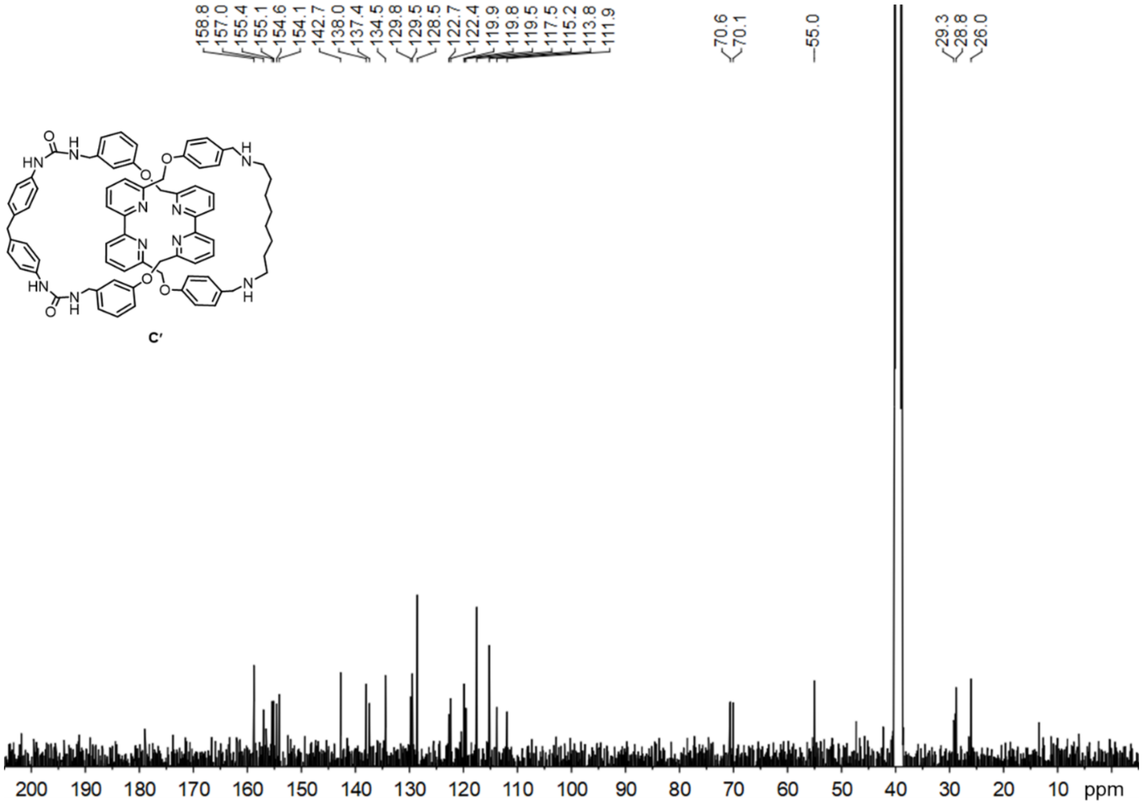

Supplementary Fig. 23. ^{13}C{^{1}H} NMR (100 MHz, DMSO-d_6, 298 K) of **C′**.

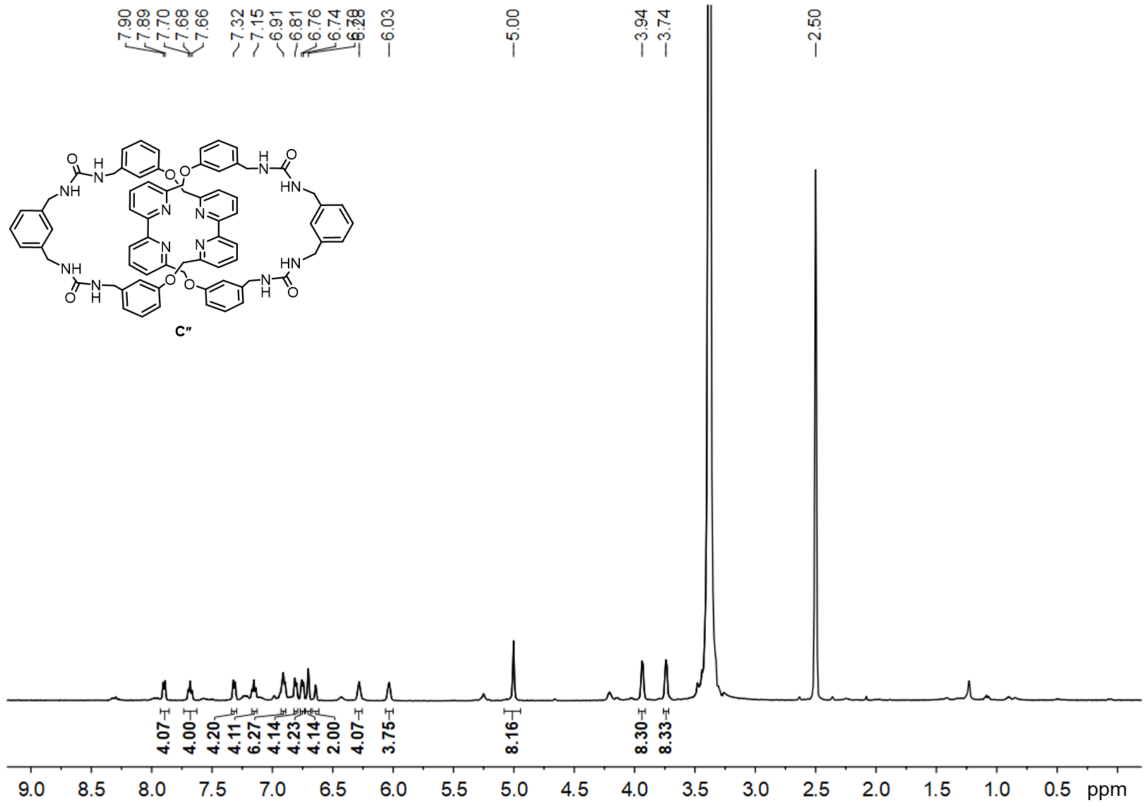

Supplementary Fig. 24. ^{1}H NMR (500 MHz, DMSO-d_6, 298 K) of **C″**.

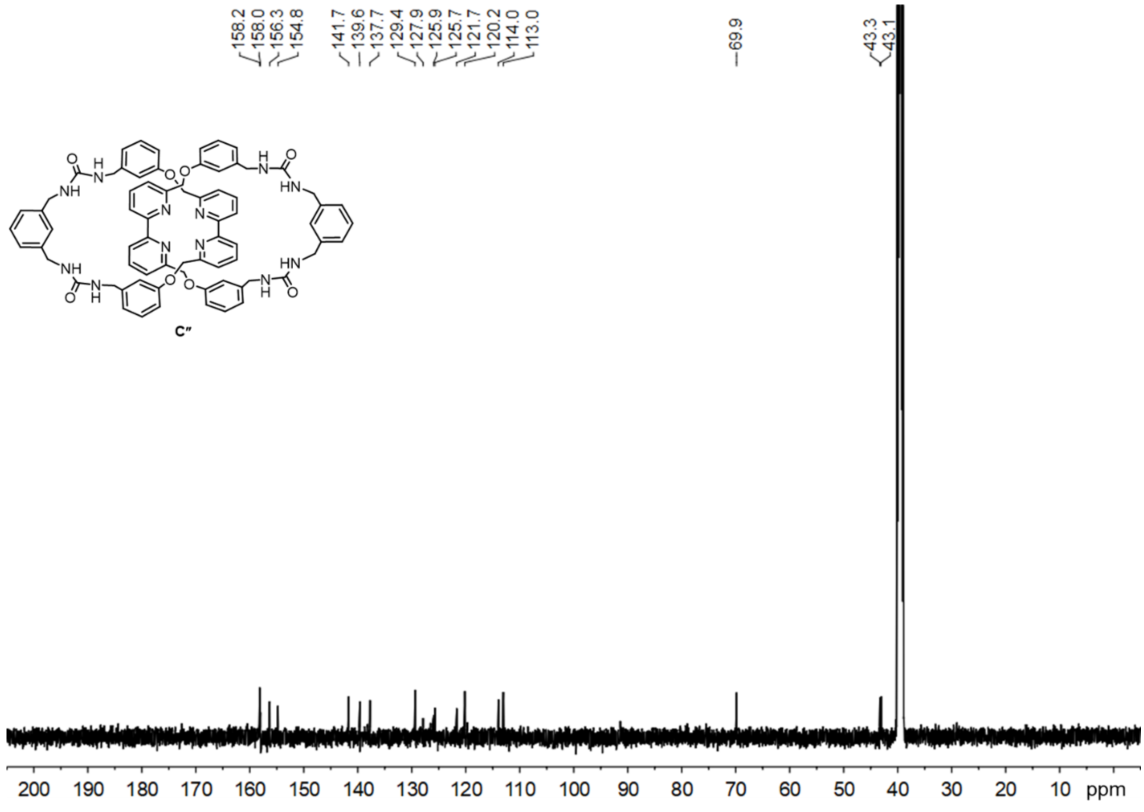

Supplementary Fig. 25. ^{13}C{^{1}H} NMR (125 MHz, DMSO-d_6, 298 K) of **C″**.

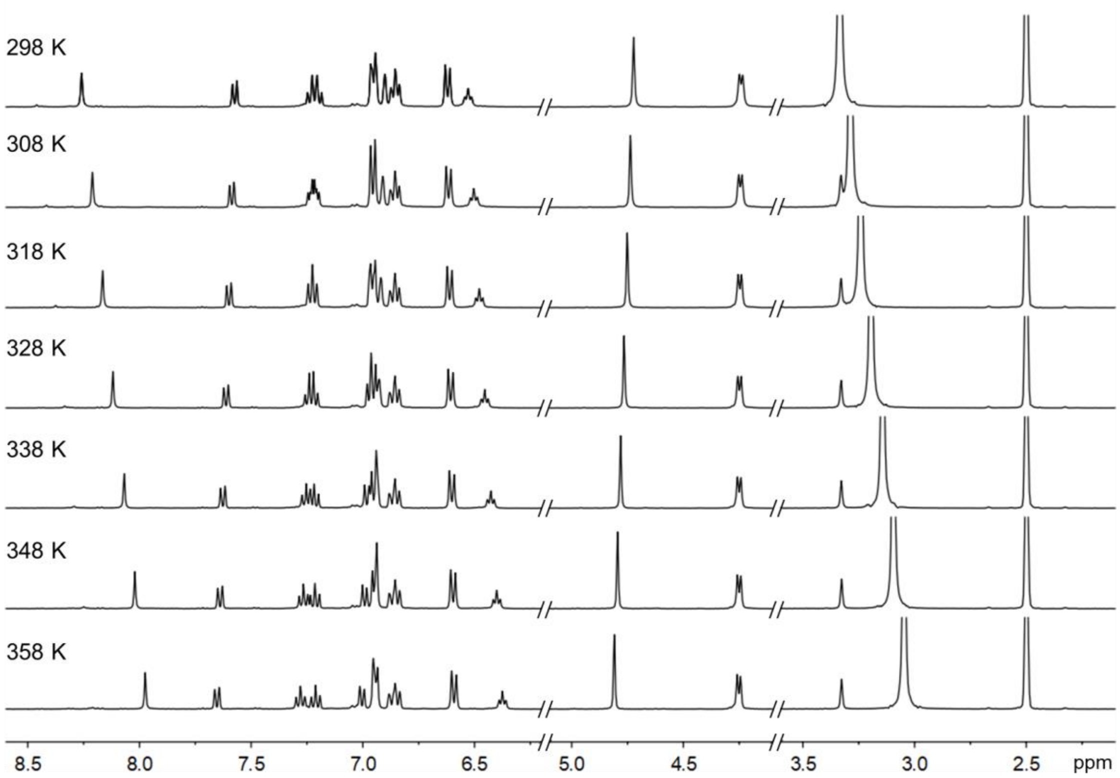

Supplementary Fig. 26. Partial ^{1}H NMR spectra (400 MHz, DMSO-d_6) of **C** obtained at temperature from 298 K to 358 K.

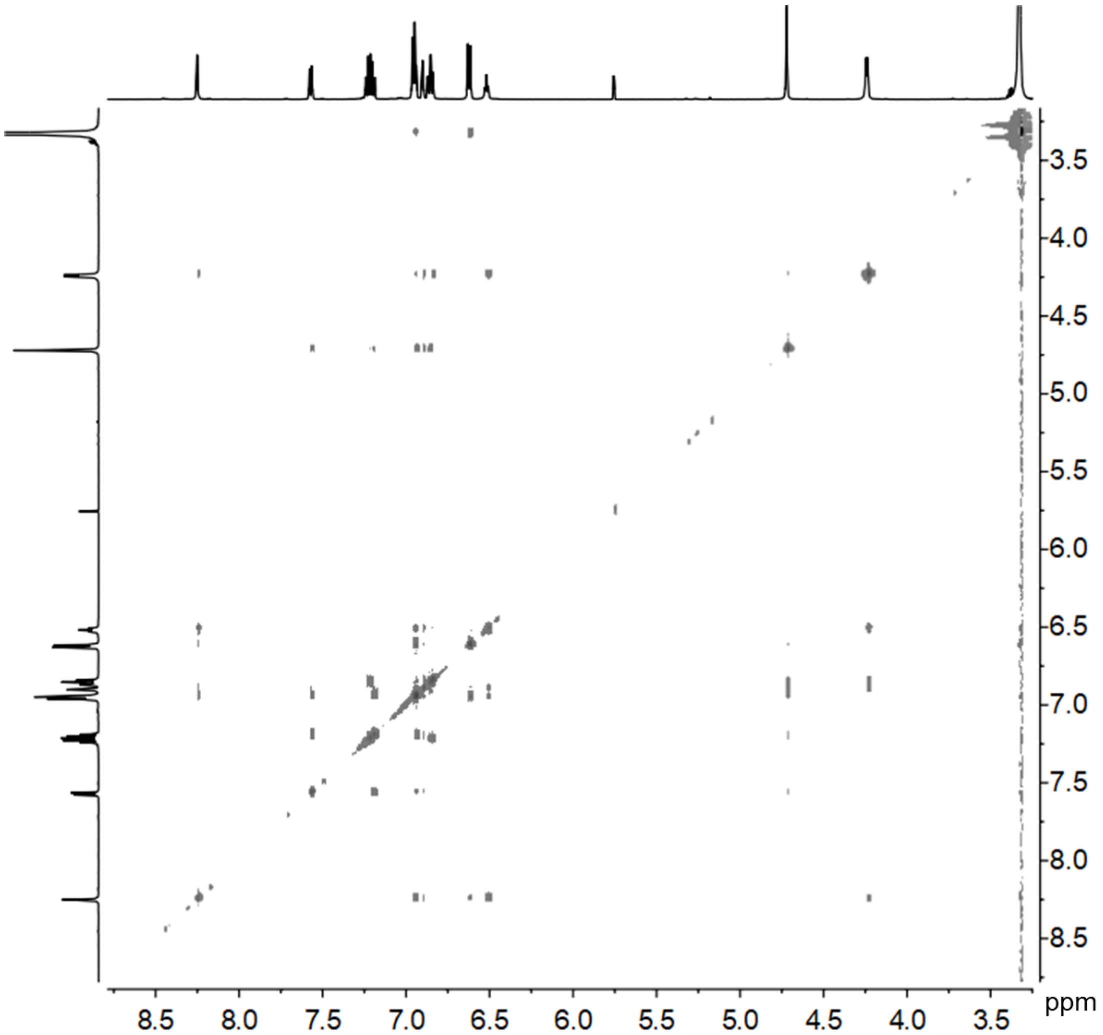

Supplementary Fig. 27. 2D NOESY NMR (600 MHz, DMSO-d_6, 298 K, mixing time = 900 ms) of **C**.

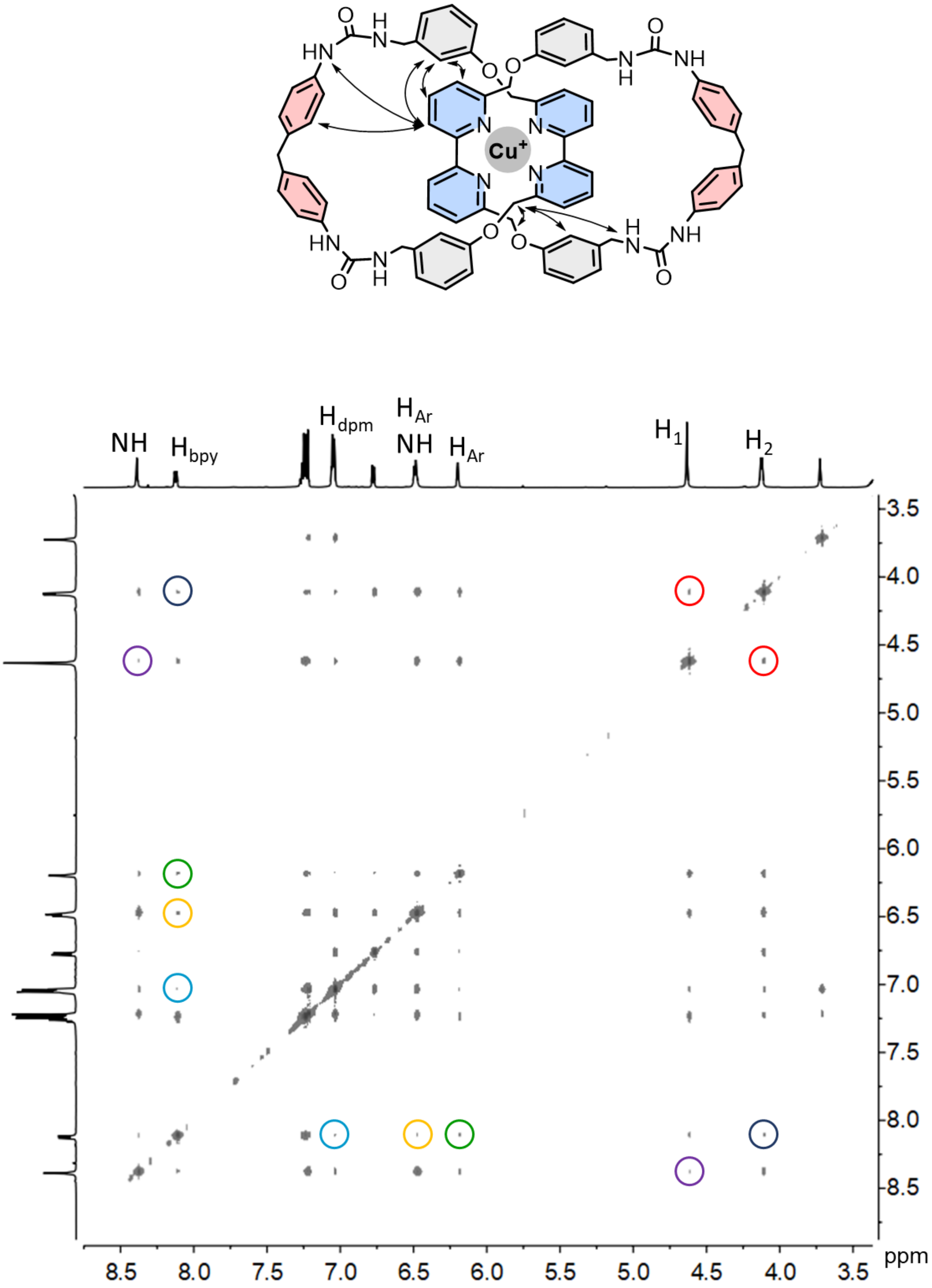

Supplementary Fig. 28. 2D NOESY NMR (600 MHz, DMSO-d_6, 298 K, mixing time = 900 ms) of [Cu**C**](PF_6). Key NOE cross peaks between macrocycles are highlighted.

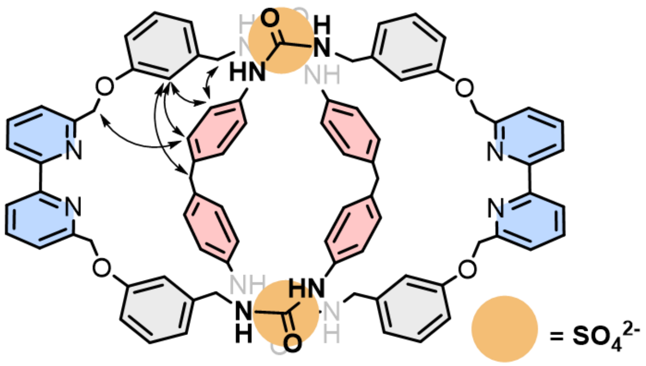

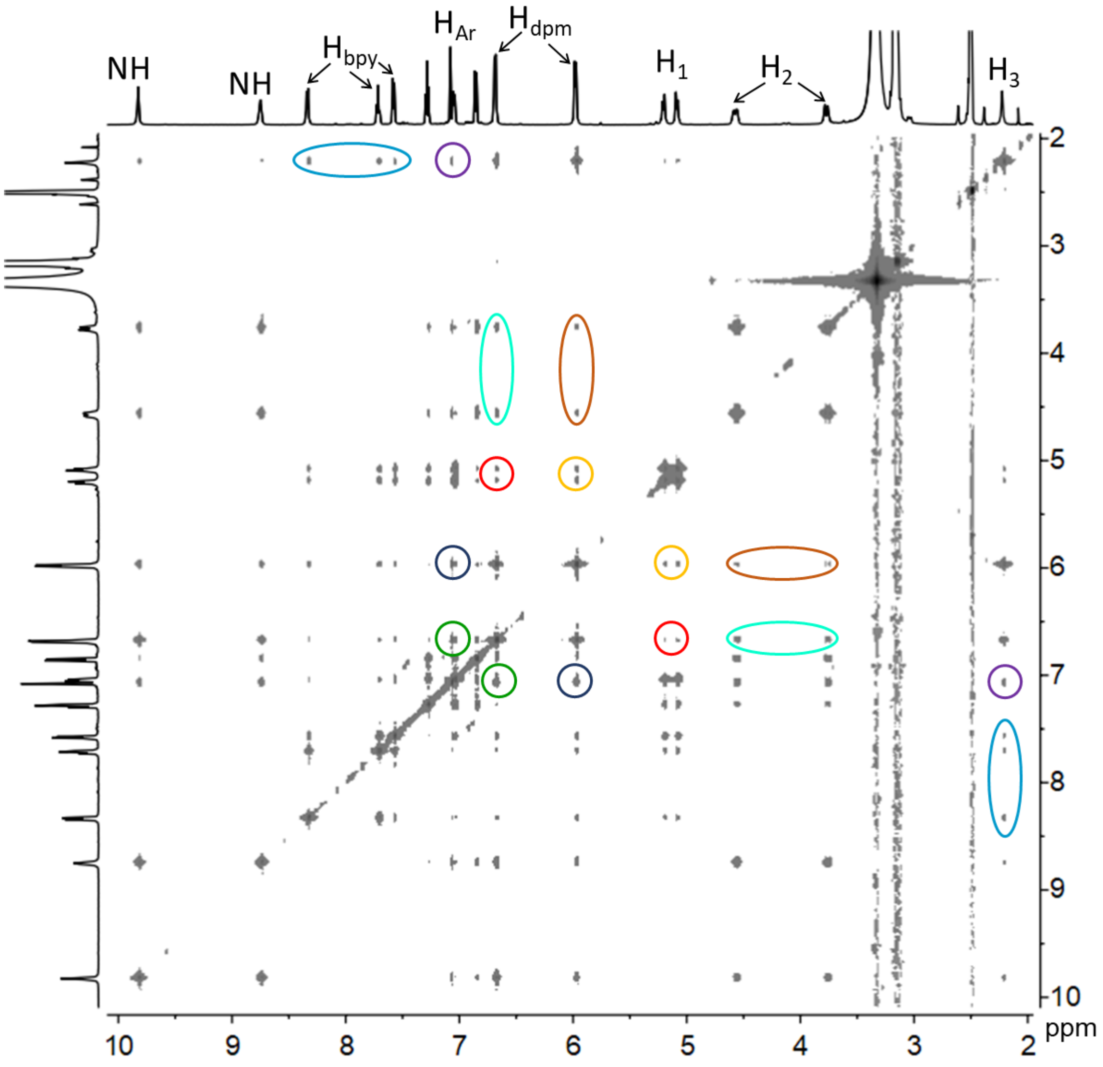

Supplementary Fig. 29. 2D NOESY NMR (600 MHz, DMSO-d_6, 298 K, mixing time = 900 ms) of **C** in the presence of 10 eq. of TBA_2SO_4. Key NOE cross peaks between macrocycles are highlighted.

3. Cation Binding Studies

Metal salts (including $[Cu(MeCN)_4](PF_6)$, $AgBF_4$, $LiBF_4$, $NaClO_4$, $Fe(ClO_4)_2$, $Co(ClO_4)_2$, $Ni(ClO_4)_2$, $Cu(ClO_4)_2$, $Zn(ClO_4)_2$ and $Cd(ClO_4)_2$) were purchased from commercial suppliers (J&K, Sigma-Aldrich, Aladdin and TCI, purity ≥ 98%) and stored in a vacuum desiccator containing silica gel before use. DMSO-d_6 for stock solutions and NMR samples was purchased from Energy and used without further purification.

Cation binding was studied by UV-Vis, NMR, and ESI-MS. For UV-Vis studies, an Agilent Cary 50 UV/Vis spectrophotometer was used. Absorbance was measured from 200 nm to 800 nm at a scan rate of 4800 nm/min and a 1-nm interval. Samples were contained in a quartz cuvette with a path length of 1 cm and 1.5 mL cell volume. Stock solutions of the tested cations (12.5 mM and 50 mM) were prepared using $Cu(MeCN)_4(PF_6)$, $AgBF_4$, $LiBF_4$, $NaClO_4$, $Fe(ClO_4)_2$, $Co(ClO_4)_2$, $Ni(ClO_4)_2$, $Cu(ClO_4)_2$, $Zn(ClO_4)_2$ and $Cd(ClO_4)_2$ in DMSO containing 125 μM of **C**. Aliquots of the cation stock solution were added to a solution of **C** (1000 μL, 125 μM in DMSO) in a quartz cuvette. The samples were mixed well at room temperature using a disposable pipette after each addition before an absorption spectrum was collected. For ^1H NMR binding studies, stock solutions of $Cu(MeCN)_4(PF_6)$, $AgBF_4$, $LiBF_4$, $NaClO_4$, $Fe(ClO_4)_2$, $Co(ClO_4)_2$, $Ni(ClO_4)_2$, $Cu(ClO_4)_2$, $Zn(ClO_4)_2$ and $Cd(ClO_4)_2$ were prepared in DMSO-d_6 at 50 mM. Samples were prepared by mixing 10 μL of a cation stock solution to a solution of **C** (500 μL, 1 mM) in DMSO-d_6 to give a host-guest ratio of 1:1. ^1H NMR spectra were obtained at 298 K immediately using a Bruker DPX spectrometer with working frequencies of 500 MHz. These tested NMR samples were then used for HR-ESI-MS analysis. Reduction of Cu^{2+} upon adding the divalent metal to a DMSO solution of the catenane was observed, resulting in the formation of the Cu(I)-catenane complex [Cu**C**]$^+$ as shown by ^1H NMR, UV-Vis and HR-MS.

Binding of Cu^+ or Ag^+ to **C** was further studied using ^1H NMR by sequential addition of aliquots of the $[Cu(MeCN)_4](PF_6)$ or $AgBF_4$ stock solution to a solution of **C** (500 μL, 1 mM in DMSO-d_6) to achieve the desired accumulated eq. of the guest. For ^1H NMR titration of Ag^+ to the catenane, a gradual shift of the proton resonances of the catenane was observed, suggesting the formation of a new species that is fast exchanging with **C**. Subsequent addition of stoichiometric amount of Cu^+ to the Ag^+-containing titration

mixture resulted in complete conversion to $[Cu\mathbf{C}]^+$, showing that the Ag^+ binding to **C** is much weaker, and the binding is selective to Cu^+ over Ag^+.

Considering that related phenanthroline-based [2]catenanes have also been shown to bind to the d^{10} Zn(II) ion, Zn^{2+} binding towards the bis-bipyridyl unit was further studied by ^{1}H NMR using 6,6'-dimethyl-2,2'-bipyridine (**DMbpy**, purchased from Aladdin, purity ≥ 98%) as a model ligand. Coordination of **DMbpy** to Zn^{2+} was studied in DMSO-d_6 and CD_3CN using stock solutions of **DMbpy** at 2 mM and $Zn(ClO_4)_2$ at 50 mM. Aliquots of the Zn(II) stock solutions were titrated to the corresponding **DMbpy** solutions. While no obvious spectral change was observed when the titration was conducted in DMSO-d_6, a new set of bpy signals in slow exchange with those of the free bpy ligand was observed when the titration was conducted in CD_3CN, suggesting that the Zn(II)-bpy coordination is likely kinetically controlled and is very slow in DMSO-d_6.

Cu(I) binding of **C** was also studied by fluorescence spectroscopy. Spectra were recorded on an Edinburgh Instruments FS5 spectrophotometer equipped with a 150 W CW ozone-free xenon arc lamp and a photomultiplier R928P detection unit with spectral coverage of 200–870 nm. Samples for emission measurement were contained in a quartz cuvette with a path length of 1 cm and 1.5 mL cell volume. A stock solution of $Cu(MeCN)_4(PF_6)$ (12.5 mM) was prepared in DMSO containing 125 μM of **C**. Aliquots of Cu(I) stock solution was added to a solution of **C** (1000 μL, 125 μM in DMSO) in the quartz cuvette. The samples were mixed well at room temperature using a disposable pipette after each addition before an emission spectrum was collected. The samples were excited at 270 nm and emission spectra were collected from 290 nm to 700 nm. Two emission peaks at 350 nm and 460 nm were found in the fluorescence spectrum of **C**. Addition of Cu^+ or SO_4^{2-} ions both resulted in a decrease in the normalized emission intensity at 460 nm.

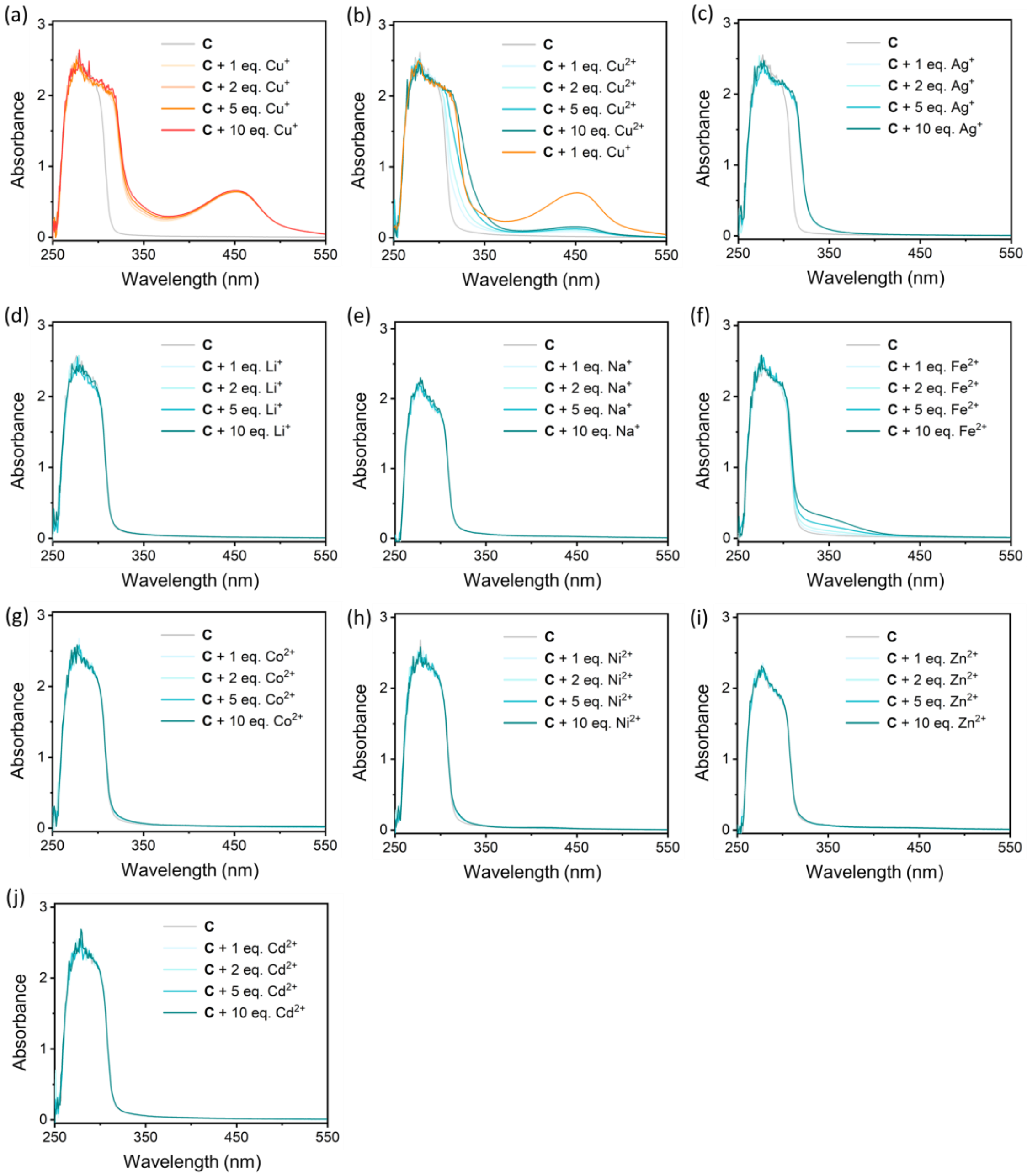

Supplementary Fig. 30. UV-Vis spectra of 125 μM solutions of **C** in DMSO at 298 K in the presence of 0 to 10 eq. of (a) Cu^{+}, (b) Cu^{2+} (compared with that in the presence of 1 eq. of Cu^{+}), (c) Ag^{+}, (d) Li^{+}, (e) Na^{+}, (f) Fe^{2+}, (g) Co^{2+}, (h) Ni^{2+}, (i) Zn^{2+} and (j) Cd^{2+}.

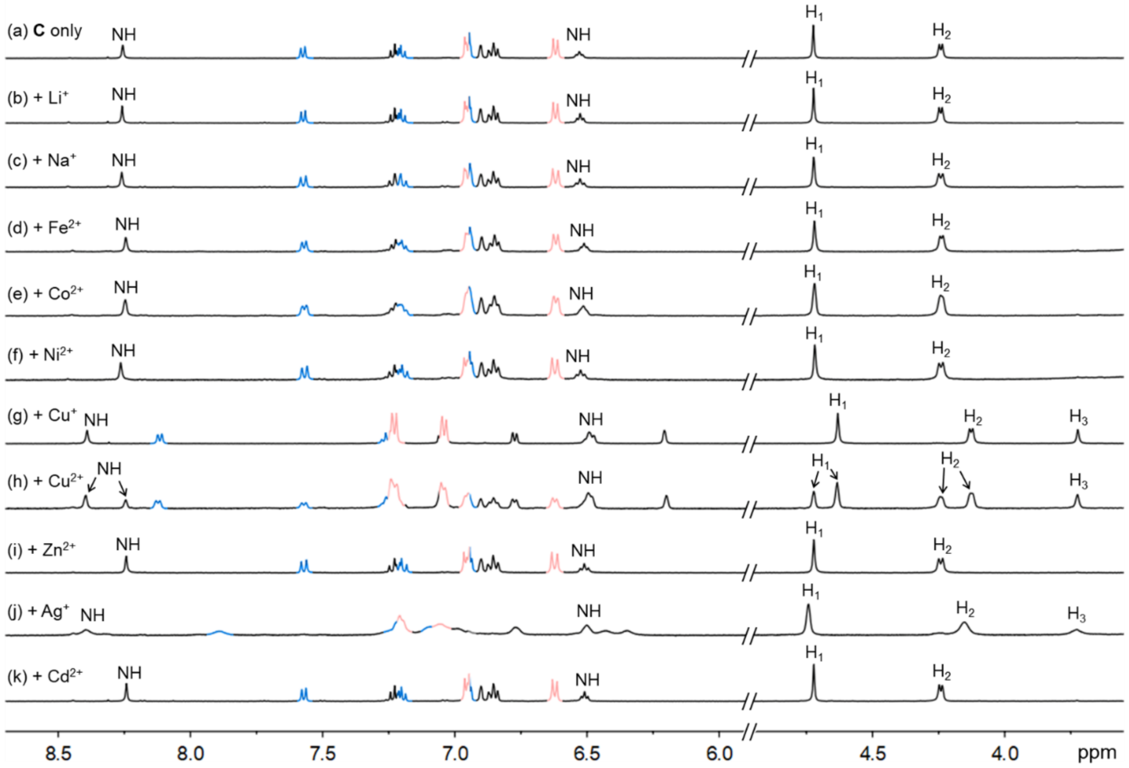

Supplementary Fig. 31. Partial ^{1}H NMR spectra (500 MHz, 298 K, DMSO-d_6) of a 1 mM solution of **C** in the presence of 1 eq. of different cations. Signals from H_{bpy} and H_{dpm} are highlighted in blue and pink respectively.

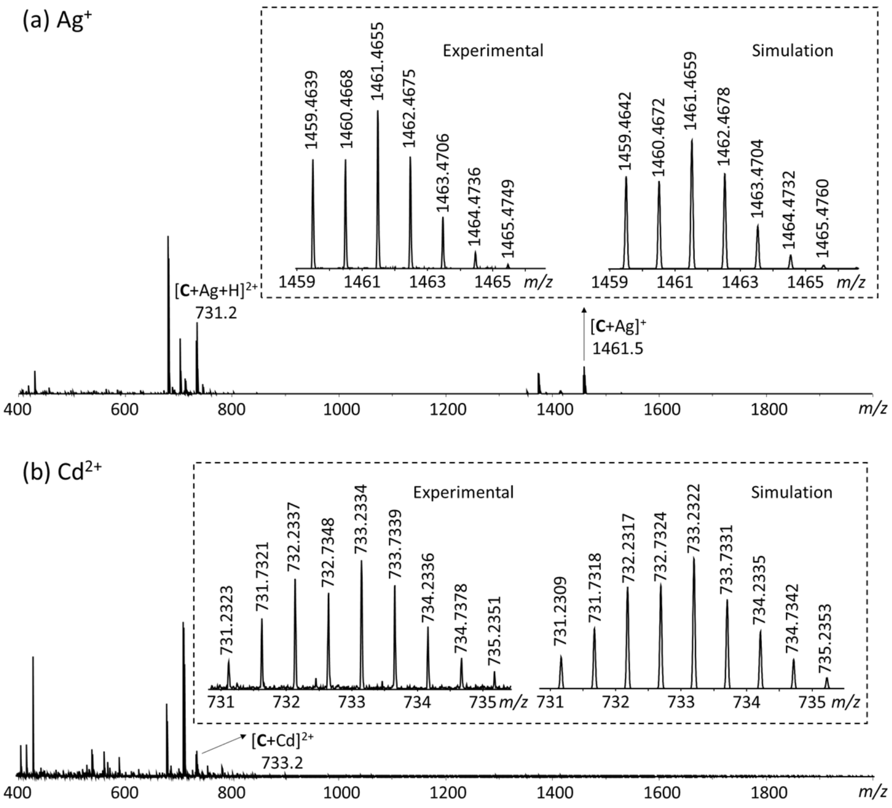

Supplementary Fig. 32. ESI-MS (+ve) spectra of (a) **C** with 1 eq. of Ag^+ (inset: isotopic distribution of the peak at *m/z* = 1461.5) and (b) **C** with 1 eq. of Cd^{2+} (inset: isotopic distribution of the peak at *m/z* = 733.2).

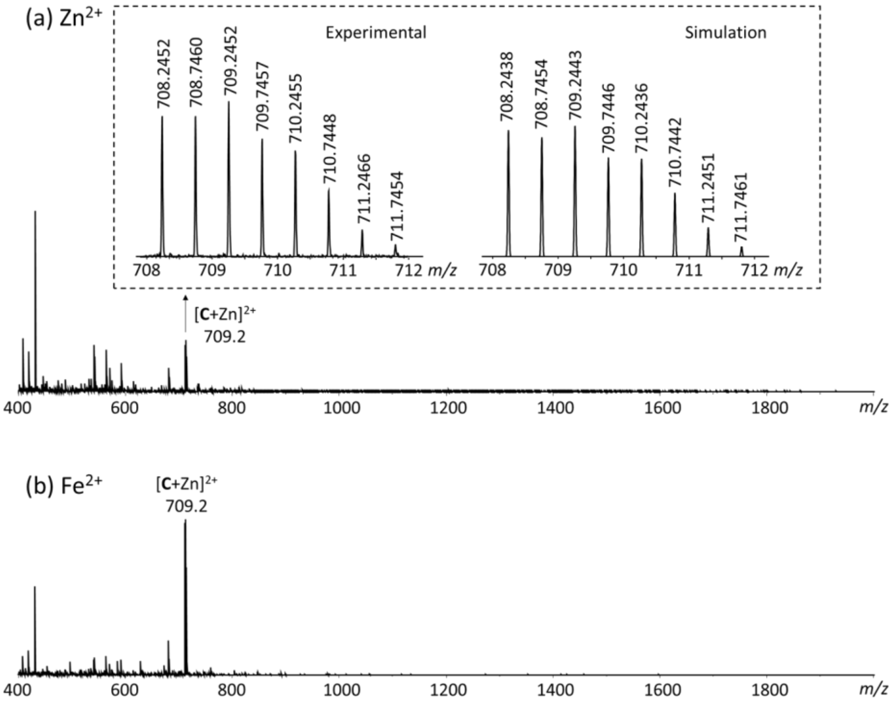

Supplementary Fig. 33. ESI-MS (+ve) spectra of (a) **C** with 1 eq. of Zn^{2+} (inset: isotopic distribution of the peak at *m/z* = 709.2) and (b) **C** with 1 eq. of Fe^{2+}.

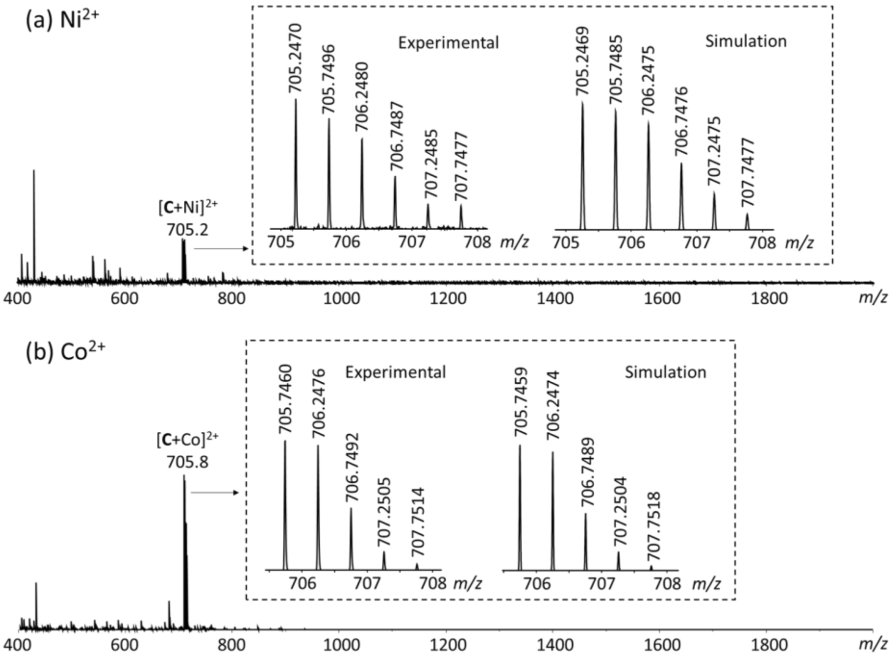

Supplementary Fig. 34. ESI-MS (+ve) spectra of (a) **C** with 1 eq. of Ni^{2+} (inset: isotopic distribution of the peak at *m/z* = 705.2) and (b) **C** with 1 eq. of Co^{2+} (inset: isotopic distribution of the peak at *m/z* = 705.8).

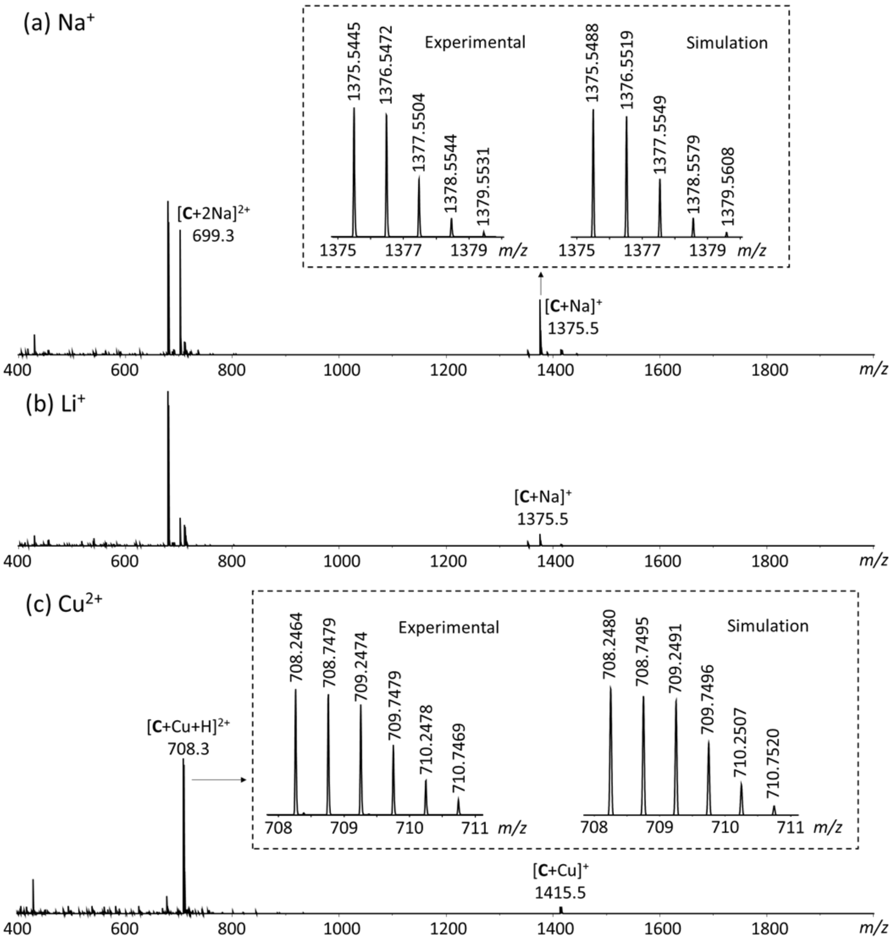

Supplementary Fig. 35. ESI-MS (+ve) spectra of (a) **C** with 1 eq. of Na^{+} (inset: isotopic distribution of the peak at *m/z* = 1375.5), (b) **C** with 1 eq. of Li^{+} and (c) **C** with 1 eq. of Cu^{2+} (inset: isotopic distribution of the peak at *m/z* = 708.3).

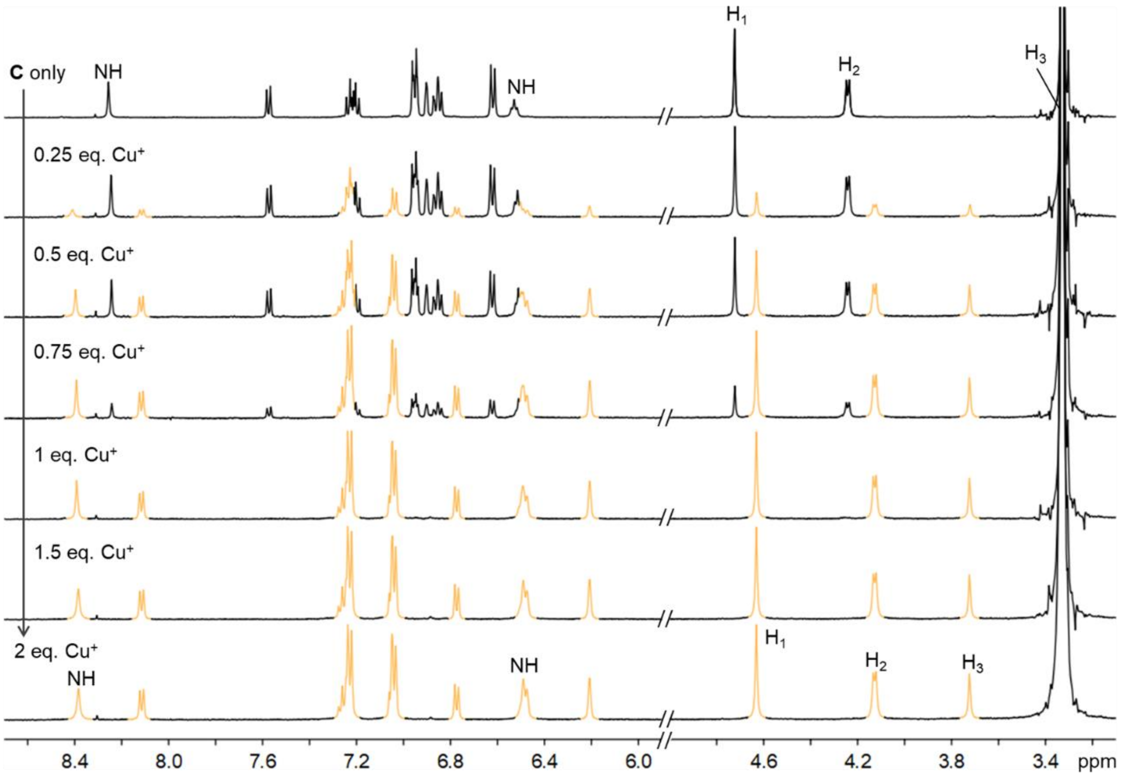

Supplementary Fig. 36. Partial ^{1}H NMR spectra (500 MHz, 298 K, DMSO-d_6) of a 1 mM solution of **C** in the presence of increasing amount of $[Cu(MeCN)_4](PF_6)$. Signals from [Cu**C**](PF_6) are highlighted in orange.

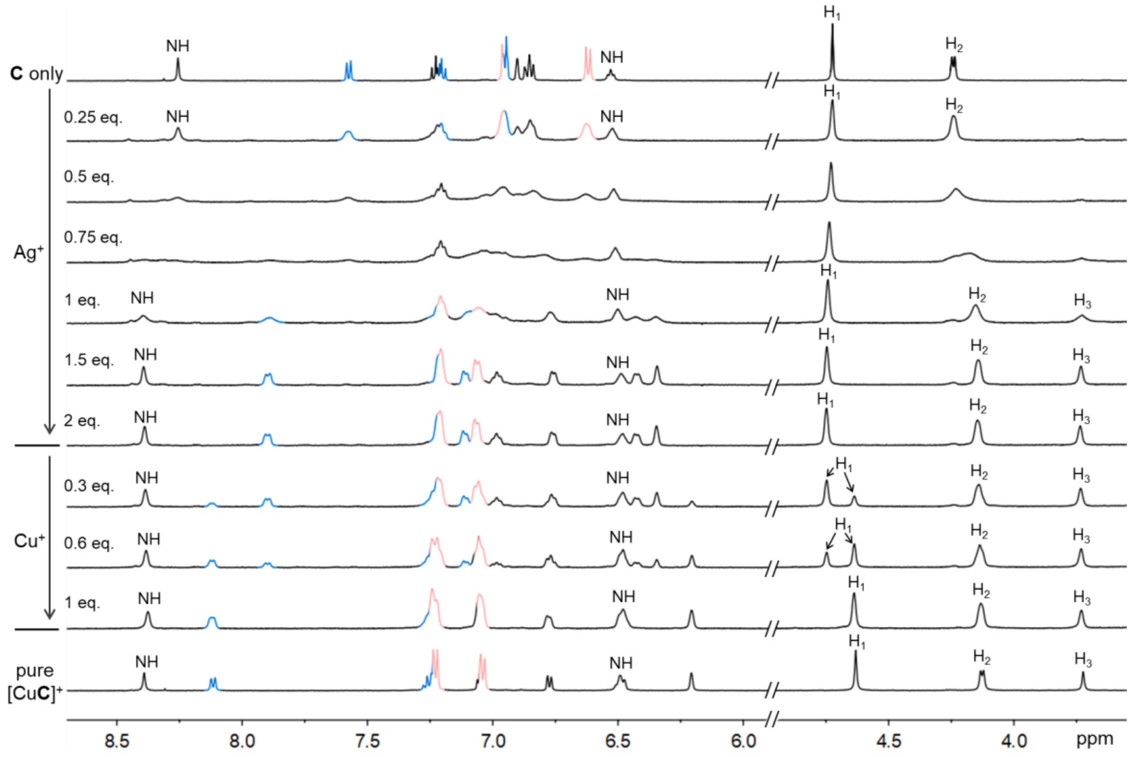

Supplementary Fig. 37. Partial ^{1}H NMR spectra (500 MHz, 298 K, DMSO-d_6) of a 1 mM solution of **C** in the presence of 0.25 eq. to 2 eq. of Ag^+, followed by sequential addition of 0.3 eq. to 1 eq. Cu^+ to displace the complexed Ag^+. Signals from H_{bpy} and H_{dpm} are highlighted in blue and pink respectively.

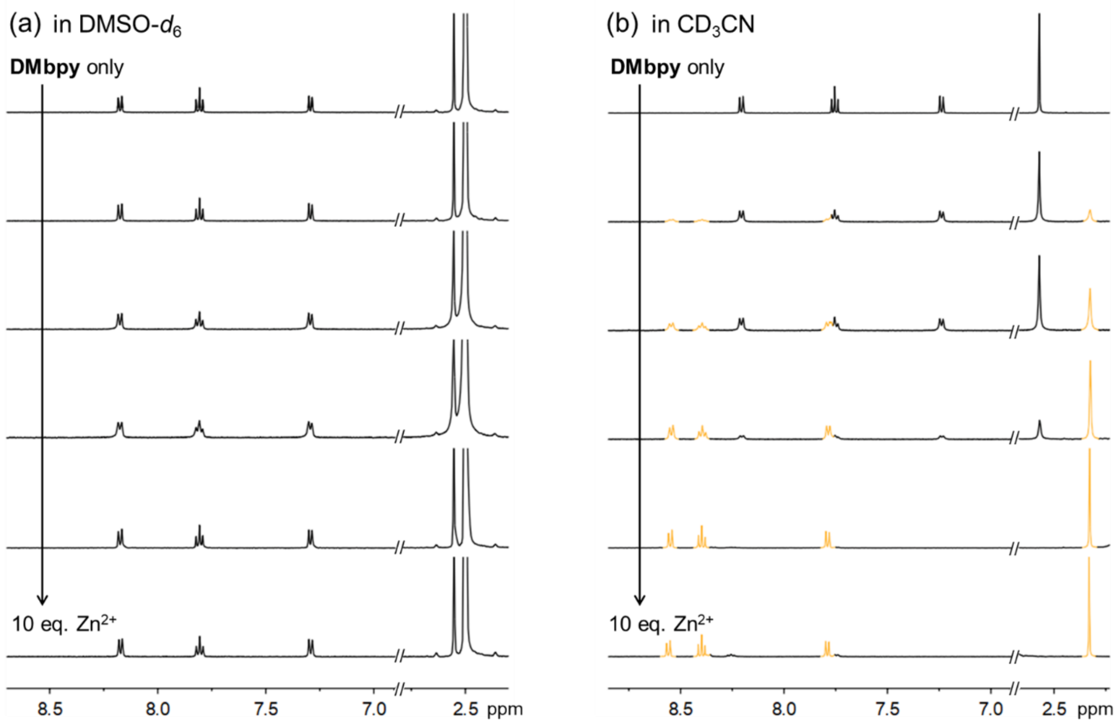

Supplementary Fig. 38. Partial ^{1}H NMR spectra (500 MHz, 298 K) of **DMbpy** in the presence of 0 eq. (top), 0.2 eq., 0.3 eq., 0.5 eq., 1 eq. and 10 eq. (bottom) of Zn^{2+} in (a) DMSO-d_6 and (b) CD_3CN, respectively. Signals from the Zn^{2+}-complexed **DMbpy** are highlighted in orange.

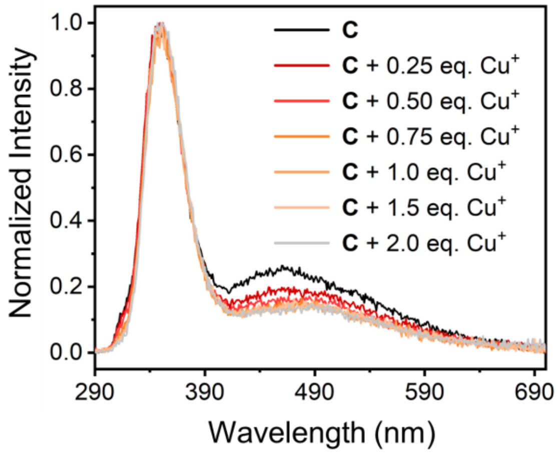

Supplementary Fig. 39. Normalized fluorescence spectra of the 125 μM solution of **C** in DMSO at 298 K in the presence of 0 to 2.0 eq. of Cu^+.

4. Anion Binding Studies

Tetrabutylammonium (TBA) salts were purchased from commercial suppliers (Aladdin, Energy and Dkmchem, purity ≥ 98% unless otherwise noted), dried *in vacuo* and stored in a vacuum desiccator containing silica gel prior to use. DMSO-d_6 for stock solutions and NMR samples was purchased from Energy and used without further purification unless otherwise noted. Stock solutions of TBA salts of chloride, bromide, iodide, nitrate, acetate, hydrogen sulfate, dihydrogen phosphate and sulfate were prepared in DMSO-d_6 at 50 mM. Concentrations of the stock solutions were calibrated by ^{1}H NMR using 1,3,5-trimethoxybenzene (purchased from Aladdin, purity ≥ 98%) as the internal standard.

Anion binding capability of **C** was first evaluated by ^{1}H NMR. Samples were prepared by mixing 100 μL of an anion stock solution to the solution of **C** (500 μL, 1 mM) in DMSO-d_6 to give a host-guest ratio of 1:10. A ^{1}H NMR spectrum was obtained at room temperature immediately.

Stoichiometry of binding of SO_4^{2-}, $H_2PO_4^-$, and CH_3COO^- to **C** in DMSO-d_6, that of SO_4^{2-} to **C**, **M**, **C′** and **C″** in DMSO-d_6/D_2O (*v/v* 19:1), and that of SO_4^{2-} to **C″** in DMSO-d_6 was determined by the continuous variation method (Job plot) with a 2.0 mM total concentration of the host and the guest. Changes in the chemical shift of the host protons (H_{bpy}, H_{dpm} or H_1) were followed.

Association constants were determined by ^{1}H NMR titration.[3] In a typical titration experiment, aliquots of 50 mM (for **C** and **C″**) or 100 mM (for **C′** and **M**) anion stock solutions were sequentially added to a 1 mM (for **C** and **C″**) or 2 mM (for **C′** and **M**) solution of the host (500 μL) at 298 K and mixed well, after which a ^{1}H NMR spectrum was obtained immediately. Changes in the chemical shift of the host protons were monitored. Association constants were obtained by a global fitting of binding isotherms using the online program Bindfit.[4] The full 1:2 binding model was used for calculating association constants of 1:2 stoichiometry (in a full 1:2 binding model, $K_{a1} \neq 4K_{a2}$, $\Delta\delta_{HG2} \neq 2\Delta\delta_{HG}$; $\Delta\delta_{HG}$ and $\Delta\delta_{HG2}$ represent the chemical shift changes of the host protons upon forming a 1:1 (HG) and 1:2 (HG_2) complex, respectively).[5] To further obtain the thermodynamic parameters of the sulfate binding towards **C**, ^{1}H NMR titration experiments in DMSO-d_6/D_2O (*v/v* 19:1) were conducted at 313 K, 328 K and 358 K to determine the corresponding association constants for constructing the van't Hoff plot.

In this case of Cl^- binding of **C**, only minimal chemical shift changes were observed, possibly due to the weak binding of the halide. Attempt to construct Job plot using the anion titration data failed. An alternative approach to determine binding stoichiometry pioneered by Thordarson was adopted, in which the covariance of the fit (cov_{fit}) of different fitting models is compared.[3] The 1:2 binding model was found to have a significantly lower cov_{fit} (2.2×10^{-5}) compared to that of the 1:1 model (8.1×10^{-4}).

Sulfate binding of **C** was also studied by UV-Vis spectroscopy and fluorescence spectroscopy with the same equipment and setting described in Section 4. Samples were contained in a quartz cuvette with a path length of 1 cm and 1.5 mL cell volume. A stock solution of TBA_2SO_4 (50 mM) was prepared in DMSO containing 125 μM of **C**. Aliquots of the sulfate stock solution was added to a solution of **C** (1000 μL, 125 μM in DMSO) in a quartz cuvette. The samples were mixed well at room temperature using a disposable pipette after each addition before a spectrum was collected. The samples for fluorescence studies were excited at 270 nm and emission spectra were collected from 290 nm to 700 nm. No significant changes were observed in the UV-Vis spectra of **C** upon the addition of SO_4^{2-}. A decrease in the normalized emission intensity at 460 nm was observed upon addition of SO_4^{2-}. Yet, such non-specific changes in the emission were also found when Cu^+ was added (see Section 4), and thus may not be useful to distinguish the binding of the different ions.

To investigate whether the addition of a chiral cation to the sulfate-bound complex of **C** can induce the formation of an enantiopure co-conformer over the other one, the L-valine methyl ester derived α-trimethylammonium (**L**$^+$) hexafluorophosphate was synthesized according to literature procedures.[6,7] and prepared as 50 mM stock solutions in DMSO containing 125 μM of **C** for circular dichroism (CD) spectroscopy and in DMSO-d_6 for ^{1}H NMR analysis, respectively. Spectra were recorded on JASCO J-815 CD spectropolarimeter equipped with a 150 W xenon arc lamp and collected from 250 nm to 400 nm. Samples for CD spectroscopy were contained in a quartz cuvette with a path length of 1 cm and 1.5 mL cell volume, and mixed well at room temperature using a disposable pipette after each addition before a spectrum was collected. No significant spectral changes were observed upon addition of **L**$^+$ by both CD and ^{1}H NMR spectroscopies.

To study the binding properties of **C** in the co-presence of Cu^+ and SO_4^{2-} in DMSO by ^{1}H NMR titration, a 50 mM $Cu(MeCN)_4(PF_6)$ stock solution in DMSO-d_6 was sequentially added to a 1 mM solution of **C** with 3 eq. of TBA_2SO_4 in DMSO-d_6. Upon addition of up to 1 mole eq. of Cu^+ (relative to **C**), no significant spectral changes were observed. Broad signals were observed when a further 0.5 eq to 2 eq. of Cu^+ (1.5 eq. to 3 eq. in total, relative to **C**) was added, suggesting the presence of several exchanging species with an exchange rate close to that of the NMR. The co-presence of Cu^+ and SO_4^{2-} ions hence likely resulted in the formation of both complexes, as well as related ion pairs, that are in exchange in solution. Further addition of Cu^+ of up to 6 eq. (relative to **C**) resulted in the emergence of a new set of signals with chemical shifts very close to that of [Cu**C**]$^+$, consistent with a shifting of the equilibrium towards the Cu(I)-catenane complexes as predicted by Le Chatelier's principle.

Phosphate (PO_4^{3-}) binding of **C** was also investigated by ^{1}H NMR titration in pre-dried DMSO-d_6 over 3 Å molecular sieves (water/DMSO *v/v* ~ 0.01%) due to the high basicity and hygroscopic nature of phosphate. TBA_3PO_4 was prepared by mixing phosphoric acid (H_3PO_4, 85 wt. % in H_2O) with 3 eq. of TBAOH (40 wt. % in H_2O), and the titration was performed in the same methods as described above. Upon addition of PO_4^{3-}, the urea NH protons were found disappeared, suggesting that the urea NH may have been deprotonated by the strongly basic phosphate. Further addition of up to 5 eq. of phosphate resulted in a new set of upfield-shifted, broad and overlapped signals, suggesting the presence of multiple exchanging species, likely with the urea NH deprotonated, that may be associated with the phosphate/hydrogen phosphate in the solution. Due to the possible presence of a (de)protonation equilibrium, the complex binding equilibrium could involve multiple species derived from the catenanes and phosphate, and thus interactions of the catenane host with PO_4^{3-} is likely more complicated than a simple host-guest binding.

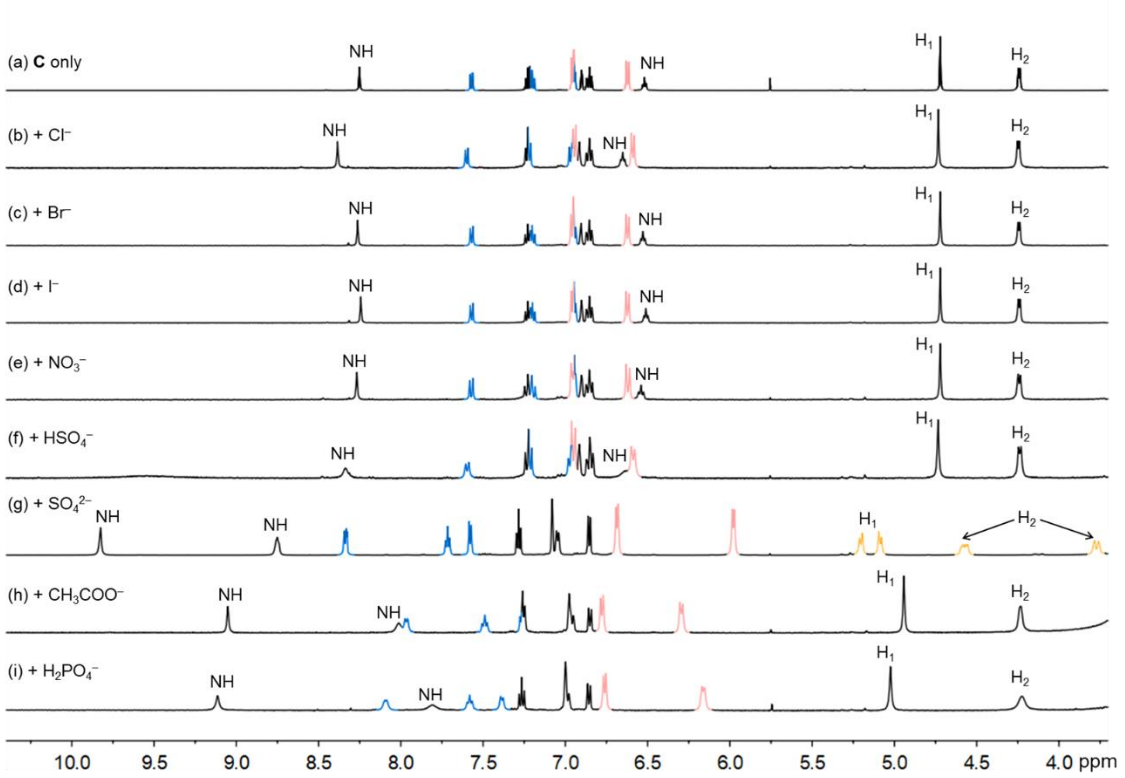

Supplementary Fig. 40. Partial ^{1}H NMR spectra (500 MHz, 298 K, DMSO-d_6) of a 1 mM solution of **C** in the presence of 10 eq. of different anions. Signals from H_{bpy} and H_{dpm} are highlighted in blue and pink respectively. Diastereotopic splitting of H_1 and H_2 in the sulfate complex is highlighted in orange.

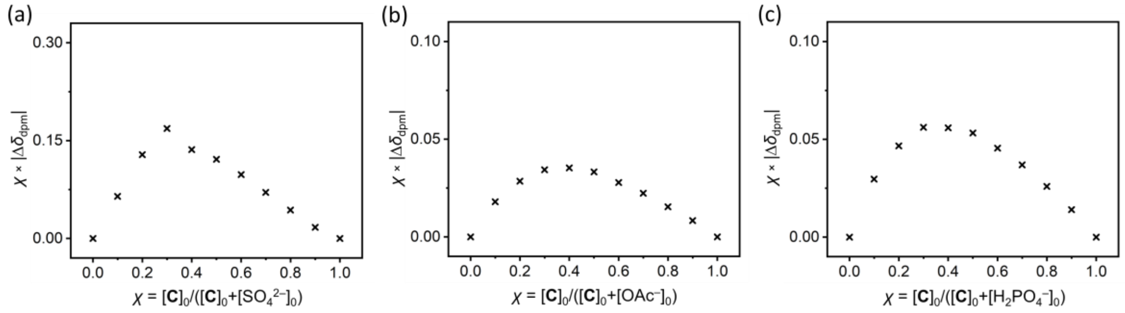

Supplementary Fig. 41. Job plots for the binding of **C** to (a) SO_4^{2-}; (b) CH_3COO^- and (c) $H_2PO_4^-$ in DMSO-d_6.

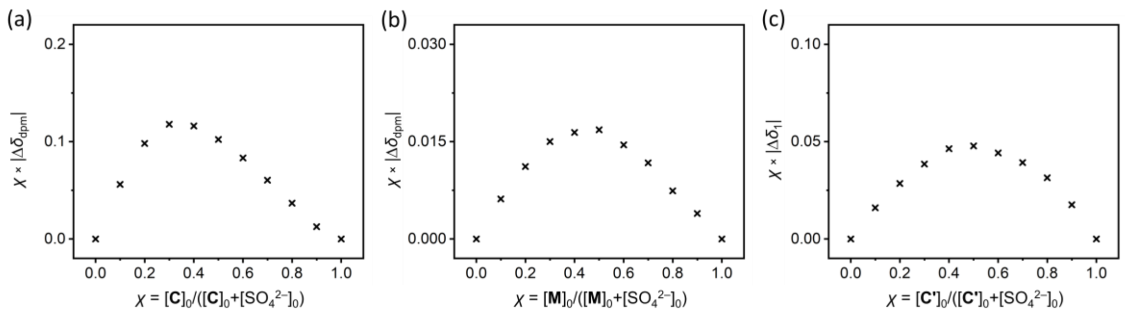

Supplementary Fig. 42. Job plots for the SO_4^{2-} binding in DMSO-d_6/D_2O (*v/v* 19:1) of (a) **C**; (b) **M** and (c) **C′**.

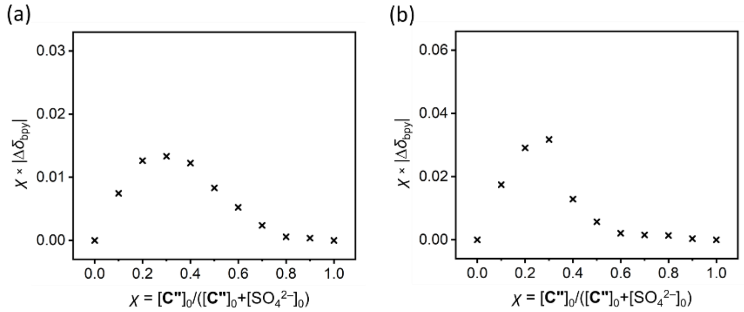

Supplementary Fig. 43. Job plots for the SO_4^{2-} binding of **C″** in (a) DMSO-d_6 and (b) DMSO-d_6/D_2O (*v/v* 19:1).

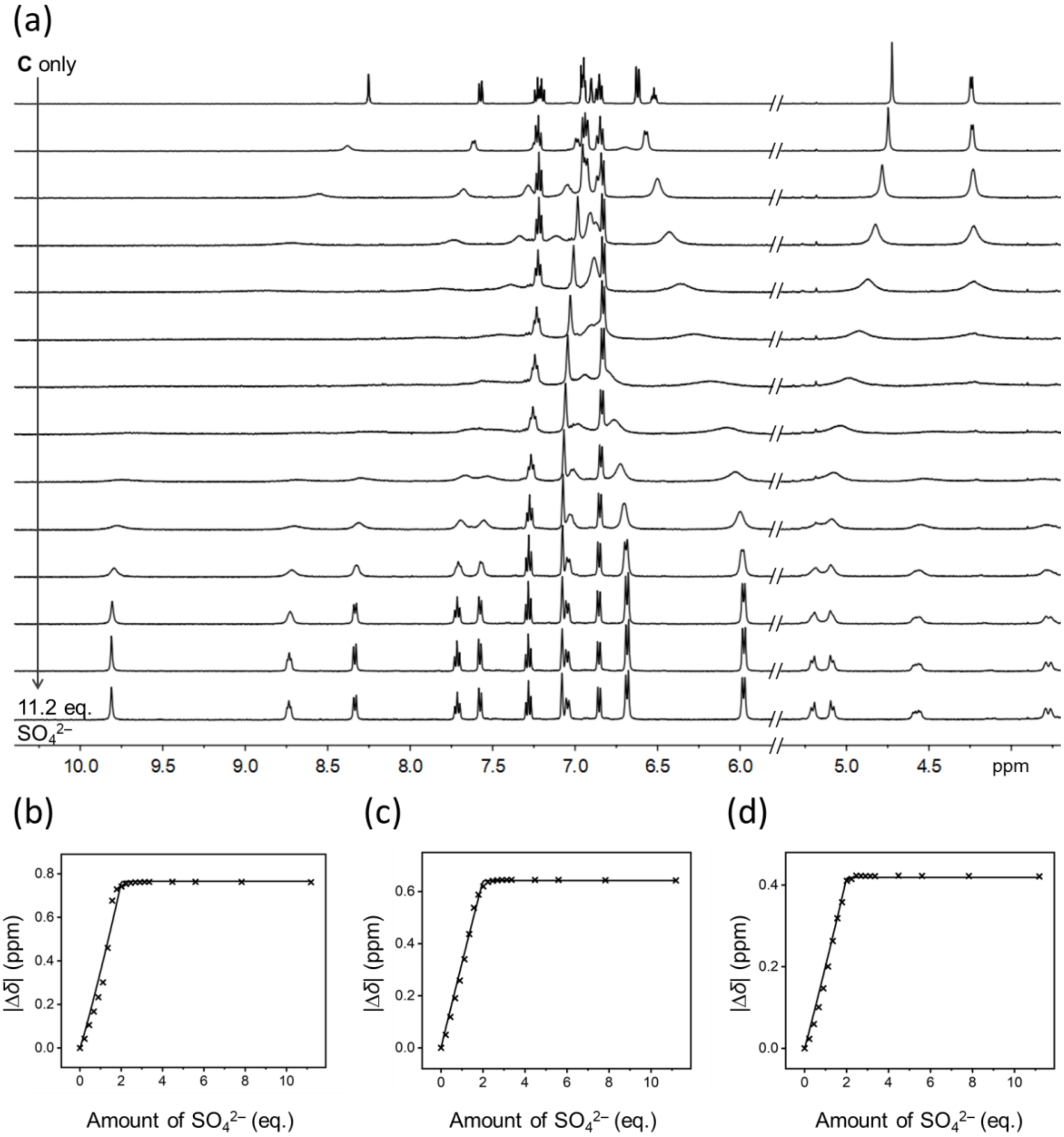

Supplementary Fig. 44. (a) Partial ^{1}H NMR spectra (500 MHz, 298 K, DMSO-d_6) of **C** in the presence of 0 eq. (top), 0.2 eq., 0.5 eq., 0.7 eq., 0.9 eq., 1.1 eq., 1.3 eq., 1.6 eq., 1.8 eq., 2.0 eq., 2.2 eq., 2.7 eq., 5.6 eq. and 11.2 eq. (bottom) of TBA sulfate. Binding isotherms obtained by monitoring chemical shift changes of signals initially at (b) 7.57 ppm, (c) 6.62 ppm and (d) 4.72 ppm. Association constants calculated: $K_1 > 10^5$ M^{-1}, $K_2 > 10^5$ M^{-1}.

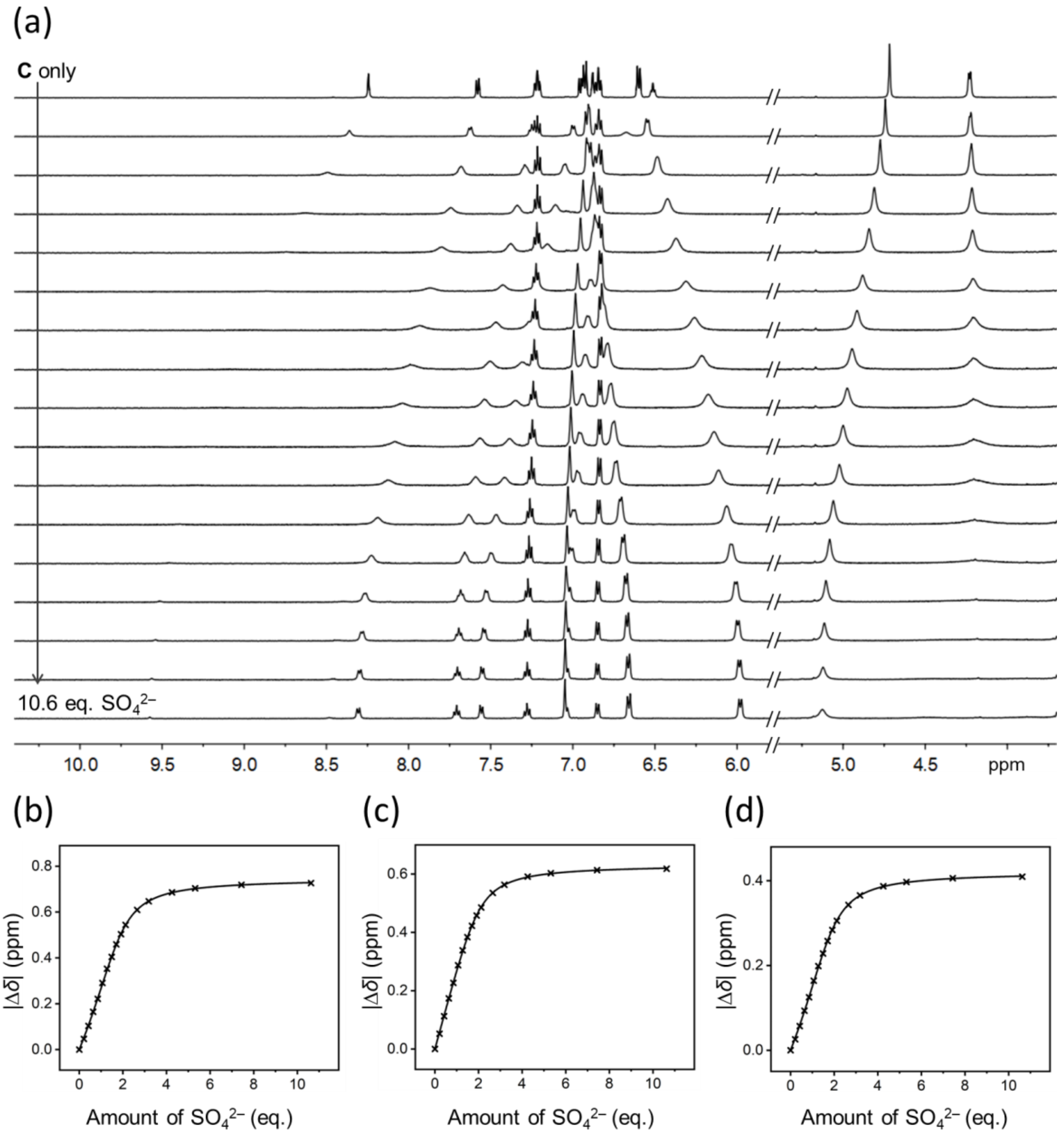

Supplementary Fig. 45. (a) Partial ^{1}H NMR spectra (500 MHz, 298 K, 19:1 (*v*/*v*) DMSO-d_6/D_2O) of **C** in the presence of 0 eq. (top), 0.2 eq., 0.4 eq., 0.6 eq., 0.8 eq., 1.1 eq., 1.3 eq., 1.5 eq., 1.7 eq., 1.9 eq., 2.1 eq., 2.7 eq., 3.2 eq., 4.2 eq., 5.3 eq., 7.4 eq. and 10.6 eq. (bottom) of TBA sulfate. Binding isotherms obtained by monitoring chemical shift changes of signals initially at (b) 7.58 ppm, (c) 6.60 ppm and (d) 4.72 ppm. Association constants calculated: K_1 = 21000 (± 3800) M^{-1}, K_2 = 3600 (± 100) M^{-1}.

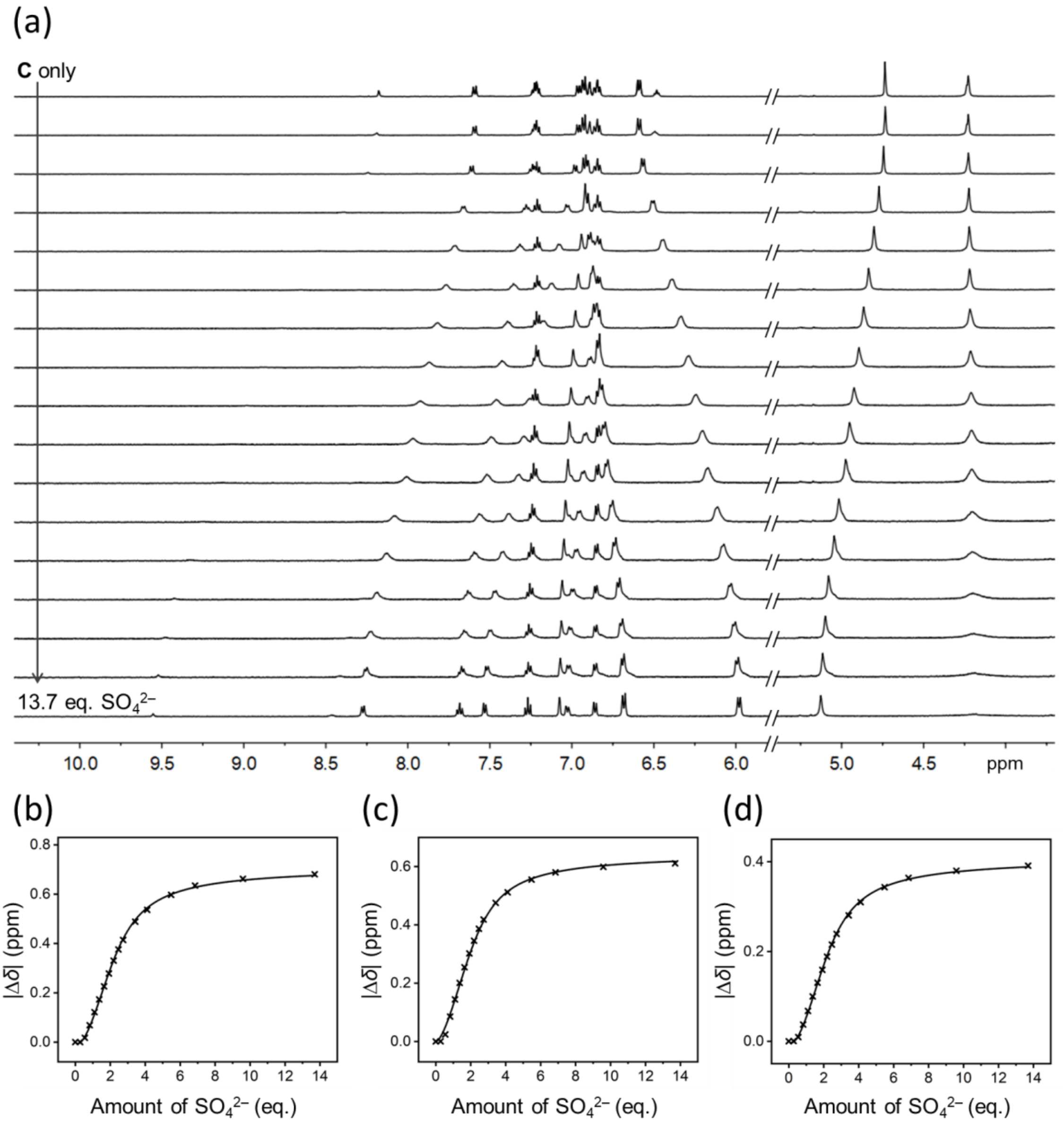

Supplementary Fig. 46. (a) Partial ^{1}H NMR spectra (500 MHz, 313 K, 19:1 (*v*/*v*) DMSO-d_6/D_2O) of **C** in the presence of 0 eq. (top), 0.3 eq., 0.5 eq., 0.8 eq., 1.1 eq., 1.4 eq., 1.6 eq., 1.9 eq., 2.2 eq., 2.5 eq., 2.7 eq., 3.4 eq., 4.1 eq., 5.5 eq., 6.8 eq., 9.6 eq. and 13.7 eq. (bottom) of TBA sulfate. Binding isotherms obtained by monitoring chemical shift changes of signals initially at (b) 7.59 ppm, (c) 6.59 ppm and (d) 4.74 ppm. Association constants calculated: K_1 = 3500 (± 750) M^{-1}, K_2 = 2700 (± 80) M^{-1}.

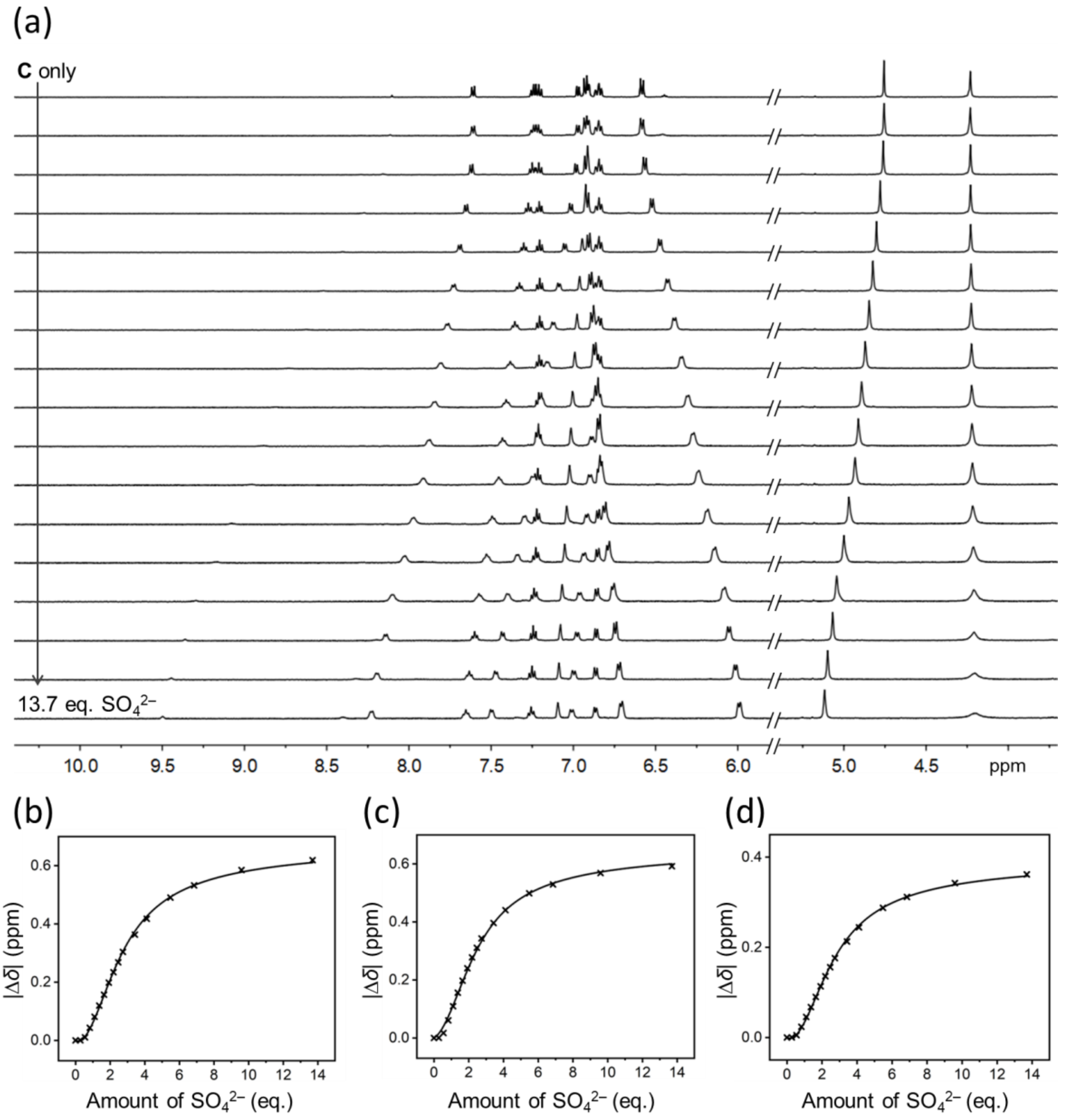

Supplementary Fig. 47. (a) Partial ^{1}H NMR spectra (500 MHz, 328 K, 19:1 (*v*/*v*) DMSO-d_6/D_2O) of **C** in the presence of 0 eq. (top), 0.3 eq., 0.5 eq., 0.8 eq., 1.1 eq., 1.4 eq., 1.6 eq., 1.9 eq., 2.2 eq., 2.5 eq., 2.7 eq., 3.4 eq., 4.1 eq., 5.5 eq., 6.8 eq., 9.6 eq. and 13.7 eq. (bottom) of TBA sulfate. Binding isotherms obtained by monitoring chemical shift changes of signals initially at (b) 7.61 ppm, (c) 6.58 ppm and (d) 4.76 ppm. Association constants calculated: K_1 = 4400 (± 1400) M^{-1}, K_2 = 1100 (± 40) M^{-1}.

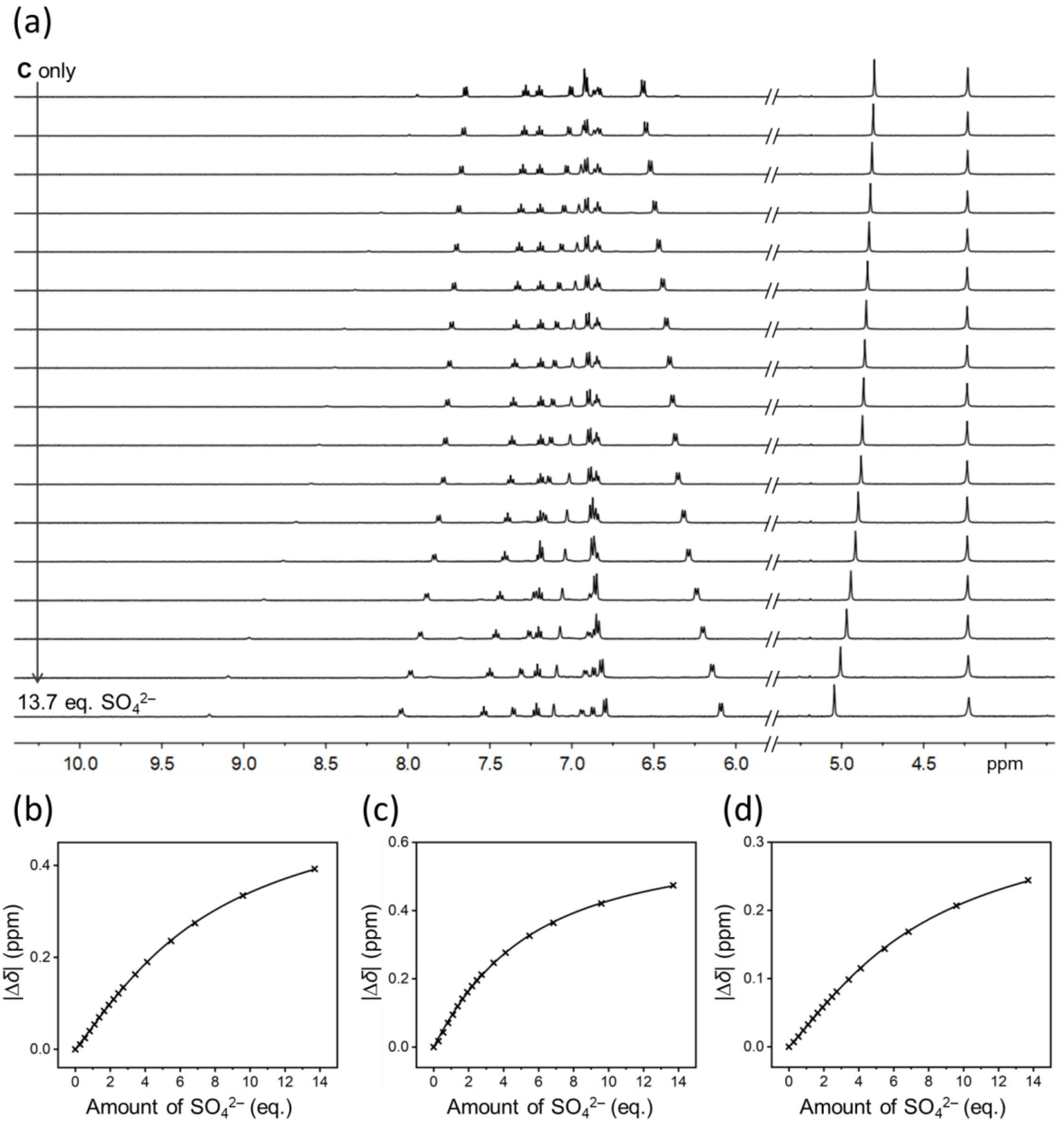

Supplementary Fig. 48. (a) Partial ^{1}H NMR spectra (500 MHz, 358 K, 19:1 (*v*/*v*) DMSO-d_6/D_2O) of **C** in the presence of 0 eq. (top), 0.3 eq., 0.5 eq., 0.8 eq., 1.1 eq., 1.4 eq., 1.6 eq., 1.9 eq., 2.2 eq., 2.5 eq., 2.7 eq., 3.4 eq., 4.1 eq., 5.5 eq., 6.8 eq., 9.6 eq. and 13.7 eq. (bottom) of TBA sulfate. Binding isotherms obtained by monitoring chemical shift changes of signals initially at (b) 7.65 ppm, (c) 6.56 ppm and (d) 4.80 ppm. Association constants calculated: K_1 = 530 (± 13) M^{-1}, K_2 = 170 (± 4) M^{-1}.

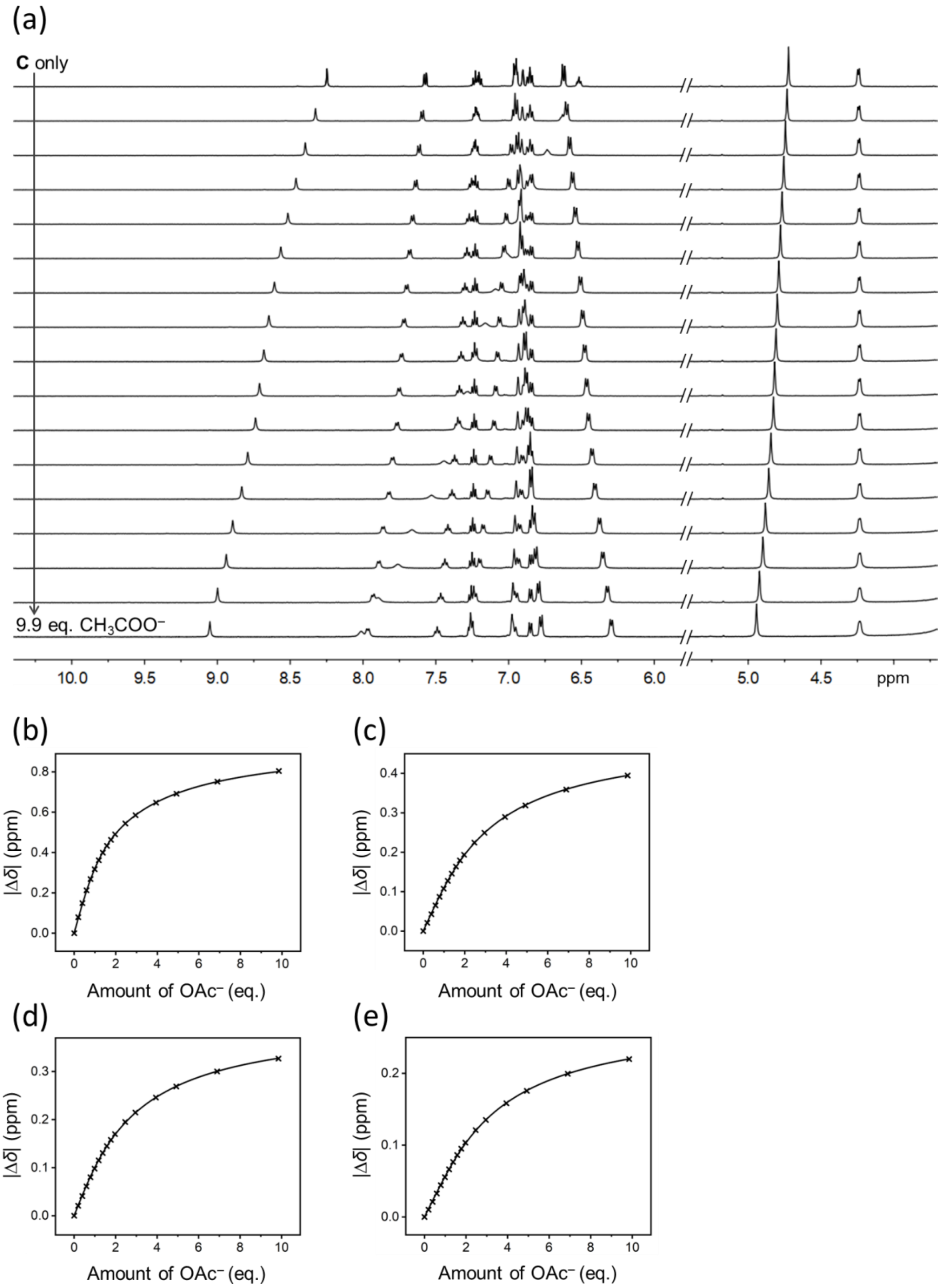

Supplementary Fig. 49. (a) Partial ^{1}H NMR spectra (500 MHz, 298 K, DMSO-d_6) of **C** in the presence of 0 eq. (top), 0.2 eq., 0.4 eq., 0.6 eq., 0.8 eq., 1.0 eq., 1.2 eq., 1.4 eq., 1.6 eq., 1.8 eq., 2.0 eq., 2.5 eq., 3.0 eq., 3.9 eq., 4.9 eq., 6.9 eq. and 9.9 eq. (bottom) of TBA acetate. Binding isotherms obtained by monitoring chemical shift changes of signals initially at (b) 8.25 ppm, (c) 7.57 ppm, (d) 6.62 ppm and (e) 4.72 ppm. Association constants calculated: K_1 = 7700 (± 210) M^{-1}, K_2 = 370 (± 4) M^{-1}.

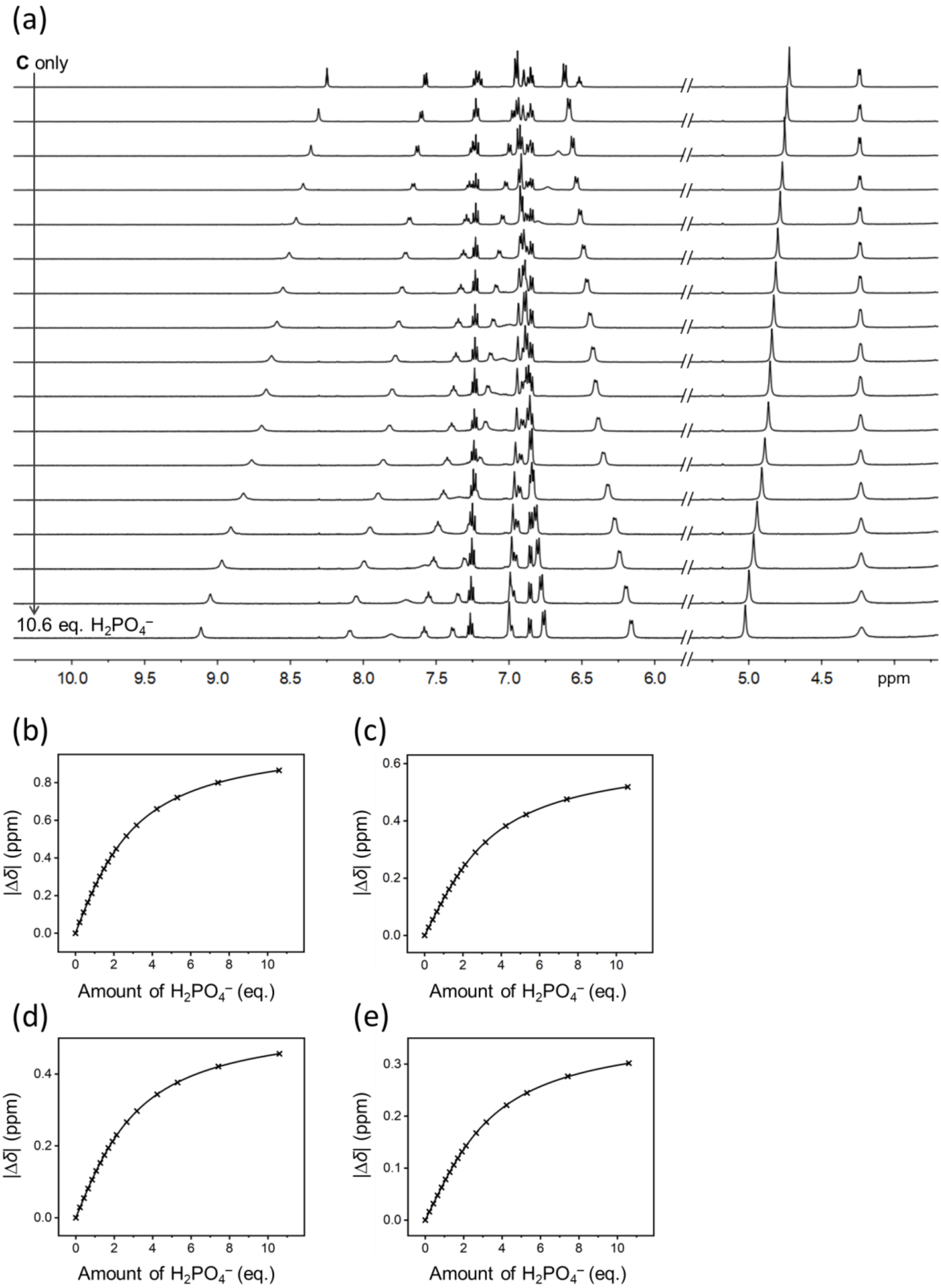

Supplementary Fig. 50. (a) Partial ^{1}H NMR spectra (500 MHz, 298 K, DMSO-d_6) of **C** in the presence of 0 eq. (top), 0.2 eq., 0.4 eq., 0.6 eq., 0.8 eq., 1.1 eq., 1.3 eq., 1.5 eq., 1.7 eq., 1.9 eq., 2.1 eq., 2.7 eq., 3.2 eq., 4.2 eq., 5.3 eq., 7.4 eq. and 10.6 eq. (bottom) of TBA dihydrogen phosphate. Binding isotherms obtained by monitoring chemical shift changes of signals initially at (b) 8.25 ppm, (c) 7.57 ppm, (d) 6.62 ppm and (e) 4.72 ppm. Association constants calculated: K_1 = 3000 (± 20) M^{-1}, K_2 = 390 (± 1) M^{-1}.

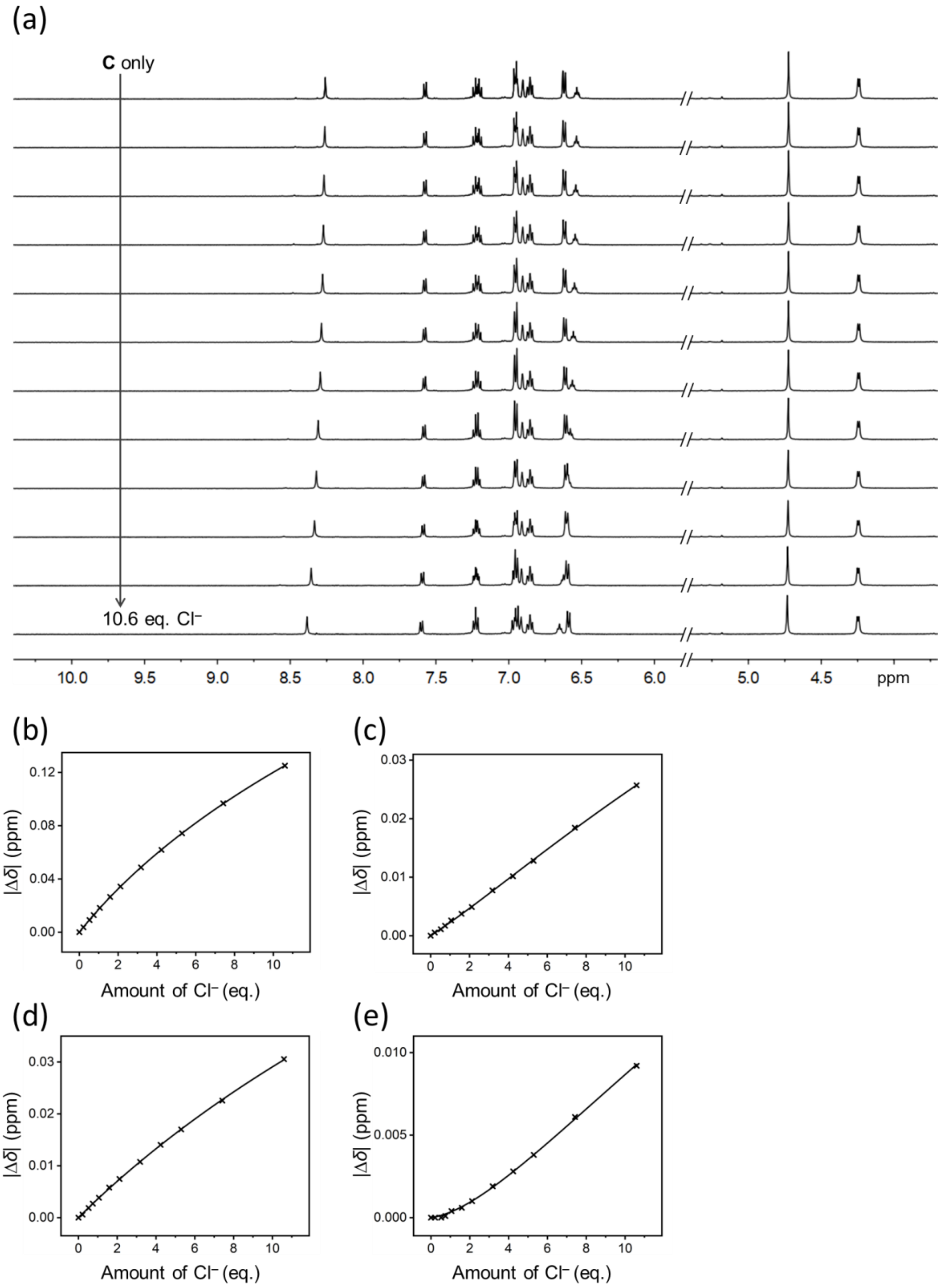

Supplementary Fig. 51. (a) Partial ^{1}H NMR spectra (500 MHz, 298 K, DMSO-d_6) of **C** in the presence of 0 eq. (top), 0.2 eq., 0.5 eq., 0.7 eq., 1.1 eq., 1.6 eq., 2.1 eq., 3.2 eq., 4.2 eq., 5.3 eq., 7.4 eq. and 10.6 eq. (bottom) of TBA chloride. Binding isotherms obtained by monitoring chemical shift changes of signals initially at (b) 8.26 ppm, (c) 7.57 ppm, (d) 6.62 ppm and (e) 4.72 ppm. Association constants calculated: K_1 = 160 (± 1) M^{-1}, K_2 = 3 (± 0.02) M^{-1}.

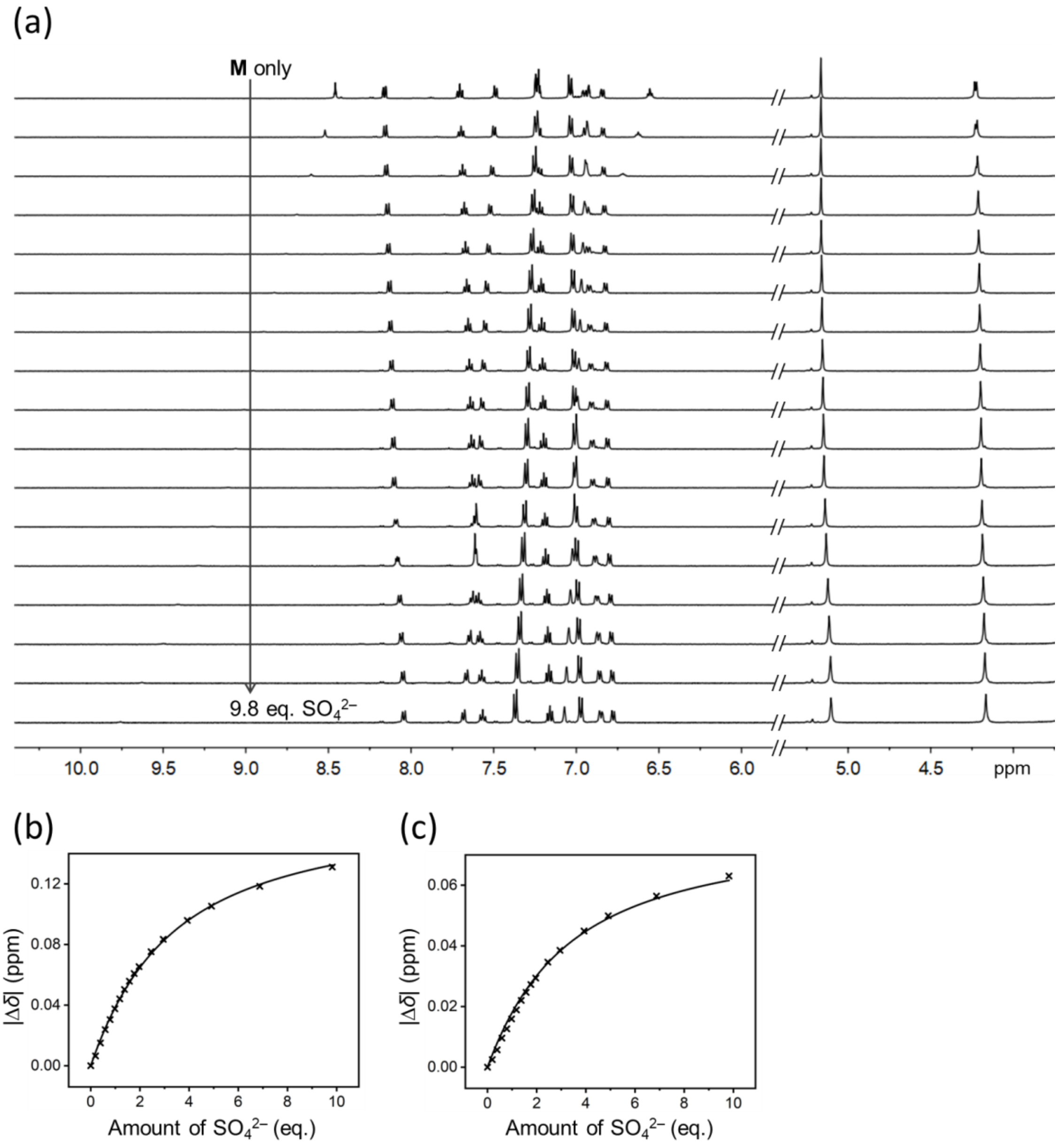

Supplementary Fig. 52. (a) Partial ^{1}H NMR spectra (500 MHz, 298 K, 19:1 (*v*/*v*) DMSO-d_6/D_2O) of **M** in the presence of 0 eq. (top), 0.2 eq., 0.4 eq., 0.6 eq., 0.8 eq., 1.0 eq., 1.2 eq., 1.4 eq., 1.6 eq., 1.8 eq., 2.0 eq., 2.5 eq., 2.9 eq., 3.9 eq., 4.9 eq., 6.9 eq. and 9.8 eq. (bottom) of TBA sulfate. Binding isotherms obtained by monitoring chemical shift changes of signals initially at (b) 7.24 ppm and (c) 7.04 ppm. Association constant calculated: K = 360 (± 7) M^{-1}.

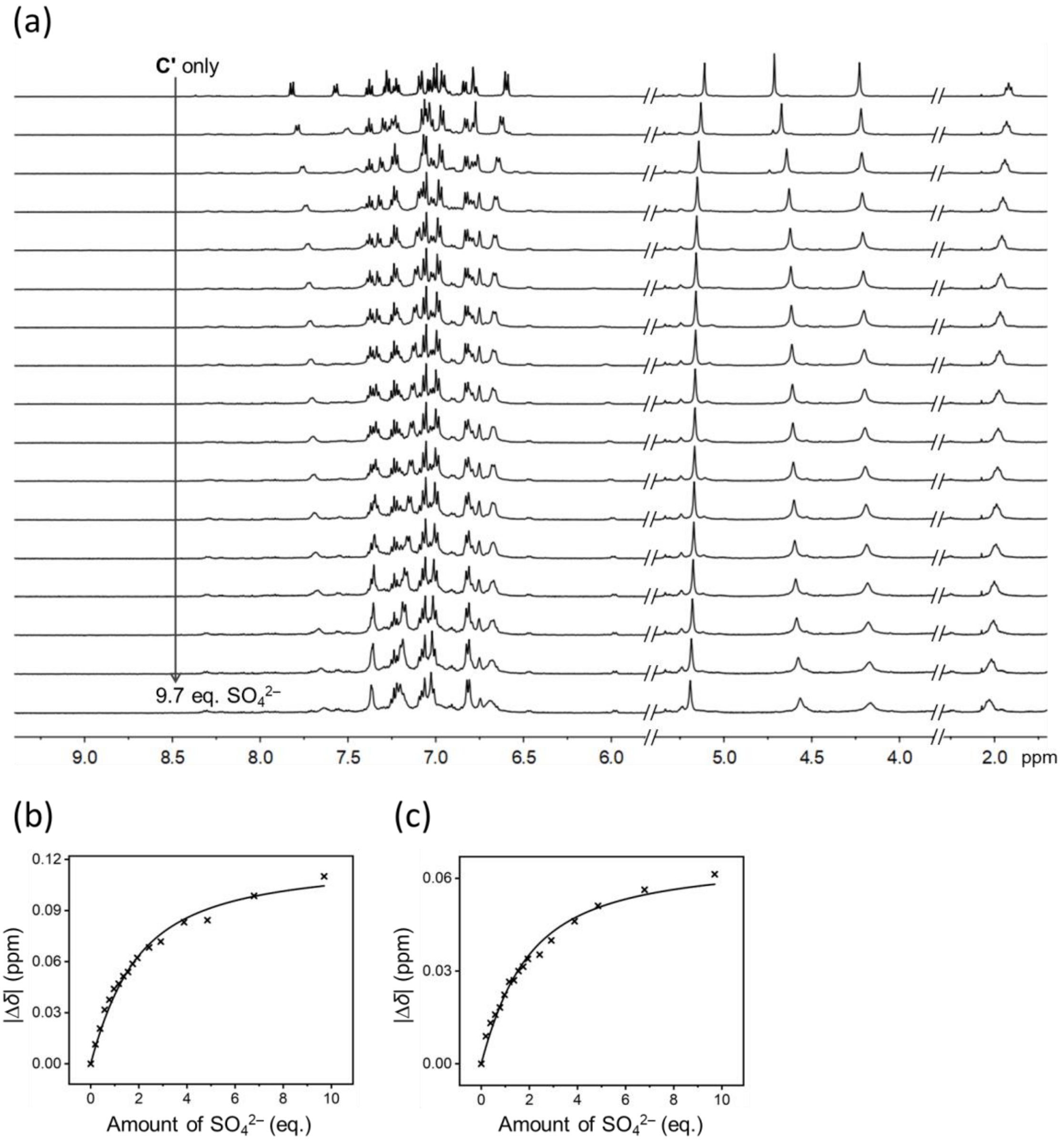

Supplementary Fig. 53. (a) Partial ^{1}H NMR spectra (500 MHz, 298 K, 19:1 (*v*/*v*) DMSO-d_6/D_2O) of **C′** in the presence of 0 eq. (top), 0.2 eq., 0.4 eq., 0.6 eq., 0.8 eq., 1.0 eq., 1.2 eq., 1.4 eq., 1.6 eq., 1.7 eq., 1.9 eq., 2.4 eq., 2.9 eq., 3.9 eq., 4.9 eq., 6.8 eq. and 9.7 eq. (bottom) of TBA sulfate. Binding isotherms obtained by monitoring chemical shift changes of signals initially at (b) 4.23 ppm and (c) 1.92 ppm. Association constant calculated: K = 370 (± 22) M^{-1}.

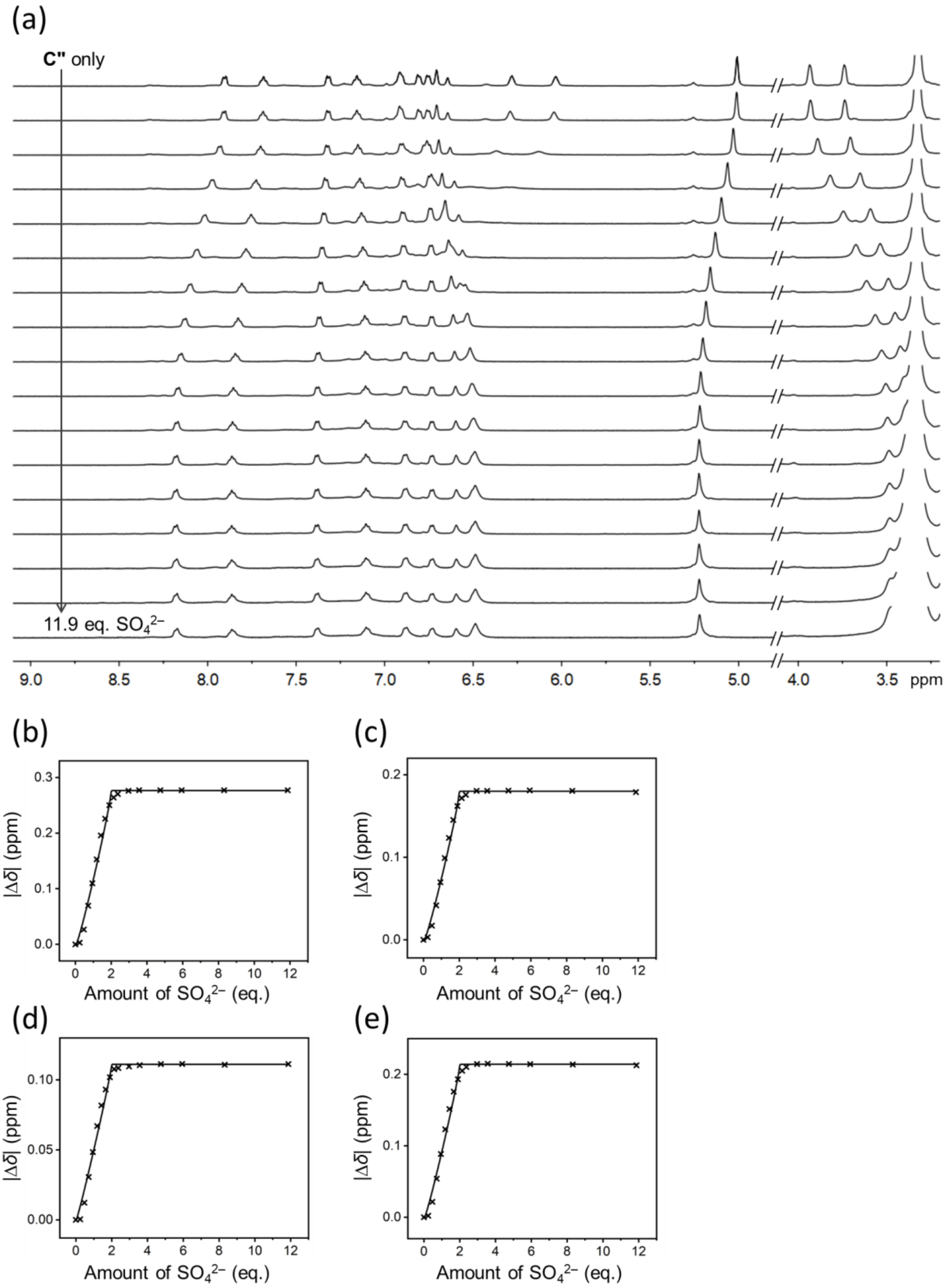

Supplementary Fig. 54. (a) Partial ^{1}H NMR spectra (500 MHz, 298 K, DMSO-d_6) of **C″** in the presence of 0 eq. (top), 0.2 eq., 0.5 eq., 0.7 eq., 1.0 eq., 1.2 eq., 1.4 eq., 1.7 eq., 1.9 eq., 2.1 eq., 2.4 eq., 3.0 eq., 3.6 eq., 4.8 eq., 5.9 eq., 8.3 eq. and 11.9 eq. (bottom) of TBA sulfate. Binding isotherms obtained by monitoring chemical shift changes of signals initially at (b) 7.90 ppm, (c) 7.68 ppm, (d) 6.71 ppm and (e) 5.01 ppm. Association constants calculated: $K_1 > 10^5$ M^{-1}, $K_2 > 10^5$ M^{-1}.

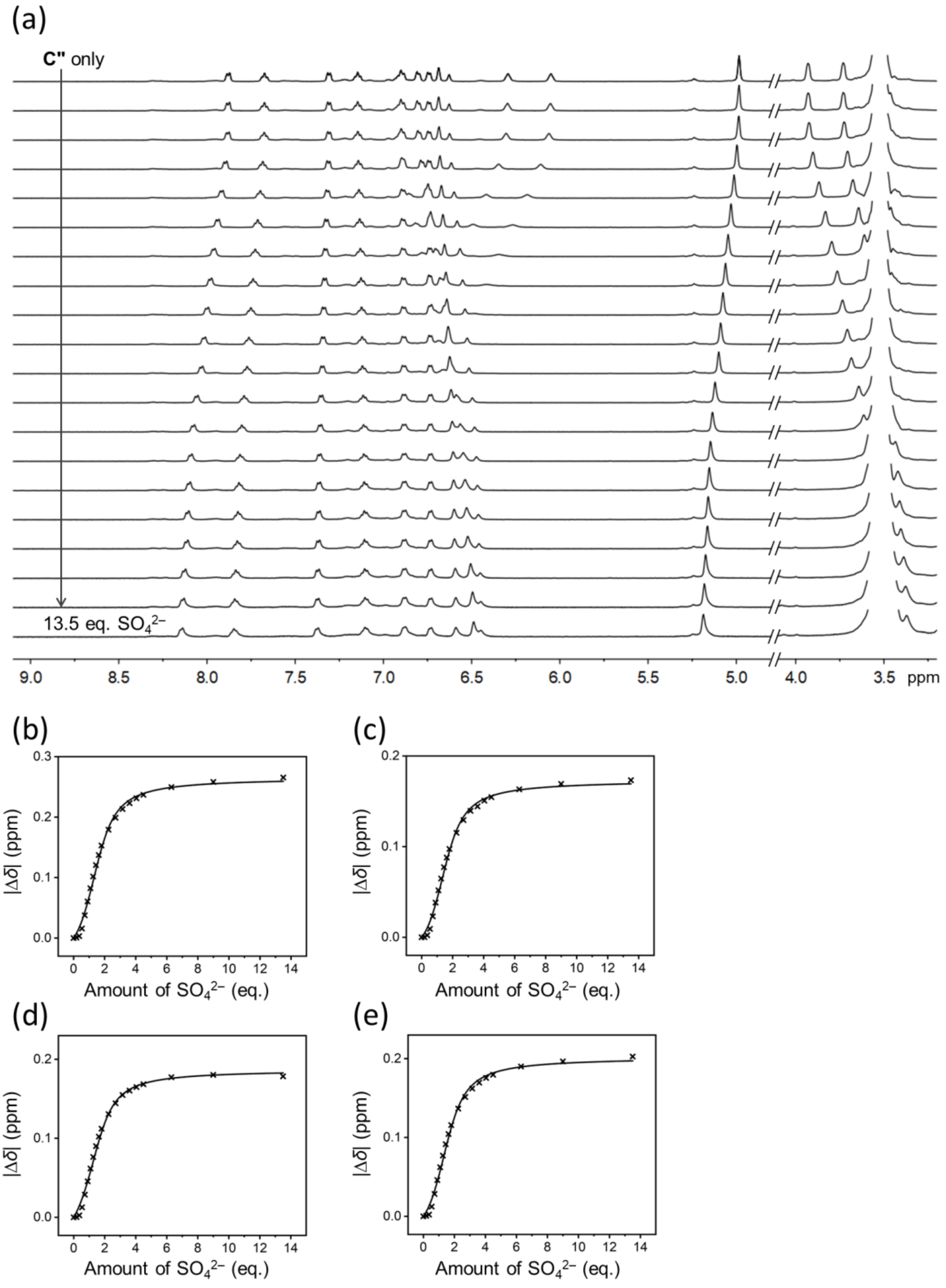

Supplementary Fig. 55. (a) Partial ^{1}H NMR spectra (500 MHz, 298 K, 19:1 (*v*/*v*) DMSO-d_6/D_2O) of **C″** in the presence of 0 eq. (top), 0.2 eq., 0.4 eq., 0.5 eq., 0.7 eq., 0.9 eq., 1.1 eq., 1.3 eq., 1.4 eq., 1.6 eq., 1.8 eq., 2.3 eq., 2.7 eq., 3.2 eq., 3.6 eq., 4.1 eq., 4.5 eq., 6.3 eq., 9.0 eq. and 13.5 eq. (bottom) of TBA sulfate. Binding isotherms obtained by monitoring chemical shift changes of signals initially at (b) 7.88 ppm, (c) 7.67 ppm, (d) 6.63 ppm and (e) 4.98 ppm. Association constants calculated: K_1 = 18000 (± 11000) M^{-1}, K_2 = 3000 (± 210) M^{-1}.

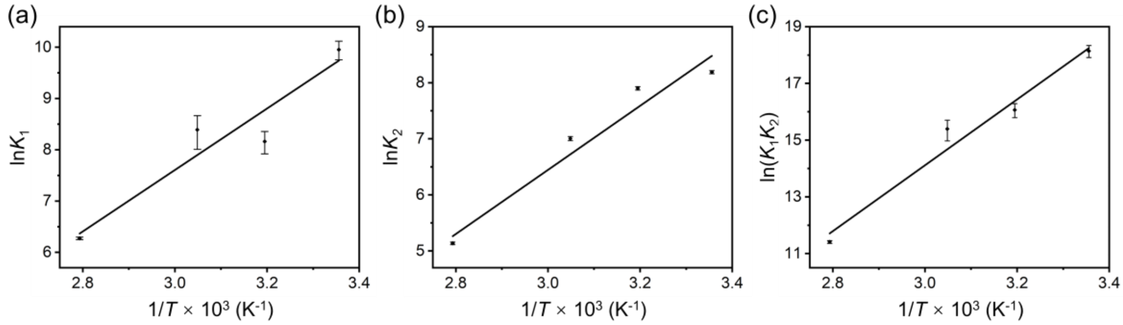

Supplementary Fig. 56. Van't Hoff plots for (a) the first SO_4^{2-} binding of **C** (ΔH_1 = –12(±3) kcal·mol^{-1}, ΔS_1 = –21(±8) cal·mol^{-1}·K^{-1}, R^2 of linear fitting = 0.90), (b) the second SO_4^{2-} binding of **C** (ΔH_2 = –11(±2) kcal·mol^{-1}, ΔS_2 = –21(±4) cal·mol^{-1}·K^{-1}, R^2 of linear fitting = 0.96) and (c) the overall 1:2 SO_4^{2-} binding of **C** ($\Delta H°$ = –23(±3) kcal·mol^{-1}, $\Delta S°$ = –41(±9) cal·mol^{-1}·K^{-1}, R^2 of linear fitting = 0.97) in DMSO-d_6/D_2O (*v/v* 19:1).

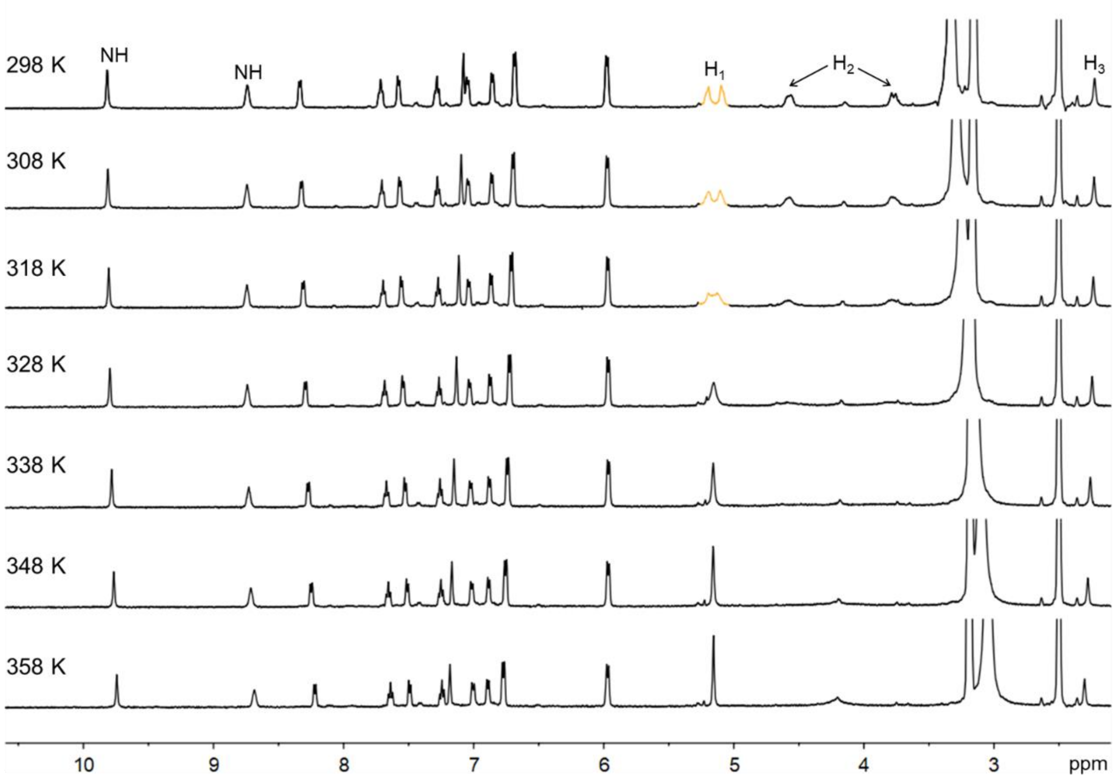

Supplementary Fig. 57. Partial ^{1}H NMR spectra (500 MHz, DMSO-d_6) of **C** (1 mM) in the presence of 3 eq. of TBA sulfate obtained at temperature from 298 K to 358 K. Diastereotopic splitting of H_1 is highlighted in orange. Coalescence temperature of signal H_1 T_c = 318 K, $\Delta G^‡$ = 15.7 kcal·mol^{-1}.

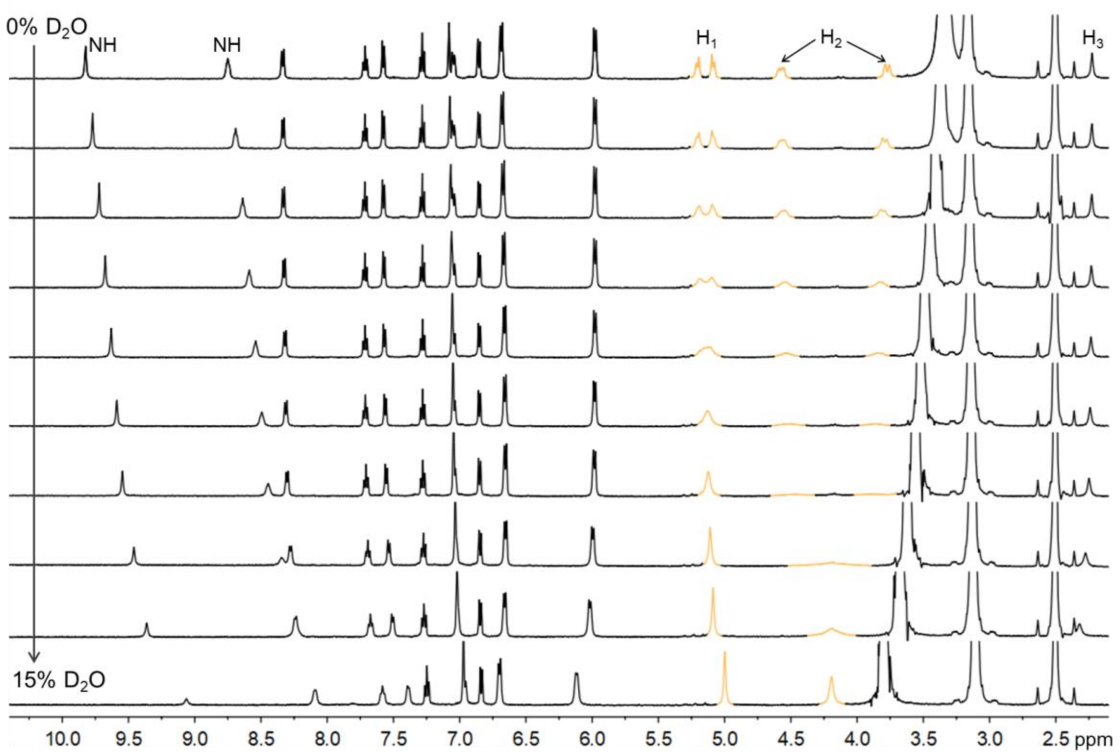

Supplementary Fig. 58. Partial ^{1}H NMR spectra (500 MHz, 298 K, DMSO-d_6) of **C** (1 mM) with 10 eq. of TBA sulfate in the presence of 0% (top) to 1%, 2%, 3%, 4%, 5%, 6%, 8%, 10% and 15% (bottom) D_2O (*v*/*v*).

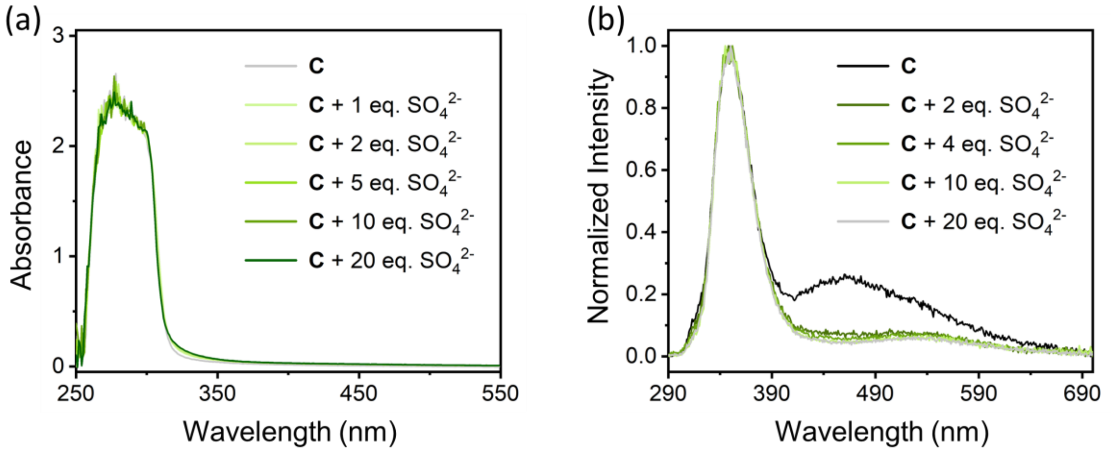

Supplementary Fig. 59. (a) UV-Vis spectra and (b) normalized fluorescence spectra of 125 μM solutions of **C** in DMSO at 298 K in the presence of 0 to 20 eq. of SO_4^{2-}.

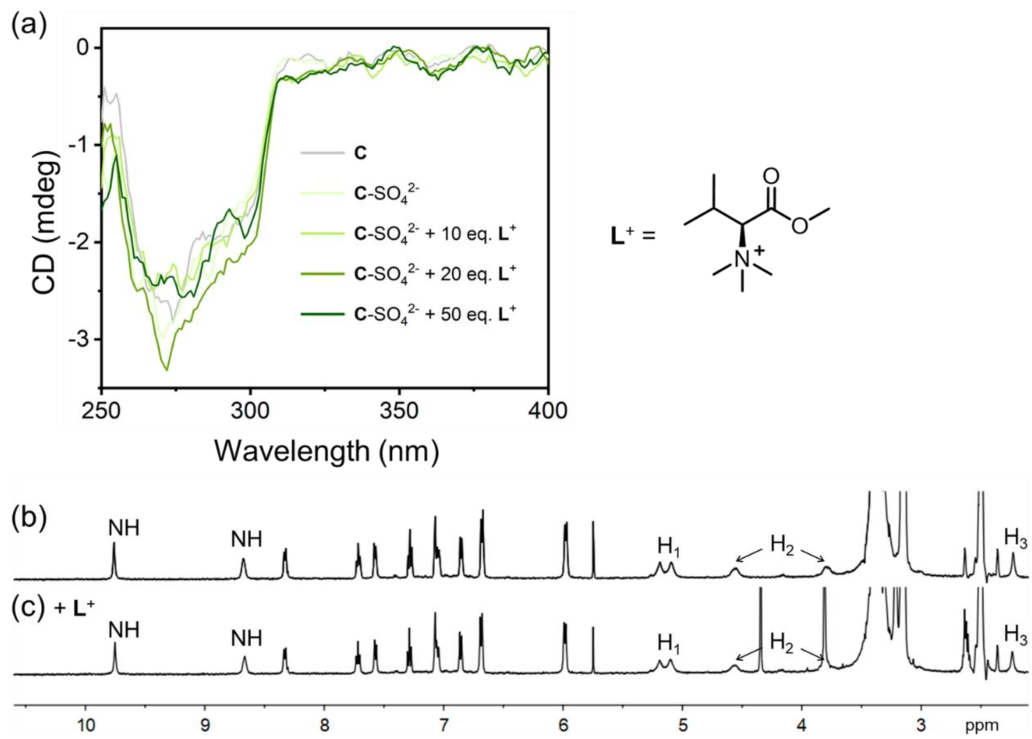

Supplementary Fig. 60. (a) CD spectra of 125 μM solutions of **C** in DMSO at 298 K with 10 eq. of TBA sulfate in the presence of 0 to 50 eq. of chiral cation $\mathbf{L}^+$, and partial ^{1}H NMR spectra (500 MHz, 298 K, DMSO-d_6) of **C** (1 mM) with 3 eq. of TBA sulfate (b) before and (c) after the addition of 6 eq. of $\mathbf{L}^+$.

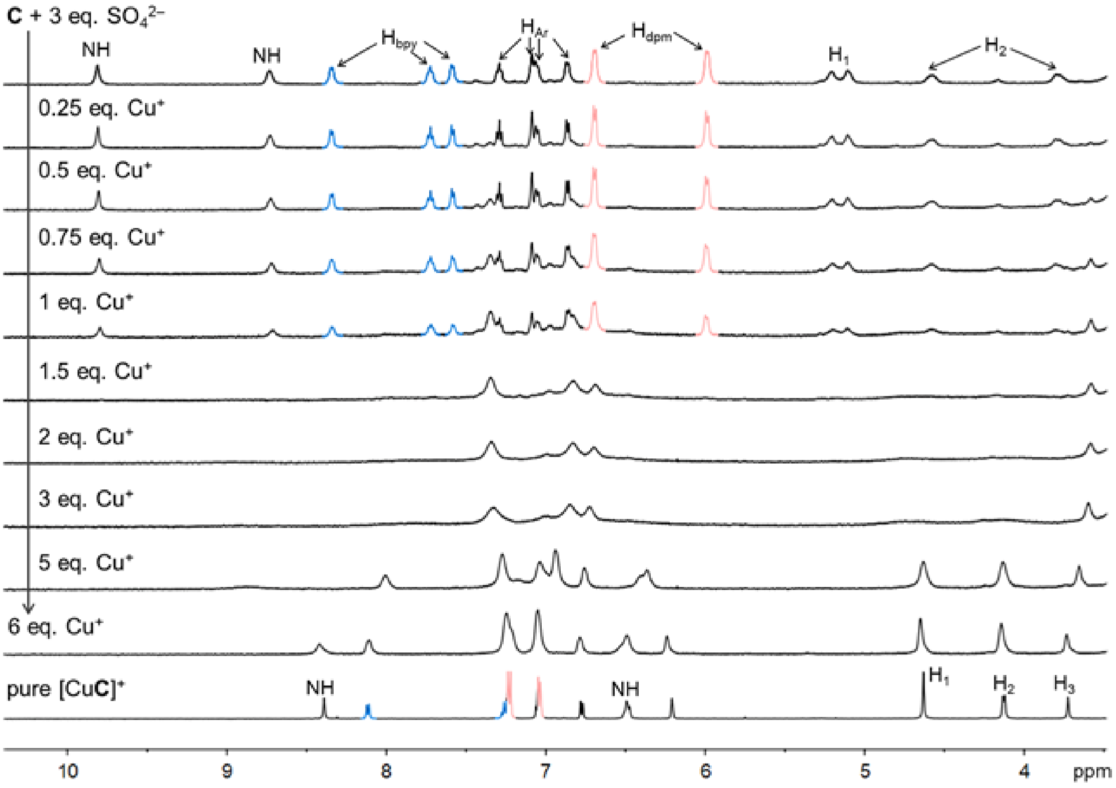

Supplementary Fig. 61. Partial ^{1}H NMR spectra (500 MHz, 298 K, DMSO-d_6) of **C** (1 mM) with 3 eq. of TBA sulfate in the presence of 0 eq. to 6 eq. of Cu^+, compared with that of pure $[Cu\mathbf{C}]^+$ (bottom).

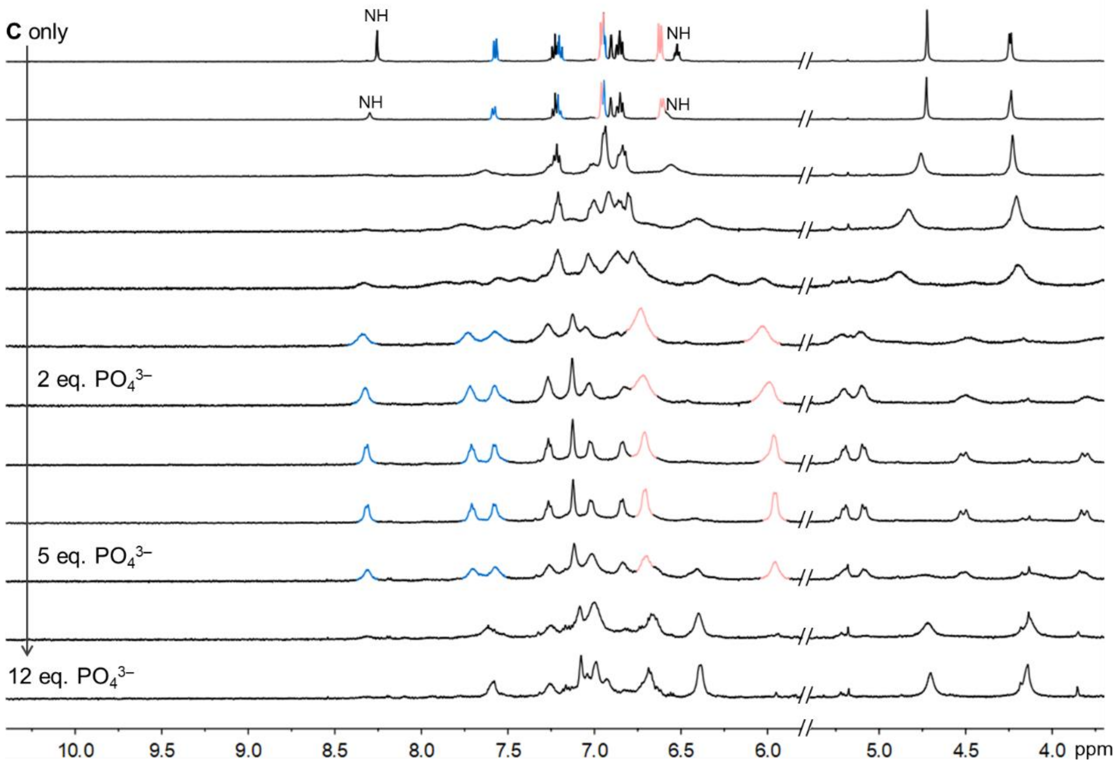

Supplementary Fig. 62. Partial ^{1}H NMR spectra (500 MHz, 298 K, molecular sieve-dried DMSO-d_6) of **C** (1 mM) in the presence of 0 eq. (top), 0.25 eq., 0.5 eq., 0.75 eq., 1 eq., 1.5 eq., 2 eq., 2.5 eq., 3 eq., 5 eq., 8 eq. and 12 eq. (bottom) of TBA phosphate. Signals from H_{bpy} and H_{dpm} are highlighted in blue and pink respectively.

5. Computational Studies on Anion Binding of C

The initial configurations for **C**·X^{n-} and **C**·2X^{n-} (X^{n-} = SO_4^{2-}, $H_2PO_4^-$, CH_3COO^- and Cl^-) and visualization of optimized structures were generated using the Avogadro software.[8] The resulting empirical optimized configurations were further refined using density functional theory (DFT) calculations performed with Gaussian 16 (revision A.03).[9]

Geometry optimizations and single-point energy calculations were carried out using the spin-restricted hybrid density functional B3LYP,[10–12] and the 6-31G (d,p) basis set was employed for all atoms. The solvation effects of DMSO (ε = 46.826) were taken into account using the conductor-like polarizable continuum model (CPCM).[13,14] During the structural optimization step, a convergence criterion of 10^{-8} for the density matrix was applied. Vibrational analyses were conducted to validate the optimized structures.

Supplementary Table 1. Calculated thermodynamic parameters of the simulated anion binding process of **C** in DMSO (T = 298 K).

Anion	SO_4^{2-}	$H_2PO_4^-$	CH_3COO^-	Cl^-
K_1 (M^{-1})	7.91×10^{22}	3548	6990	154
ΔG_1 (kcal·mol^{-1})	-31.22	-4.84	-5.24	-2.98
ΔH_1 (kcal·mol^{-1})	-42.48	-15.27	-11.50	-5.53
$T\Delta S_1$ (kcal·mol^{-1})	-11.25	-10.43	-6.26	-2.55
K_2 (M^{-1})	7.54×10^{8}	376	393	3
ΔG_2 (kcal·mol^{-1})	-12.10	-3.51	-3.54	-0.71
ΔH_2 (kcal·mol^{-1})	-31.58	-9.19	-14.79	-6.49
$T\Delta S_2$ (kcal·mol^{-1})	-19.47	-5.68	-11.25	-5.78
β (M^{-2})	5.96×10^{31}	1.33×10^{6}	2.75×10^{6}	509
ΔG (kcal·mol^{-1})	-43.33	-8.35	-8.78	-3.69
ΔH (kcal·mol^{-1})	-74.06	-24.46	-26.29	-12.02
$T\Delta S$ (kcal·mol^{-1})	-30.73	-16.11	-17.51	-8.33

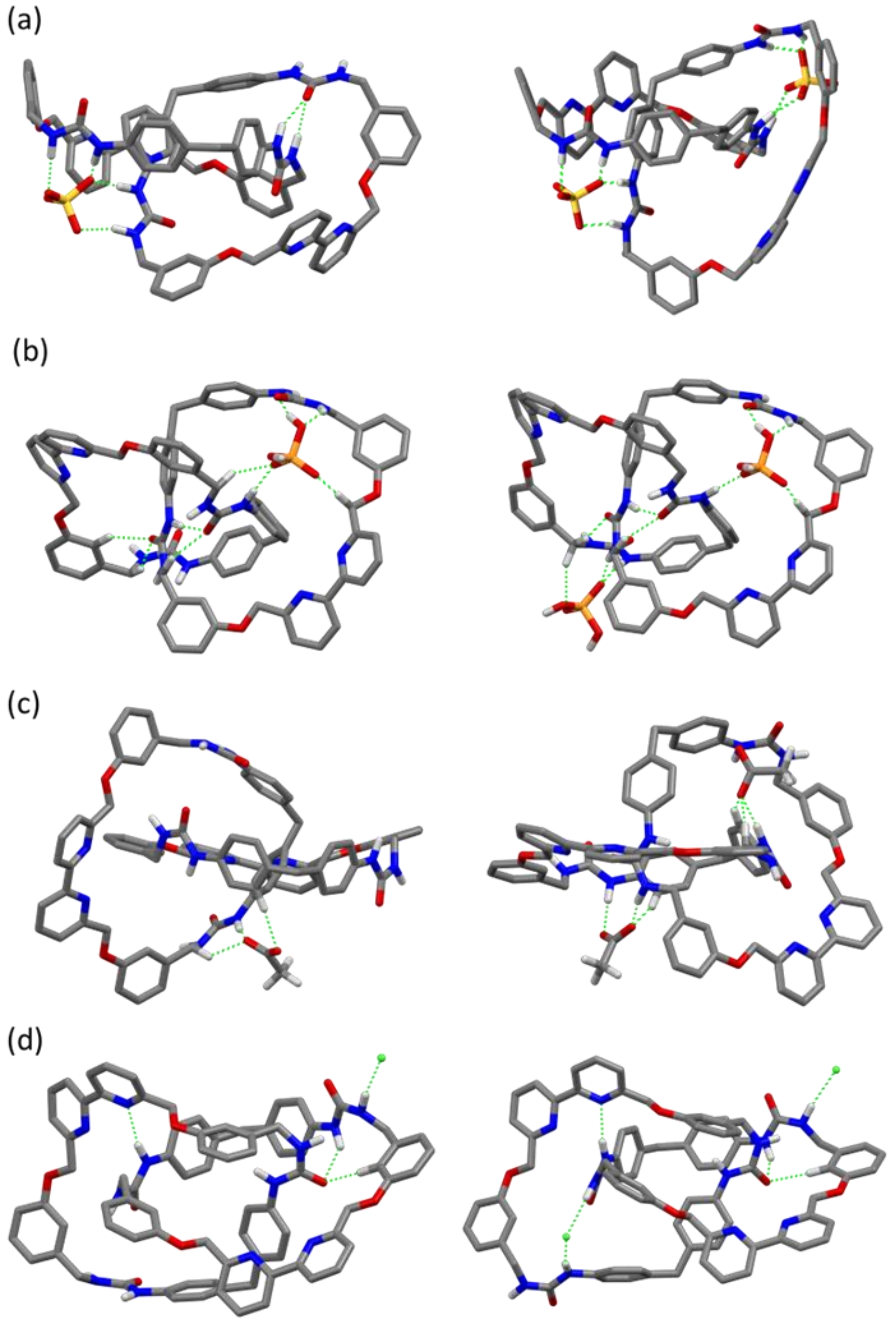

Supplementary Fig. 63. Selected optimized structures of 1:1 (left) and 1:2 (right) complexes of **C** with (a) SO_4^{2-}, (b) $H_2PO_4^-$, (c) CH_3COO^- and (d) Cl^-. Hydrogen bonds are highlighted in green. Hydrogen atoms of C−H not involved in hydrogen bonding are omitted.

6. X-ray Crystallography

Crystals suitable for X-ray diffraction were mounted on a MiTeGen dual-thickness 75-micron micro-mount and placed under a cold stream of nitrogen (Oxford). Single-crystal X-ray diffraction measurements were recorded on a Bruker D8 VENTURE Duo FIXED-CHI X-Ray Diffractometer using an IµS micro-focus Mo-K_α radiation (λ = 0.71073 Å) with a Quazar multilayer optics. Data collection was conducted with the APEX3 v2019.3-2click4 (Bruker Nano, 2019) program. Cell refinement and data reduction were performed with the SAINT V8.38A (Bruker AXS Inc., 2017) program. The structure was solved using XT 2014/5 in the APEX3 suite[15] and refined with SHELXL2018/3.[16] Hydrogen atoms were placed in idealized positions and were set riding on the respective parent atoms. All non-hydrogen atoms were refined with anisotropic thermal parameters. The structure was refined by weighted least squares refinement on F^2 to convergence. All e.s.d.'s (except the e.s.d. in the dihedral angle between two least-square planes) are estimated using the full covariance matrix. Restraints were applied due to the disordered nature of the DMF solvent molecules, TBA cations, and the sulfate dianion. The catenane molecules were refined as residues with the use of the SAME command due to the high degree of freedom of the molecules. The X-ray crystallographic data has been deposited at the Cambridge Crystallographic Data Centre (CCDC), under the deposition number CCDC 2245688. The data can be obtained free of charge from the Cambridge Crystallographic Data Center (https://www.ccdc.cam.ac.uk/structures/).

Supplementary Table 2. Selected crystallographic data and structure refinement summary of **C**-TBA_2SO_4.

Empirical formula	$C_{126}H_{172}N_{18}O_{16}S$
Formula weight	2226.87
Temperature (K)	146
Crystal system	Monoclinic
Space group	$P2_1/n$
a (Å)	17.967(2)
b (Å)	31.193(3)
c (Å)	23.024(3)
α (°)	90
β (°)	100.643(3)
γ (°)	90
Volume ($Å^3$)	12682(3)
Z	4
Density ($g\cdot cm^{-3}$)	1.166
Absorption coefficient (mm^{-1})	0.09
F(000)	4792.0
Crystal size (mm^3)	0.23 × 0.11 × 0.07
Radiation (Å)	Mo $K\alpha$ (λ = 0.71073 Å)
θ_{min}, θ_{max} (°)	2.2, 24.2
Index ranges	$-20 \leq h \leq 20$, $-35 \leq k \leq 35$, $-26 \leq l \leq 26$
Reflections collected	207082
Independent reflections	20382
R_{int}	0.155
Data/Restraints/Parameters	20382/11406/2838
Absorption correction	Multi-scan
Final R indexes [$I \geq 2\sigma(I)$][a,b]	R_1 = 0.0887, wR_2 = 0.2884
Largest diff. peak/hole ($eÅ^{-3}$)	0.358/-0.406
Restrained goodness of fit (GOF)[c]	1.014

[a] $R_1 = \Sigma||F_o|-|F_c||/\Sigma|F_o|$. [b] $wR_2 = \{\Sigma w(F_o^2-F_c^2)^2/\Sigma w(F_o^2)^2\}^{1/2}$.

[c] GOF = $\{\Sigma w((F_o^2-F_c^2)^2)]/(n-p)\}^{1/2}$, where n = number of reflections and p = total number of parameters refined.

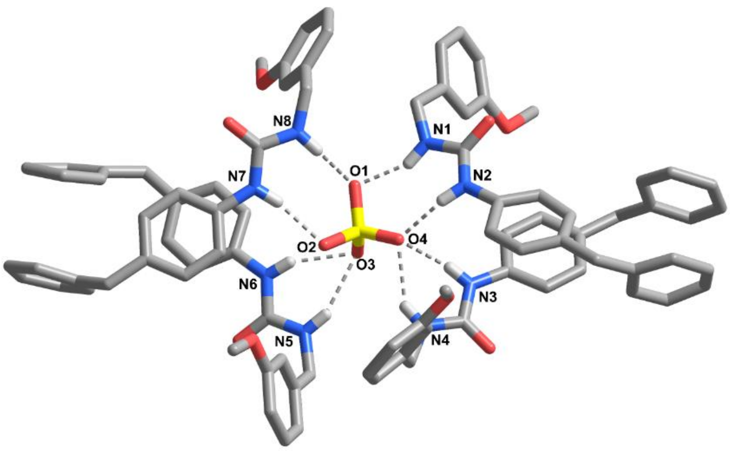

Supplementary Fig. 64. Hydrogen bonds found around the SO_4^{2-} ion. Only half of each catenane molecule is shown for clarity.

Supplementary Table 3. Hydrogen-bond geometry around the SO_4^{2-} ion.

D–H···A	d(D–H)^a (Å)	d(H···A) (Å)	d(D···A) (Å)	∠(DHA) (°)
N1–H1···O1^i	0.880	1.937	2.755(3)	153.9
N2–H2···O4^i	0.880	2.073	2.901(3)	156.3
N3–H3···O4^i	0.880	2.055	2.872(3)	154.0
N4–H4···O4^i	0.880	2.222	2.968(2)	142.3
N5–H5···O3	0.880	2.332	3.133(3)	151.3
N6–H6···O3	0.880	2.253	3.072(3)	154.7
N7–H7···O2	0.880	2.100	2.966(4)	167.7
N8–H8···O1	0.880	2.034	2.868(3)	157.6

Symmetry code: (i) x+1/2, −y+1/2, z−1/2.

^{a} Riding hydrogen atoms generated by AFIX 43.

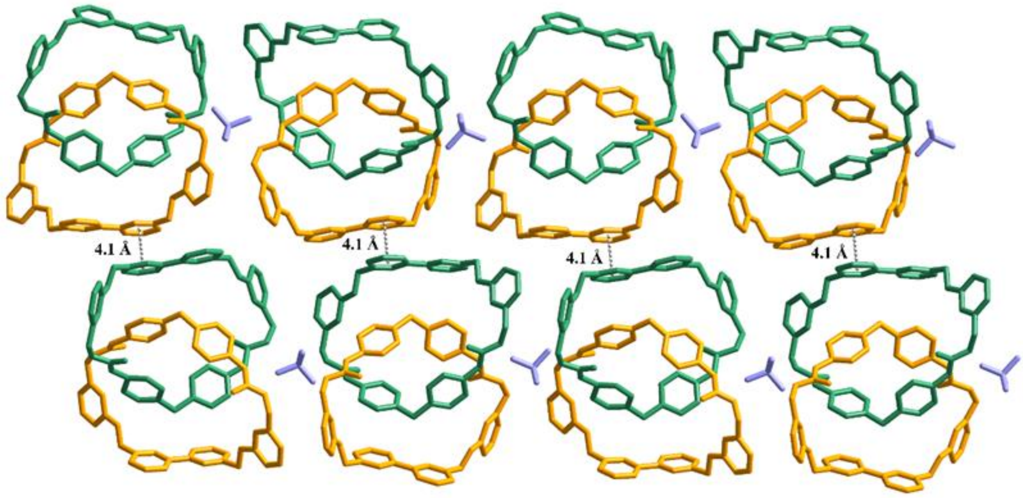

Supplementary Fig. 65. Arrangement of adjacent 1D strands with a distance of ~4.1 Å between the bpy units at the catenane exterior.

7. Guest-induced Mechanostereochemical Switching

$Ba(OTf)_2$ was purchased from commercial supplier (Energy, purity ≥ 98%). Tetramethylammonium cyanide (TMACN) was prepared by mixing tetramethylammonium chloride (TMACl) with 1 eq. of NaCN (purchased from Merck, purity ≥ 95%) in MeCN, followed by filtration and solvent removal. All salts were dried *in vacuo* and stored in a vacuum desiccator containing silica gel prior to use. Stock solutions of TBA_2SO_4 (50 mM), $Ba(OTf)_2$ (50 mM), $[Cu(MeCN)_4](PF_6)$ (50 mM) and TMACN (200 mM) were prepared in DMSO-d_6.

The guest-controlled mechanostereochemical switching of **C** was studied by the sequential addition of sulfate, barium, copper(I), cyanide and finally sulfate ions in DMSO-d_6 at room temperature. Structural changes were monitored by ^{1}H NMR after each guest addition. Aliquots of 10 μL of the TBA_2SO_4 stock solution was first added to a solution of **C** (500 μL, 1 mM), and no further spectral changes were observed after addition of 4 eq. of sulfate (total 40 μL of the sulfate stock solution). The sulfate ions in the host were then removed by addition of aliquots of 10 μL of the $Ba(OTf)_2$ stock solution. Guest-free **C** was completely re-generated after 4 eq. of Ba^{2+} has been introduced (total 40 μL of the barium stock solution). Aliquots of 5 μL of the $[Cu(MeCN)_4](PF_6)$ stock solution were then added, and a complete metalation of **C** was observed after 1 eq. of Cu^+ was introduced (total 10 μL of the copper(I) stock solution). No further spectral changes were found upon addition of a further 5 μL (0.5 eq.) of the copper(I) stock solution. To remove the Cu^+ ion from **C**, aliquots of 5 μL of the TMACN stock solution were added, and complete re-generation of **C** was observed after a total of 6 eq. of cyanide (total 15 μL of the TMACN stock solution) was added. Further switching to the chiral co-conformation by forming the sulfate complex was demonstrated by addition of further aliquots of the TBA_2SO_4 stock solution. Addition of a total of 5 eq. (50 μL) of the stock sulfate solution resulted in a ^{1}H NMR spectrum consistent to that of the sulfate complex with sharp signals.

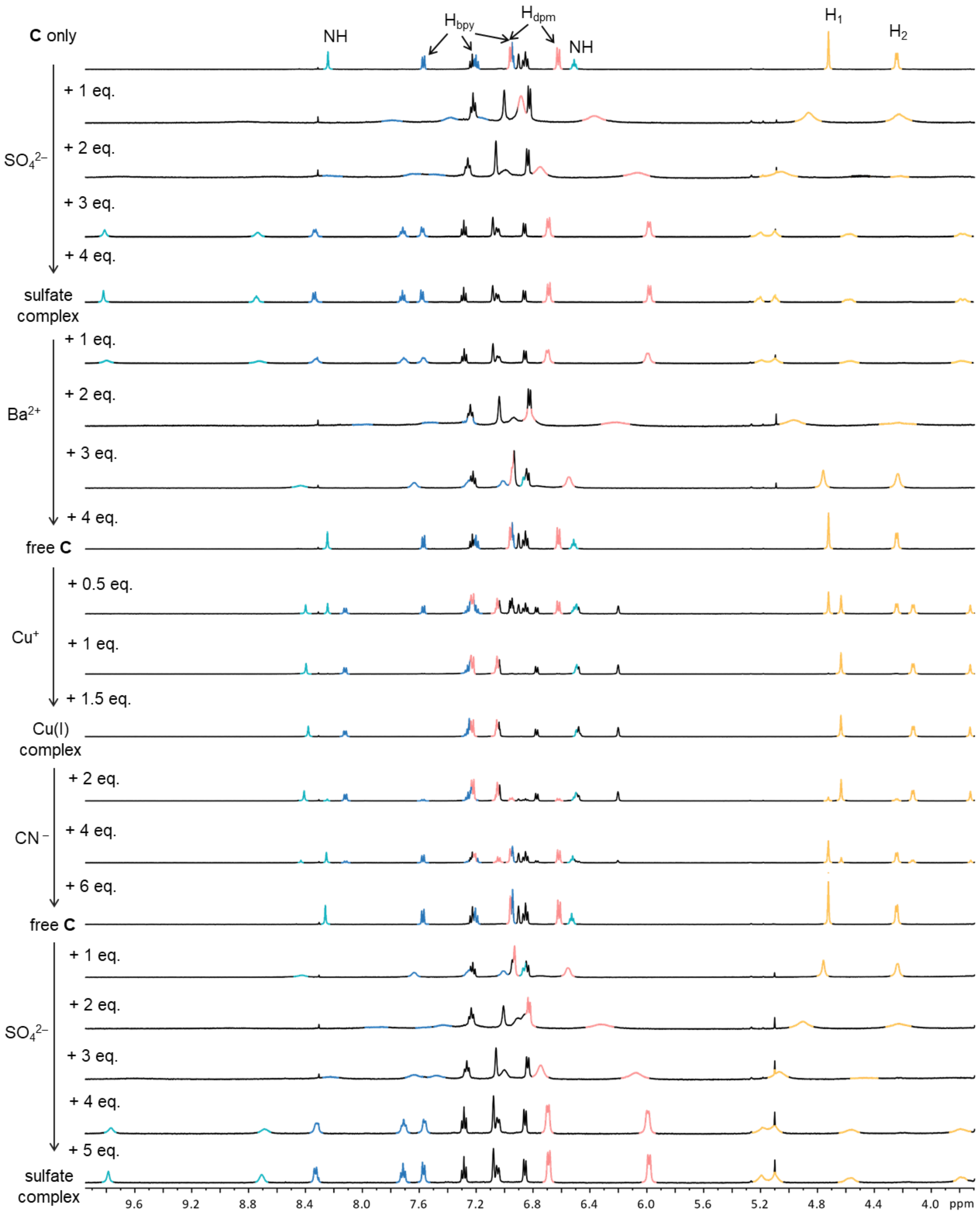

Supplementary Fig. 66. Partial ^{1}H NMR spectra (500 MHz, 298 K, DMSO-d_6) of **C** (1 mM) during the sequential addition and removal of sulfate and Cu(I) guests.

Supplementary References

1. Berná, J. *et al.* A catalytic palladium active-metal template pathway to [2]rotaxanes. *Angew. Chem. Int. Ed.* **46**, 5709–5713 (2007).
2. Price, J. R. et al. Copper(I) templated synthesis of a 2,2′-bipyridine derived 2-catenane: synthetic, modelling, and X-ray studies. *Aust. J. Chem.* **62**, 1014–1019 (2009).
3. Thordarson, P. Determining association constants from titration experiments in supramolecular chemistry. *Chem. Soc. Rev.* **40**, 1305–1323 (2011).
4. Bindfit. http://app.supramolecular.org/bindfit/.
5. Howe, E. N. W., Bhadbhade, M. & Thordarson, P. Cooperativity and complexity in the binding of anions and cations to a tetratopic ion-pair host. *J. Am. Chem. Soc.* **136**, 7505–7516 (2014).
6. Gacek, M. & Undheim, K. *N*-quaternary compounds—XXVIII: Circular dichroism of α-trimethylammonium acids. *Tetrahedron* **29**, 863–866 (1973).
7. Zuo, W. *et al.* Chirality sensing of choline derivatives by a triple anion helicate cage through induced circular dichroism. *Chem. Commun.* **54**, 7378–7381 (2018).
8. Hanwell, M. D. *et al.* Avogadro: an advanced semantic chemical editor, visualization, and analysis platform. *J. Cheminform.* **4**, 17 (2012).
9. Frisch, M. J. et al. *Gaussian 09, Revision A.02* (Gaussian, Inc., 2016).
10. Becke, A. D. Density-functional thermochemistry. III. The role of exact exchange. *J. Chem. Phys.* **98**, 5648–5652 (1993).
11. Lee, C., Yang, W. & Parr, R. G. Development of the Colle-Salvetti correlation-energy formula into a functional of the electron density. *Phys. Rev. B* **37**, 785–789 (1988).
12. Stephens, P. J., Devlin, F. J., Chabalowski, C. F. & Frisch, M. J. *Ab initio* Calculation of vibrational absorption and circular dichroism spectra using density functional force fields. *J. Phys. Chem.* **98**, 11623–11627 (1994).
13. Barone, V. & Cossi, M. Quantum calculation of molecular energies and energy gradients in solution by a conductor solvent model. *J. Phys. Chem. A* **102**, 1995–2001 (1998).
14. Cossi, M., Rega, N., Scalmani, G. & Barone, V. Energies, structures, and electronic properties of molecules in solution with the C-PCM solvation model. *J. Comput. Chem.* **24**, 669–681 (2003).
15. Sheldrick, G. M. *SHELXT* - Integrated space-group and crystal-structure determination. *Acta Cryst.* **A71**, 3–8 (2015).
16. Sheldrick, G. M. Crystal structure refinement with *SHELXL*. *Acta Cryst.* **C71**, 3–8 (2015).
